# Unified thalamic model generates multiple distinct oscillations with state-dependent entrainment by stimulation

**DOI:** 10.1371/journal.pcbi.1005797

**Published:** 2017-10-26

**Authors:** Guoshi Li, Craig S. Henriquez, Flavio Fröhlich

**Affiliations:** 1 Department of Psychiatry, University of North Carolina at Chapel Hill, Chapel Hill, NC, United States of America; 2 Department of Biomedical Engineering, Duke University, Durham, NC, United States of America; 3 Department of Biomedical Engineering, University of North Carolina at Chapel Hill, Chapel Hill, NC, United States of America; 4 Department of Cell Biology and Physiology, University of North Carolina at Chapel Hill, Chapel Hill, NC, United States of America; 5 Department of Neurology, University of North Carolina at Chapel Hill, Chapel Hill, NC, United States of America; 6 Neuroscience Center, University of North Carolina at Chapel Hill, Chapel Hill, NC, United States of America; École Normale Supérieure, College de France, CNRS, FRANCE

## Abstract

The thalamus plays a critical role in the genesis of thalamocortical oscillations, yet the underlying mechanisms remain elusive. To understand whether the isolated thalamus can generate multiple distinct oscillations, we developed a biophysical thalamic model to test the hypothesis that generation of and transition between distinct thalamic oscillations can be explained as a function of neuromodulation by acetylcholine (ACh) and norepinephrine (NE) and afferent synaptic excitation. Indeed, the model exhibited four distinct thalamic rhythms (delta, sleep spindle, alpha and gamma oscillations) that span the physiological states corresponding to different arousal levels from deep sleep to focused attention. Our simulation results indicate that generation of these distinct thalamic oscillations is a result of both intrinsic oscillatory cellular properties and specific network connectivity patterns. We then systematically varied the ACh/NE and input levels to generate a complete map of the different oscillatory states and their transitions. Lastly, we applied periodic stimulation to the thalamic network and found that entrainment of thalamic oscillations is highly state-dependent. Our results support the hypothesis that ACh/NE modulation and afferent excitation define thalamic oscillatory states and their response to brain stimulation. Our model proposes a broader and more central role of the thalamus in the genesis of multiple distinct thalamo-cortical rhythms than previously assumed.

## Author summary

Computational modeling has served as an important tool to understand the cellular and circuit mechanisms of thalamocortical oscillations. However, most of the existing thalamic models focus on only one particular oscillatory pattern such as alpha or spindle oscillations. Thus, it remains unclear whether the same thalamic circuitry on its own could generate all major oscillatory patterns and if so what mechanisms underlie the transition among these distinct states. Here we present a unified model of the thalamus that is capable of independently generating multiple distinct oscillations corresponding to different physiological conditions. We then mapped out the different thalamic oscillations by varying the ACh/NE modulatory level and input level systematically. Our simulation results offer a mechanistic understanding of thalamic oscillations and support the long standing notion of a thalamic “pacemaker”. It also suggests that pathological oscillations associated with neurological and psychiatric disorders may stem from malfunction of the thalamic circuitry.

## Introduction

The thalamocortical network plays a central role in cerebral rhythmic oscillations [1–4] and abnormal thalamocortical rhythms have been associated with disorders such as depression, schizophrenia and Alzheimer’s disease [5–7]. Understanding the cellular and circuit mechanisms of thalamocortical oscillations thus constitutes a crucial first step to comprehend the network impairments underlying neurological and psychiatric disorders. However, the mechanisms by which the thalamocortical network generates distinct states of oscillatory patterns remain highly debated [8–11]. One important question is whether the thalamus, originally believed to be the “pacemaker” of thalamocortical oscillations [7, 8, 12], is indeed able to independently generate multiple distinct brain rhythms or whether the thalamus requires interaction with the cortex [13–16]. Answering this question not only provides the basis for a mechanistic understanding of brain oscillations, but also will provide important insights in the design of effective mechanism-based brain stimulation techniques that specifically target abnormal thalamocortical dynamics.

Experimental evidence suggests that the thalamus is capable of independently generating multiple, distinct oscillatory states. In the cat lateral geniculate nucleus (LGN), *in vitro* and *in vivo* studies have identified a subset of thalamocortical cells (TCs) that generate high-threshold bursting at theta (θ) and alpha (α) frequency bands and thus may mediate the cellular mechanism of both θ and α oscillations [12, 17, 18]. Coupled with gap junctions [12, 17], high-threshold bursting TC cells provide synchronized excitatory inputs to local interneurons and reticular cells that entrain the majority of TC cells (i.e., non-high threshold bursting TC cells) into the α rhythm via feed-forward and feedback inhibition [18]. Besides θ/α oscillations, the thalamus is also critically involved in the genesis of the slow delta rhythm and spindle oscillations that appear at different stages of non-rapid eye movement (NREM) sleep [2, 19, 20]. Moreover, the thalamus is able to produce high frequency oscillations in both β and γ bands (20–60 Hz) in the neonatal rat whisker sensory system [21], during attentional processing in cats [22] and during cognitive tasks in humans [23]. Consistently, experimental data indicated that fast rhythms (30–40 Hz) could be synchronized with an intrathalamic mechanism [24]. It is not known whether the same neural substrate and circuitry for θ and α oscillations could also mediate other oscillatory patterns and what controls the transition among these oscillatory states.

Thalamic processing is subject to the action of modulatory neurotransmitters including acetylcholine (ACh), norepinephrine (NE), serotonin (5-HT), histamine (HA) and dopamine (DA) [25]. Of these neurotransmitters, cholinergic and noradrenergic modulation plays the key and best understood role in shaping the oscillatory state of the thalamocortical network [26]. Consistently, the α rhythm is both induced by muscarinic cholinergic receptor activation in slices of cat LGN [17] and supported by cholinergic innervation *in vivo* [18]. Fast rhythmic activities in the β/γ frequency band (20–60 Hz) are observed in cat thalamocortical cells [11] and promoted by cholinergic projection from the brainstem [27]. Besides, slow δ oscillations are believed to be mediated by hyperpolarization of TC neurons with diminished activation of both the cholinergic and noradrenergic systems [28, 29]. In addition to neuromodulation, the specific type of thalamic oscillation is also dependent on the level of afferent excitation. For example, the amplitude of the α oscillation is maximal when the eyes are closed and therefore input form the retina is low [30]. Hence, generation and transition of distinct thalamic oscillations depend critically on neuromodulation and afferent excitation, yet a unified model has been lacking so far.

To close this gap, we developed a biophysical, conductance-based model of the thalamic network constrained by extensive experimental data to test the hypothesis that generation and transition of distinct thalamic oscillations are functions of both ACh/NE neuromodulation and afferent excitation under various physiological conditions. By varying only these two model parameters, ACh/NE neuromodulation and afferent excitation, we demonstrated that the thalamic network is capable of generating multiple distinct oscillatory states (δ, spindle, α/θ and γ/β oscillations) in absence of cortical input. We elucidated the cellular and circuit mechanisms for each oscillatory state by manipulating the network connectivity and key model parameters. Simulation results suggest that generation of distinct thalamic oscillations is a result of both intrinsic oscillatory cellular properties and specific network connectivity patterns. The manifestation of multiple distinct oscillations in one unified biophysical thalamic model enabled us to examine the impact of rhythmic stimulation on thalamic network dynamics. By applying periodic stimulation to the thalamic model during three major oscillatory states (δ, α and γ oscillations), we observed that entrainment of thalamic oscillations is highly state-dependent in that the same stimulation induced much stronger and more prominent entrainment during γ oscillations than δ and α oscillations due to the different oscillatory mechanisms. Our findings emphasize the importance of considering the rich role of endogenous oscillations in thalamus for the study of thalamo-cortical rhythms and highlight the need to consider the network state when modulating brain oscillations with periodic stimulation waveforms.

## Results

**Fig 1:**
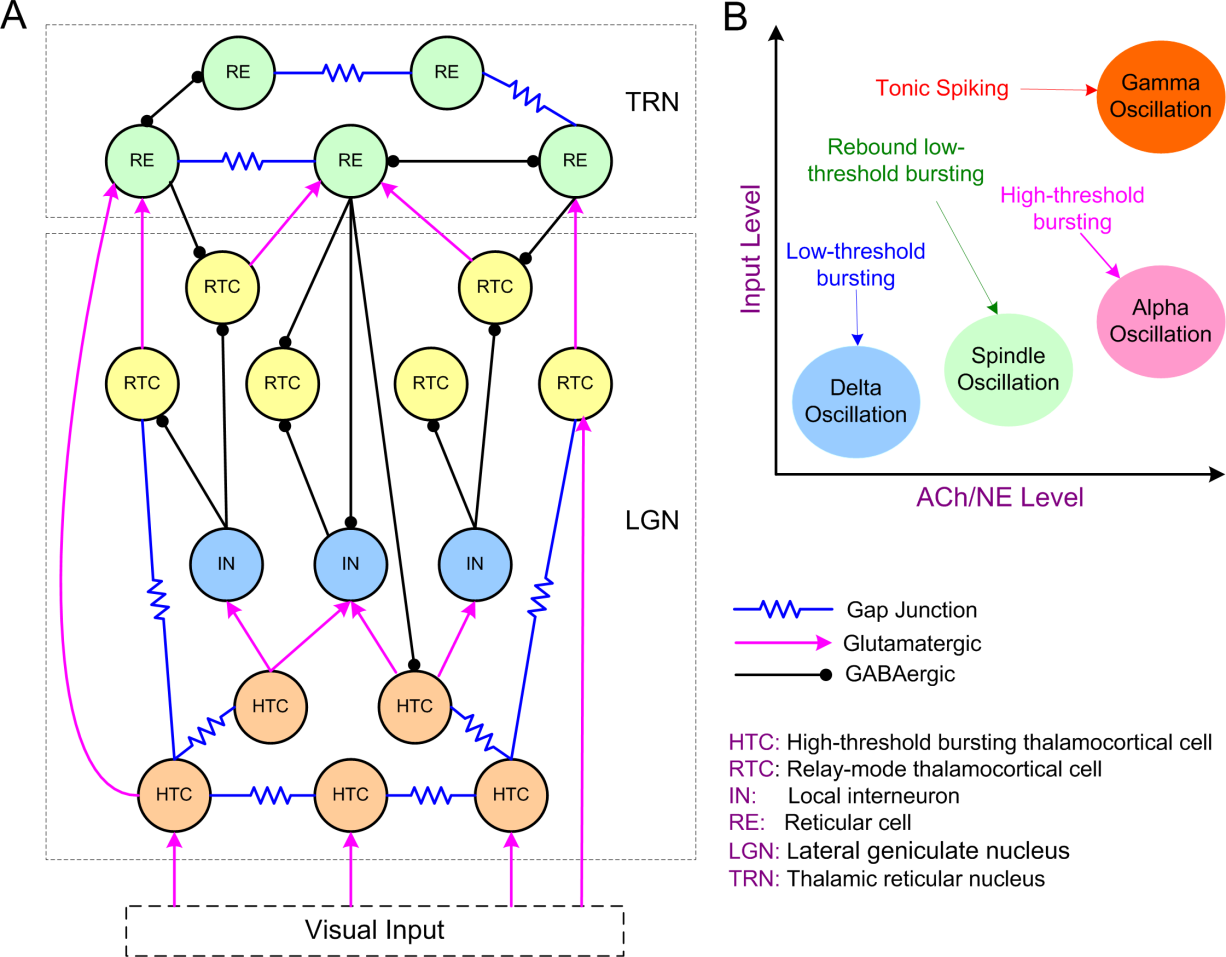
The thalamic network structure and model hypothesis. **(A)** Schematic diagram of the thalamic network model. **(B)** Model hypothesis: Generation and transition of thalamic oscillations are functions of both ACh/NE modulation and afferent excitation. Delta oscillations occur in the low ACh/NE modulatory state with minimal afferent input; spindle oscillations exist in the medium ACh/NE modulatory state with slightly increased afferent input; alpha oscillations are generated in the high ACh/NE modulatory state with weak afferent input, while gamma oscillations are generated in the high ACh/NE modulatory state with strong afferent input. The labels with arrows indicate the firing patterns of the high-threshold bursting thalamocortical cells in different oscillatory states.

We developed a biophysical conductance-based thalamic network model containing both the lateral geniculate nucleus (LGN) and the reticular nucleus (TRN) of the thalamus (Fig 1A; see Methods). We first constructed single cell models of high-threshold bursting TC cells (HTCs), relay-mode TC cells (RTCs), local interneurons (INs) and thalamic reticular cells (REs) that replicated experimentally observed firing patterns both in control condition and in case of modulation by ACh/NE (S1 and S2 Figs; S1 Text). By connecting the four types of neurons into a thalamic network (Fig 1A), we tested the central hypothesis that generation and transition of distinct thalamic oscillations are functions of both ACh/NE modulation and afferent excitation (Fig 1B).

### The thalamic network is able to produce multiple distinct oscillatory states dependent on ACh/NE modulation and afferent excitation

By varying the potassium leak conductance modulated by ACh/NE and the maximal input conductance corresponding to different levels of afferent excitation in all four types of thalamic neurons (Table 1), we were able to generate four distinct oscillatory states (δ, spindle, α, and γ oscillations) in the thalamic network. In the model, the increasing level of ACh/NE during the transition from deep sleep to wakefulness corresponded to lower potassium leak conductance in HTC, RTC and RE cells, but higher potassium leak conductance in INs (Table 1; also see Methods). All four types of thalamic neurons received random Poisson distributed inputs mediated by AMPA receptors and the maximal input conductance was a fixed constant value associated with the AMPA synaptic channels.

**Table 1:** Model parameters for different oscillatory states.

ACh/NE Level	Network Condition	Oscillatory State	g_KL_ (mS/cm^2^) (nS)			g_input_ (nS)		
			HTC & RTC	IN	RE	HTC & RTC	IN	RE
**Low**	Deep sleep	δ	0.035 (10.2)	0.01 (1.7)	0.03 (4.3)	0.1	0.1	0.1
**Medium**	Light sleep	Spindle	0.01 (2.9)	0.015 (2.6)	0.02 (2.9)	0.3	0.3	0.3
**High**	Awake (eyes closed)	α	0.0 (0.0)	0.02 (3.4)	0.01 (1.4)	1.5	1.5	1.5
	Awake (eyes opening + attention)	γ	0.0 (0.0)	0.02 (3.4)	0.01 (1.4)	17	1.5	1.5

#### Delta oscillations

**Fig 2:**
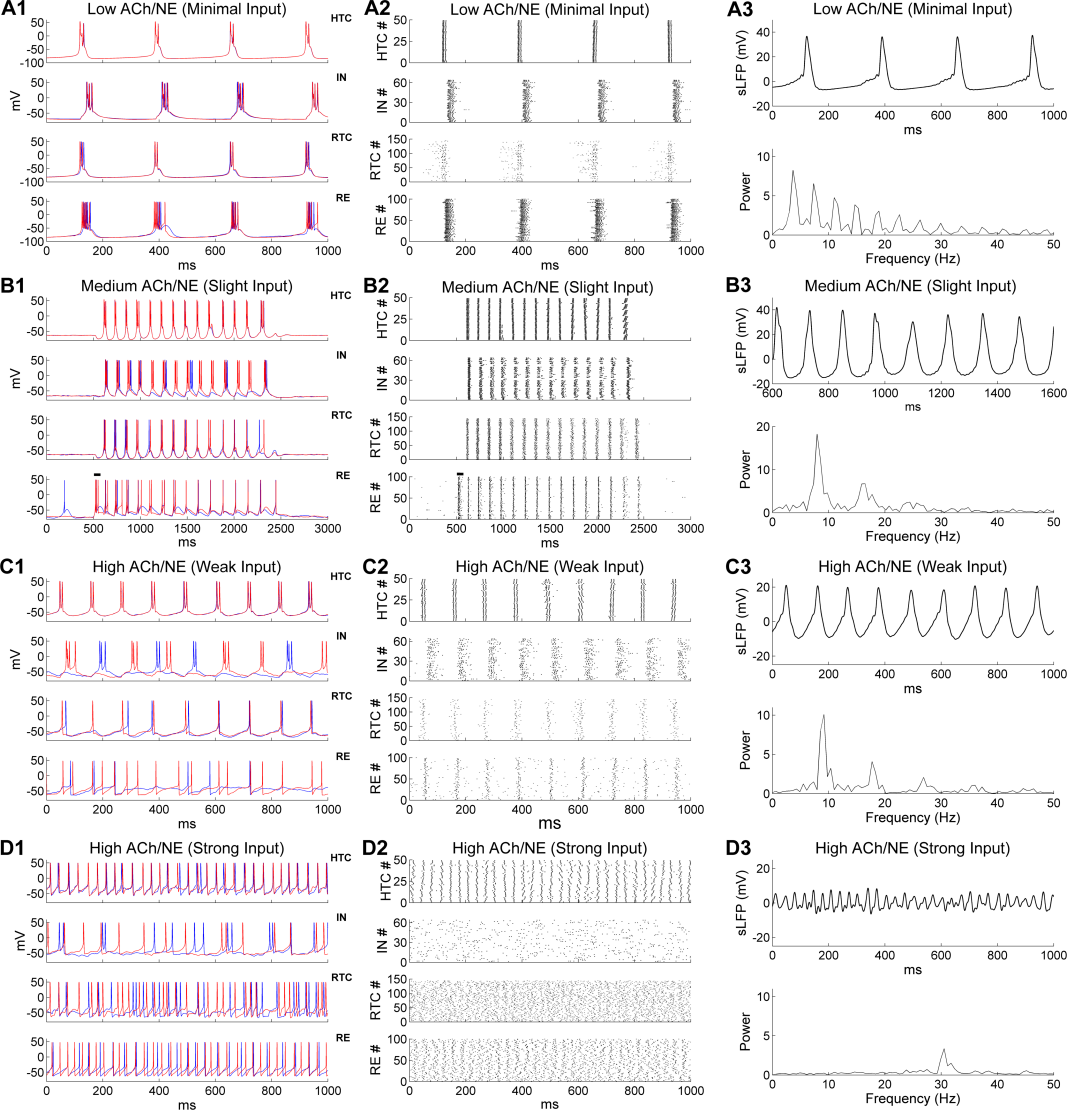
Generation of multiple distinct oscillations in the thalamic network model under three different levels of ACh/NE modulation and four different levels of afferent excitation. **The parameters used in different oscillatory states are shown in Table 1. (A)** Generation of δ oscillations in the low ACh/NE modulatory state and with minimal afferent excitation. **(A1)** Voltage traces of two representative HTC, IN, RTC and RE cells each. **(A2)** Spike rastergrams of HTC, IN, RTC and RE cells. **(A3)** Simulated LFP (*top*) with associated frequency power spectrum (*bottom*). **(B)** Generation of spindle oscillations in the medium ACh/NE modulatory state and with slight afferent excitation. The black horizontal bars in (**B1-B2**) indicate the injection of a transient current input (100 ms × 100 pA) into RE neurons to trigger spindle oscillations. **(C)** Generation of α oscillations in the high ACh/NE modulatory state and with weak afferent excitation. **(D)** Generation of γ oscillations in the high ACh/NE modulatory state and with strong afferent excitation.

For modeling deep sleep (i.e., non-rapid eye movement sleep, NREM 3), both the ACh/NE modulation and afferent inputs were set to very low values (Table 1). In this case, our model generated a low frequency oscillation at 3.7 Hz (Fig 2A), which was within the δ frequency band (1–4 Hz; [19, 29, 31, 32]). The oscillation frequency was reduced to around 3 Hz if the potassium leak conductance in TC cells increased slightly (S3A Fig), and further reduced to around 2 Hz when the inactivation time constant of the low-threshold T-type Ca^2+^ current (*I*_Ca/T_) increased 25% combined with increase of the potassium leak current and substantial blockage of the regular leak current (S3B Fig). Thus, the specific δ frequency can be fine-tuned by the leak/potassium leak conductance and the dynamics of *I*_Ca/T_. During δ oscillation, all four types of neurons fired low-threshold bursts (LTBs) at membrane potentials lower than or close to -70 mV and were well synchronized (Fig 2A1). Some additional firing of RTC cells was observed before the start of each δ cycle but not later in the cycle due to inhibition from both IN and RE neurons (Fig 2A2, *lower middle*). The inter-burst frequency (3.7 Hz) was close to the spontaneous LTB frequencies of isolated TC cells in the low ACh/NE modulation state (S1 Fig), suggesting that δ oscillation was predominantly generated by the intrinsic bursting properties of TC neurons under hyperpolarized condition [29, 31]. Both the IN and RE neurons fired strong bursts of action potentials following HTC and RTC cells as they received excitatory inputs from TC neurons. IN and RE inhibition did not produce rebound bursting in TC cells because of the hyperpolarized membrane potential (around -82 mV). The highly synchronized network activity gave rise to strong simulated local field potential (sLFP) oscillation at the same bursting frequency (3.7 Hz; Fig 2A3, *top;* spectrogram, *bottom*). Therefore δ oscillations can be generated locally in the thalamus during low ACh/NE modulation in case of low levels of afferent excitation, a physiological condition achieved during deep sleep [19].

#### Spindle oscillations

In the network condition simulating light sleep (i.e., NREM 2), we increased both the ACh/NE modulation level (i.e., medium ACh/NE modulation) and the random background input (Table 1). With increased ACh/NE modulation, the potassium leak conductance in TC cells decreased (Table 1). As a result the membrane potential of TC cells was less hyperpolarized and thus they no longer generated LTBs (due to inactivation of the low-threshold T-type Ca^2+^ current at a resting potential between -62 and -65 mV; Fig 2B1, *top and lower middle*), in line with the simulation results of isolated TC neurons (S1 Fig). Under such condition, when RE neurons were activated by a transient afferent input (100 ms × 100 pA, simulating a cortical UP state, indicated by the horizontal bars in Fig 2B1 and 2B2), a train of oscillation cycles were triggered that lasted for about 2 seconds (Fig 2B1), resembling thalamic spindles during light sleep [2, 33]. Transient activation of RE neurons hyperpolarized both HTC and RTC cells, producing rebound LTBs which in turn drove IN and RE burst firing that then initiated the next oscillation cycle. This back-and-forth interaction ended when RE firing eventually failed to evoke rebound LTBs in TC cells due to short-term synaptic depression (Fig 2B1; also see below). At a population level, all four types of model neurons were highly synchronized during spindle oscillations (Fig 2B2). Compared with the δ oscillations, the number of spikes per burst in RE neurons was greatly reduced due to depolarization of membrane potentials with decreased potassium leak conductance (compare Fig 2B2 with Fig 2A2, *bottom*). Also, random sparse spontaneous RE firing was not able to trigger spindle oscillations as it was not strong enough to hyperpolarize TC cells to evoke rebound bursting (Fig 2B2, first and last 500 ms). Thus, spindle oscillation was a result of both intrinsic properties of TC cells (rebound bursts after hyperpolarization; S1 Fig) and network interaction (TC-RE interplay). The simulated LFP exhibited strong periodicity with an oscillation frequency of 7.9 Hz (Fig 2B3), within the spindle frequency range (7–15 Hz; [20]). Our simulation results are consistent with a number of experimental findings: (1) spindle oscillations occur at more depolarized membrane potentials (between -65 and -55 mV) of TC cells than δ oscillations (−68 and −90 mV; [31]); (2) spindle oscillations are generated by synaptic interaction of TC and RE neurons [2, 3, 34]; and (3) stimulation of cholinergic release suppresses δ oscillations by depolarizing TC neurons [29].

Since the effects of NE on INs are not known [25, 26], we considered the modulatory effect of ACh only (on INs) in the above simulation (i.e., increase of *g*_*KL*_ from δ to spindle oscillations; Table 1). To account for other possible effects of NE on INs, we either fixed *g*_*KL*_ (the excitatory effect of NE counteracts the inhibitory effect of ACh) or decreased *g*_*KL*_ (the excitatory effect of NE overcomes the inhibitory effect of ACh) during the transition from deep sleep to light sleep. We found that both the duration and power of spindle activity increased slightly if *g*_*KL*_ was fixed at 0.01 mS/cm^2^ (controls: 0.015 mS/cm^2^) as IN bursting became more robust (S4B Fig). By comparison, if *g*_*KL*_ decreased to 0.005 mS/cm^2^, the spindle duration and power reduced slightly because the high level of uncorrelated IN background spiking substantially suppressed RTC bursting (S4C Fig). Overall, spindle oscillations were not significantly affected by considering other possible NE effects on INs.

#### Alpha oscillations

We modeled the increased ACh/NE levels of the waking state by completely blocking the potassium leak current in TC cells, reducing it in RE neurons, and increasing it in INs (Table 1). The synaptic weight of the random Poisson inputs to the network also increased considerably from the light sleep stage (from 0.3 nS to 1.5 nS; Table 1), simulating an overall increase of random background inputs in the awake state. With these parameter values, the thalamic network displayed prominent α oscillations (Fig 2C). Similar to isolated HTC cells in the high ACh/NE modulation state (S1 Fig), HTC cells in the network generated high-threshold bursts (HTBs) in the α frequency band and were highly synchronized because of gap junctions (Fig 2C1, *top*; [12, 17]). The synchronized HTBs strongly excited INs and produced corresponding HTBs in INs (Fig 2C1, *upper middle*). Subsequently, these HTBs in the INs provided pronounced phasic inhibition on RTC cells. Once recovered from IN and RE inhibition, RTC cells fired single action potentials (APs) at relatively depolarized membrane potentials (> -65 mV) and were strongly synchronized (Fig 2C1, *lower middle*). The synchronized HTC/RTC inputs drove correlated firing in RE neurons that provided feedback inhibition to both HTC and RTC cells (Fig 2C1, *bottom*). At the population level, a high level of synchrony was observed in all four types of neurons in the network (Fig 2C2). Of note, both HTC and IN neurons fired bursts of APs whereas both RTC and RE neurons fired tonic spiking (Fig 2C1 and 2C2). The sLFP showed strong rhythmic structure (Fig 2C3, *top*) and the frequency power spectrum revealed a dominant peak at 9.2 Hz (Fig 2C3, *bottom*), close to the α frequency (8.9 ± 1.2 Hz) recorded from freely moving cats during natural wakefulness [18]. Hence, our simulation results are consistent with recent experimental observations that thalamic α oscillations are mediated by high-threshold bursting of HTC cells under muscarinic cholinergic receptor activation [17, 18]. Moreover, a high level of phase locking between RE spikes and α rhythm was not required for coherent α oscillations (S7 Fig; [18]) while blocking the gap junctions among TC cells (HTC-HTC & HTC-RTC connections) seriously disrupted the α rhythm in the thalamic network (S8 Fig), as observed experimentally [12, 35].

We also examined other possible effects of NE on INs during alpha oscillations (S5 Fig). If the potassium leak conductance in INs was maintained at the level for modeling deep sleep (*g*_*KL*_ = 0.01 mS/cm^2^), RTC activities were suppressed considerably compared to controls (*g*_*KL*_ = 0.02 mS/cm^2^) due to substantial increase of spontaneous IN firing (S5B Fig). Consequently, the oscillation power decreased moderately (S5B Fig). On the other hand, if the potassium leak conductance in INs was reduced to 0 mS/cm^2^ due to NE action, the high level spontaneous firing of INs almost completely suppressed RTC responses leading to little RE activities. Without effective RE feedback inhibition, HTC cells switched from bursting to single spiking or became inactive because of the inactivation of the high-threshold T-type Ca^2+^ channel (S5C Fig). As a result, the α oscillation was seriously disrupted. Thus, the model predicts that inhibition of INs by ACh and/or NE will increase the robustness of α oscillations.

#### Gamma oscillations

**Fig 3:**
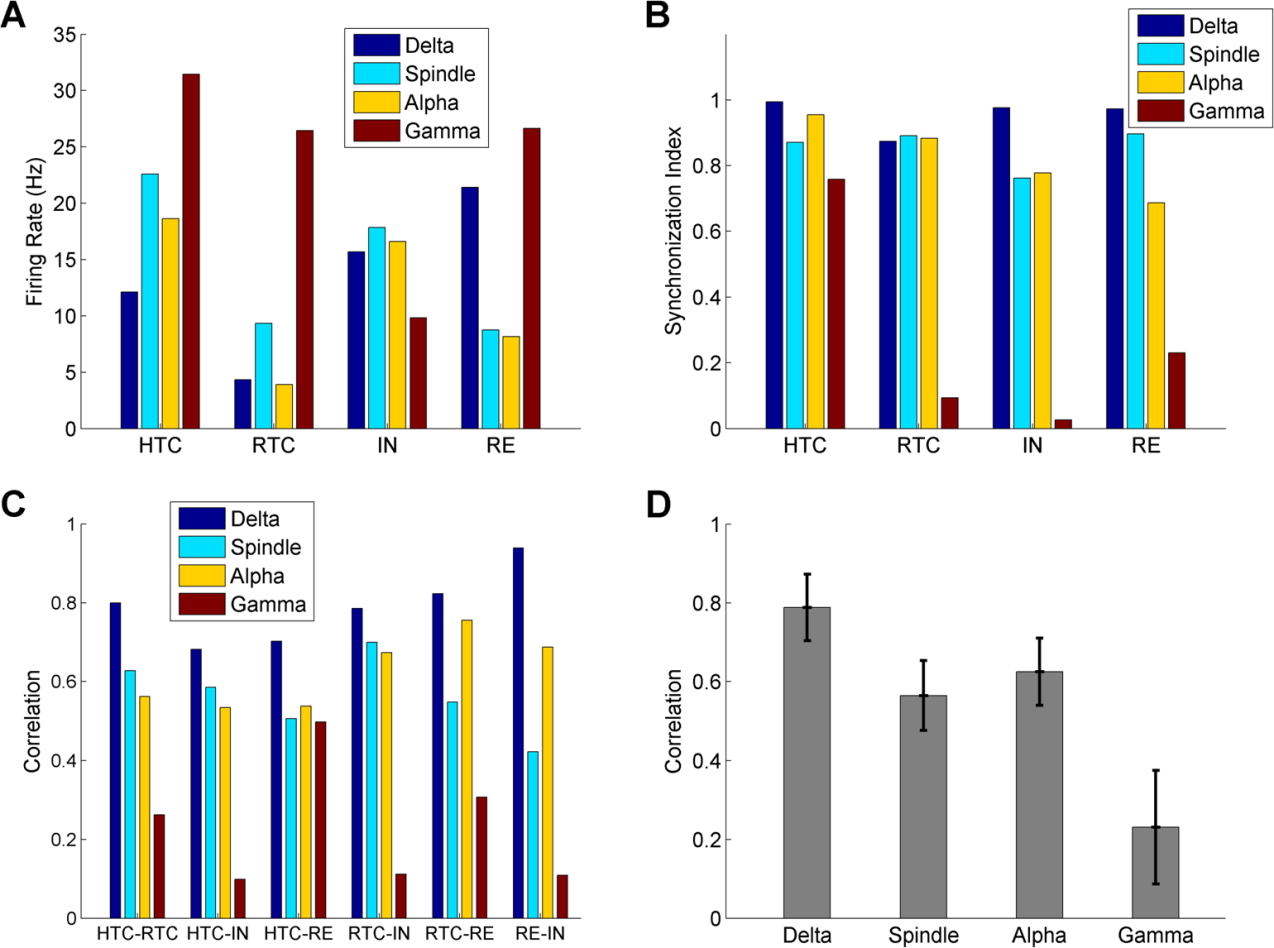
Quantification of thalamic network activity during four oscillatory states (δ, spindle, α and γ oscillations). Average firing rates of HTC, RTC, IN and RE neurons across four oscillatory states. (A) Synchronization index of HTC, RTC, IN and RE neurons across four oscillatory states (B) Cross-correlation between different groups of thalamic neurons during four oscillatory states. (C) Average cross-correlation of six pairs of neuronal groups (**C**) during four oscillatory states. Error bars indicate standard deviation.

In the high ACh/NE modulation state, when the maximal synaptic conductance of the random Poisson inputs to TC cells was substantially increased (from 1.5 nS during α oscillations to 17 nS; Table 1) to mimic the condition of eyes opening and additional intracortical input during focused attention, endogenous α oscillations in the thalamic network gave way to γ oscillations (Fig 2D). With much stronger synaptic excitation, the average firing rates of both HTC and RTC cells increased substantially (HTC, α: 18.6 Hz, γ: 31.4 Hz; RTC, α: 3.9 Hz, γ: 26.4 Hz; Fig 3A). Notably, HTC cells switched from HTBs (during α oscillations) to tonic spiking while remaining well synchronized because of the gap junction connections (Fig 2D1 and 2D2, *top*; also Fig 3B). In contrast, synchrony was not detectable in either the RTC voltage traces (Fig 2D1, *lower middle*) or spike rastergram (Fig 2D2, *lower middle*), demonstrating that the firing of RTC cells started to lose rhythmicity as the random inputs greatly increased (Fig 3B). Different from α oscillations, INs fired a mix of single spikes and bursts in a random fashion (Fig 2D1 and 2D2, *upper middle*). This was because compared with HTBs, single HTC spikes were less potent in driving IN bursts and with higher HTC firing rates, the strength of the HTC→IN synapses was reduced more by short-term depression (STD). Indeed, the IN spikes became synchronized and phase-locked to the γ rhythm when the STD at the HTC→IN synapses was removed (S9 Fig). As the excitatory inputs from HTC and RTC cells increased, the average firing rate of RE neurons also went up considerably (α: 8.2 Hz; γ: 26.7 Hz; Fig 3A). Unlike INs, weak synchrony was still observed in RE voltage traces (Fig 2D1, *bottom*) and the population spike rastergram (Fig 2D2, *bottom*), owing to inter-RE gap junctions and inhibition. With reduced network synchronization, the amplitude of the sLFP oscillations decreased substantially compared with lower frequency oscillations (δ, spindle and α) (compare Fig 2D3 with Fig 2A3–2C3, *top*). The frequency power spectrum revealed a dominant peak at 30.5 Hz, within the low gamma frequency band (Fig 2D3, *bottom*). Our simulation results are in agreement with experimental data that TC cells are capable of generating fast rhythmic spiking activities in γ frequency band in response to strong depolarizing inputs [11, 27, 36]. In particular, the depolarization-induced transition from high-threshold bursting to high-frequency tonic spiking in HTC cells closely matches the experimental data (see Fig 2B of [27]). In addition, if the synaptic drive to TC cells reduced, the thalamic network oscillated at a lower frequency in the β band (e.g., 23.2 Hz; S10 Fig). This suggests that thalamic β and γ oscillations may share the same cellular mechanism and they constitute a continuous range of “fast” oscillation frequency dependent on depolarization, as suggested experimentally [37]. Lastly, there was little change in γ oscillation frequency or power when the potassium leak conductance in INs was maintained at the level used to model deep sleep (*g*_*KL*_ = 0.01 mS/cm^2^) or blocked completely (*g*_*KL*_ = 0.0 mS/cm^2^), though IN spikes became more synchronized with reduced level of the potassium leak current (S6 Fig). This suggests that altering the potassium leak current in INs has differential effects on different oscillatory states (see above).

### Quantification of thalamic network activity during four oscillatory states

To quantify the network activity during the four oscillatory states, we calculated the average firing rates and synchronization index of four different groups of thalamic neurons (HTC, RTC, IN & RE, Fig 3A and 3B) along with the correlation between different neuronal populations (Fig 3C and 3D). Overall, the average firing rates of RTC cells were lower than HTC cells for all oscillatory states, because RTC cells were less excitable than HTC cells due to a much smaller high-threshold T-type Ca^2+^ current (*I*_Ca/HT_, S1 Table). In addition, RTC cells received inhibition from both INs and REs, while HTC cells received inhibition from REs only (Fig 1A). The firing rates of both HTC and RTC cells increased from δ to spindle oscillations and decreased during α oscillations followed by a large increase during γ oscillations (Fig 3A). This is because the bursting frequency of TC cells increased from δ to spindle oscillations (δ: 3.7 Hz; spindle: 7.9 Hz) and during the transition from spindle to α oscillations, the oscillation frequency was similar (spindle: 7.9 Hz; α: 9.2 Hz), but RTC cells switched from bursting to single spiking while the number of spikes per burst reduced in HTC cells (spindle: 3.2; α: 2.1). The TC firing rates were highest during γ oscillations among all four oscillatory states since TC cells received strong afferent drive (Table 1). During the transition from γ to α oscillations, the firing rates of INs increased (γ: 9.8 Hz; α: 16.6 Hz; Fig 3A) while the firing rates of REs reduced (γ: 26.6 Hz; α: 8.2 Hz; Fig 3A). This is consistent with the experimental observation that LGN interneurons in cats exhibited an increase in firing rate during α oscillations (compared to non-α state; presumably γ state in our model), whereas TRN neurons showed a decrease in firing rate [18]. Our simulations suggest that such differential effects resulted from the following three factors: (1) INs were less excitable than RE cells in the high ACh/NE state owing to a larger potassium leak conductance (Table 1); (2) INs received inputs from HTCs only while REs received inputs from both HTC and RTC cells (Fig 1A). Consequently, the large increase in RTC firing during γ oscillations led to substantial increase in RE spikes; (3) HTC cells switched from HTBs during α oscillations to tonic spiking during γ oscillations, which reduced the effectiveness of excitatory drive on INs, as mentioned earlier. Combined with STD, INs switched from strong bursting to irregular mix of bursting and tonic spiking (compare Fig 2C1 with Fig 2D1, *upper middle*). As a result, the IN firing rate reduced during γ oscillations compared with α oscillations.

The generation of distinct oscillations in the thalamic network depends on synchronization of different neuronal populations. To evaluate the degree of neuronal synchrony in the thalamic network, we calculated the synchronization index (*SI*) of the four neuronal populations during different oscillatory states (Fig 3B). The *SI* of both HTC and RTC cells maintained at relatively high level during δ, spindle and α oscillations (> 0.87; Fig 3B). During γ oscillations, the *SI* of RTC cells substantially decreased to 0.09, while that of HTC cells only slightly reduced to 0.76 (Fig 3B) because of gap junctions. The *SI* of INs decreased moderately from δ to spindle/α oscillations and reduced greatly during γ oscillations (δ: 0.98; spindle: 0.76; α: 0.78; γ: 0.03; Fig 3B). Similarly, the *SI* of REs showed a decreasing trend from δ to γ oscillations, but the reduction during γ oscillations was smaller (δ: 0.97; spindle: 0.90; α: 0.69; γ: 0.23; Fig 3B) due to the inter-RE gap junctions and inhibition. These results indicate that the thalamic network had the highest level of synchrony during δ oscillations followed by spindle and α oscillations and the synchronization level was the lowest during γ oscillations.

We next computed the cross-correlation between different groups of neuronal populations during the four oscillatory states (Fig 3C). First, the correlation was consistently highest during δ oscillations and lowest during γ oscillations for all six neuronal population pairs. Second, the correlation during spindle oscillations was either comparable to (e.g., HTC-RTC) or moderately lower than (e.g., RTC-RE) α oscillations (Fig 3C). Lastly, the correlation between HTC cells and RE neurons during γ oscillations was substantially higher than other population pairs (Fig 3C), consistent with higher level of synchrony of these two neuronal assembles during γ oscillations (Fig 3B). On average, the neuronal correlation was largest during δ oscillations followed by α and spindle oscillations and the correlation was lowest during γ oscillations (Fig 3D).

Overall, as the ACh/NE modulation level and synaptic input increased, the thalamic network switched from low frequency oscillations (δ oscillations) to higher frequency oscillations (spindle and α oscillations), and to fast frequency oscillations (γ oscillations). Correspondingly, the membrane potentials of TC neurons gradually depolarized and the firing patterns of HTC cells switched from LTBs to rebound LTBs to HTBs and to tonic spiking, while those of RTC neurons changed from LTBs to rebound LTBs and to tonic spiking. Hence, with minimal change of parameters (the potassium leak conductance and the synaptic input strength; Table 1), the thalamic network was able to generate multiple distinct and stable oscillatory states that appear under different behavioral and cognitive conditions [2, 18, 19, 22, 23]. Next, we dissected the cellular and circuit mechanisms for each oscillatory state by manipulating the major network connectivity and varying key model parameters.

### Delta oscillations are synchronized by both gap junctions and synaptic inhibition

**Fig 4:**
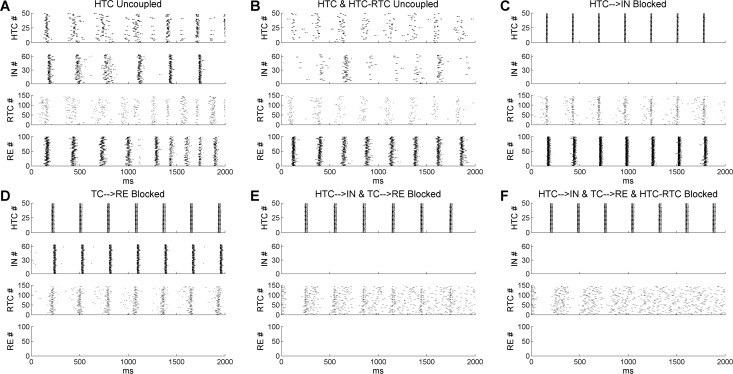
Thalamic δ oscillations are synchronized by both gap junctions and synaptic inhibition from IN and RE neurons. **(A)** Rastergrams of HTC, IN, RTC and RE cells when the gap junctions among HTC cells are removed. **(B)** Gap junctions among HTC cells and between HTC and RTC cells are removed. **(C)** Projection from HTC cells to IN neurons is removed. **(D)** Projection from TC cells to RE neurons is removed. **(E)** Projection from TC cells to both IN and RE neurons is removed. **(F)** Projection from TC cells to both IN and RE neurons is removed and the gap junctions between HTC and RTC cells are blocked.

To understand how δ oscillations were generated in the thalamic network, we first removed the gap junction connections among HTC cells. Removal of gap junctions led to large variation in HTC burst timing as the LTB frequencies of individual cells differed from each other because of intrinsic heterogeneity (i.e., different leak conductance) and external noise input (Fig 4A, *top*). It also reduced RTC synchrony through the HTC-RTC gap junctions (compare Fig 4A with Fig 2A2, *lower middle*). Consequently, after a few partially synchronized δ cycles, both HTC and RTC cells broke into two subpopulations separated by the IN and RE inhibition. In one cycle, a majority of HTC cells burst with a small percentage of RTC cells while in the next cycle, a majority of RTC cells burst with a small percentage of HTC cells (Fig 4A). As a result, the network oscillation frequency doubled to about 6.7 Hz (S11 Fig). Interestingly, if the HTC-RTC gap junctions were additionally removed, the frequency doubling effect was not observed (Fig 4B) and the network oscillation frequency just slightly increased to about 4 Hz (S11 Fig). Although the δ rhythm was maintained without any TC gap junctions, the degree of network synchrony was substantially reduced compared with the control condition (compare Fig 4B with Fig 2A2).

To examine the role of synaptic inhibition in thalamic network synchronization, we first blocked the HTC→IN projections to remove the feedforward IN inhibition on RTC cells (gap junctions intact). We found that RTC cells remained well synchronized (Fig 4C). We next blocked the TC→RE connections to eliminate the RE feedback inhibition on TC cells. Similarly, RTC bursting was still well phase-locked to the δ rhythm (Fig 4D). If, however, both the HTC→IN and TC→RE connections were blocked, RTC bursts became largely desynchronized, except the subset of RTC cells that formed gap junctions with HTC cells (Fig 4E). This suggests that either IN or RE inhibition is required for the synchronization of RTC cells. Lastly, when the HTC-RTC gap junctions were additionally removed (besides IN and RE inhibition), RTC bursting became completely desynchronized (Fig 4F). Therefore, thalamic δ oscillations are generated intrinsically by TC cells and are synchronized by both gap junctions and synaptic inhibition.

### Spindle oscillations are mainly generated through TC-RE interaction

**Fig 5:**
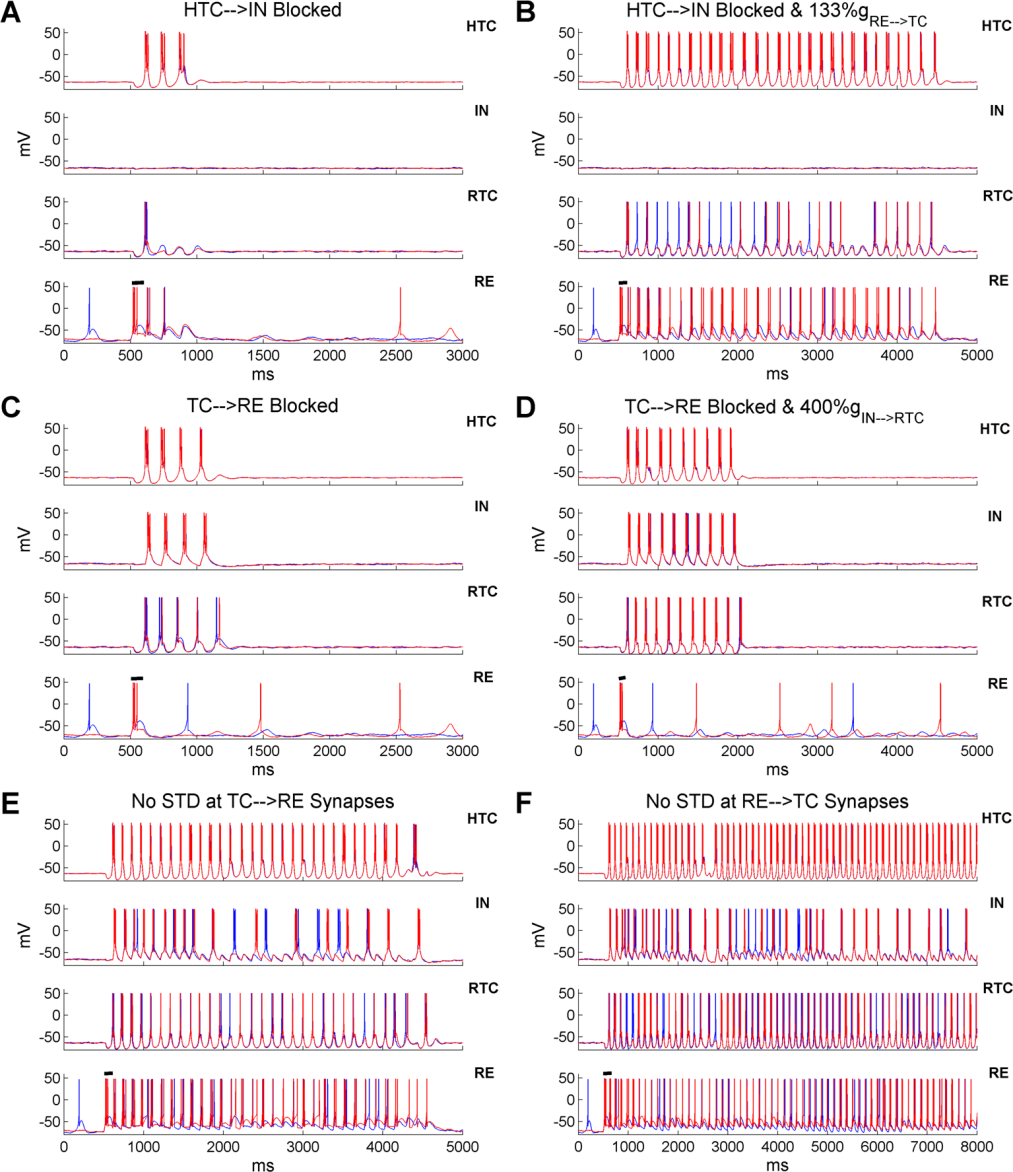
Spindle oscillations are mainly generated through the TC-RE interaction and the spindle duration is tightly regulated by RE inhibition. **(A)** Voltage traces of two representative HTC, IN, RTC and RE cells each when the excitatory inputs from HTC cells to IN neurons are blocked. Black horizontal bar indicates the presence of transient current injection (100 ms) into RE neurons to trigger spindles (same for below). **(B)** Excitatory inputs from HTC cells to IN neurons are blocked and the maximal synaptic conductance of the inhibitory RE→TC synapses increased 33% (from 3 nS to 4 nS). **(C)** Excitatory inputs from TC cells to RE neurons are blocked. **(D)** Excitatory inputs from TC cells to RE neurons are blocked and the maximal synaptic conductance of the inhibitory IN→RTC synapses increases fourfold (from 3 nS to 12 nS). **(E)** STD at the TC→RE synapses is blocked. **(F)** STD at the RE→TC synapses is blocked.

In the thalamic network, spindle oscillations were triggered by transient synchronized RE burst firing which produced rebound LTBs in TC cells. Besides RE inhibition, RTC cells also received IN inhibition which could elicit rebound LTBs. To differentiate the role of IN and RE inhibition in spindle oscillations, we blocked the HTC→IN and TC→RE projections, respectively, to eliminate IN and RE bursts. When IN burst firing was removed, HTC and RTC cells fired only three and one bursts respectively (Fig 5A), indicating that IN inhibition contributed to spindle oscillations by hyperpolarizing RTC cells. Nevertheless, the lack of IN inhibition could be easily compensated by increased inhibition from RE neurons. In the absence of IN inhibition, when the inhibitory RE→TC synaptic weight increased only 33% (from 3 nS to 4 nS), spindle oscillations persisted for about 4 seconds, twice the duration of the control case (compare Fig 5B with Fig 2B1). Thus, with sufficient RE inhibition, the TC-RE feedback loop was able to create and sustain spindle oscillations for a few seconds. On the other hand, when the TC→RE synapses were blocked, the TC-IN network generated four cycles of spindle oscillations before termination (Fig 5C). This was because the IN bursts hyperpolarized RTC cells resulting in rebound RTC bursts. At the same time, RTC hyperpolarization facilitated rebound bursts in HTC cells through the HTC-RTC gap junctions, which further drove IN bursting. Nevertheless, in the absence of RE inhibition, the inhibitory IN→RTC synaptic weight needed to increase fourfold (from 3 nS to 12 nS) in order to sustain spindles for about 1.5 seconds (Fig 5D). This contrasted to a much more prominent increase in spindle duration induced by a much smaller increase of the RE→TC synaptic weight (compare Fig 5D with Fig 5B). Such difference suggests that RE inhibition plays a more major role than IN inhibition in generating spindle oscillations, consistent with experimental data [3, 20, 38].

Similar to the effect of increased RE→TC synaptic weight, when the TC→RE synaptic weight increased 50% (AMPA: from 4 nS to 6 nS; NMDA: from 2 nS to 3 nS), RE neurons responded to TC inputs with more burst spikes and the duration of spindle oscillations increased substantially from about 2 seconds to 3.8 seconds (S12 Fig). Hence, spindle duration was fine-tuned by the strength of RE inhibition. As RE inhibition gradually decreased over the course of spindles due to short-term depression (STD), we hypothesized that removing STD at the TC→RE or RE→TC synapses could significantly extend or even sustain spindles indefinitely. Indeed, when STD at the TC→RE synapses was removed, spindle oscillations were sustained for about 4 seconds (Fig 5E), whereas they continued beyond 7 seconds when STD at the RE→TC synapses was abolished (Fig 5F). Thus in our model, spindle oscillations are terminated mainly by STD at the inhibitory RE→TC synapses, as suggested previously [39]. Our results are consistent with a recent experimental study showing that the duration of spindle oscillations depends critically on the inhibitory strength of RE neurons on TC cells [38]. Moreover, it suggests that the variable spindle duration observed in experiments may be a result of difference in RE excitability and heterogeneous synaptic strength between TC and RE neurons.

### Thalamic α oscillations are sculpted by high-threshold bursting dynamics of HTC cells

**Fig 6:**
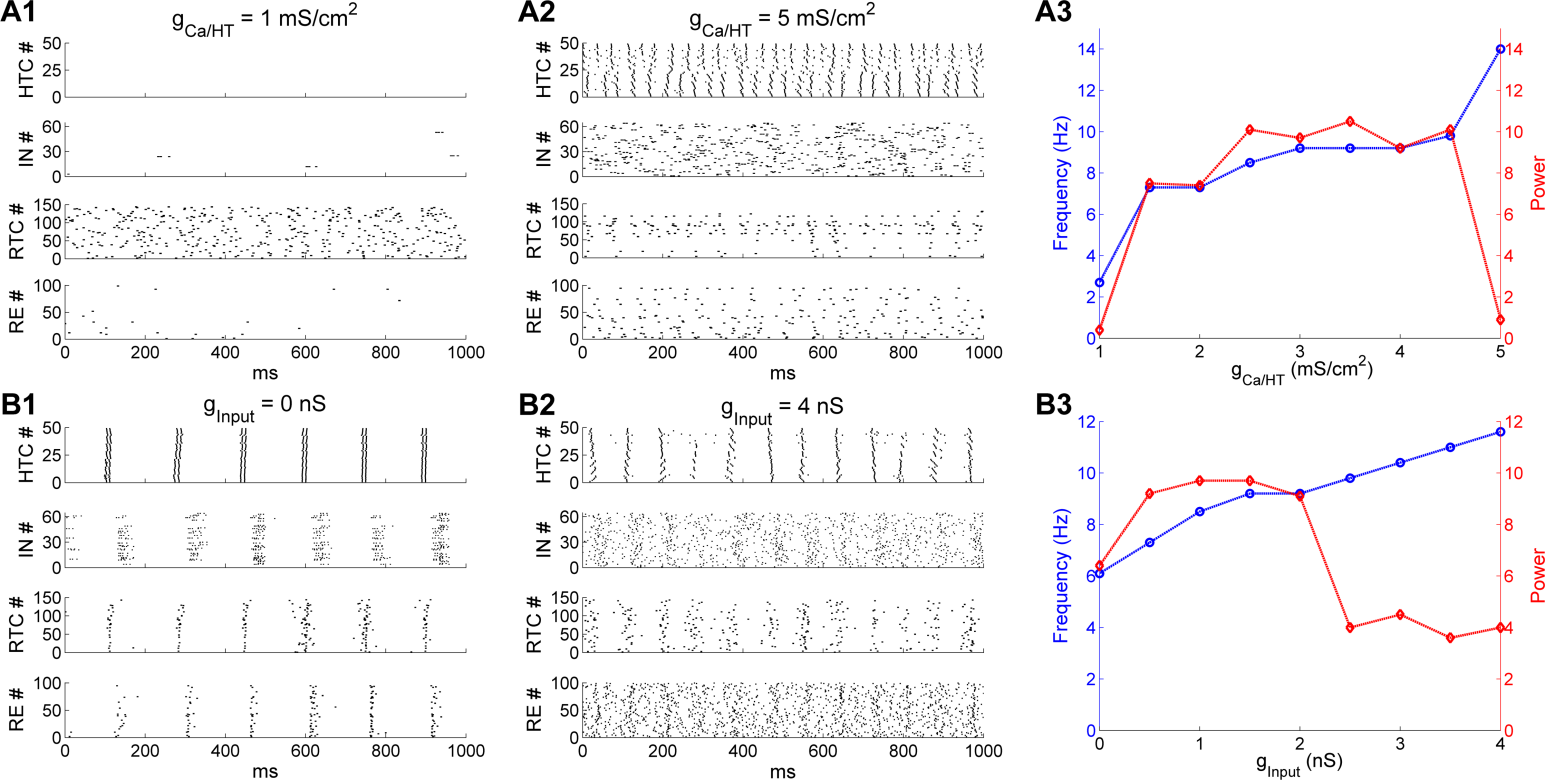
Thalamic α oscillations are sculpted by the high-threshold bursting dynamics of HTC cells. **(A**) Effect of varying the maximal conductance density of the high-threshold T-type Ca^2+^ current (g_Ca/HT_) in HTC cells. **(A1)** Spike rastergrams of HTC, IN, RTC and RE cells when g_Ca/HT_ is reduced to 1 mS/cm^2^. **(A2)** g_Ca/HT_ is increased to 5 mS/cm^2^. The default value is 3 mS/cm^2^. **(A3)** Dominant network oscillation frequency (blue) and spectral peak power (red) when g_Ca/HT_ varies from 1 mS/cm^2^ to 5 mS/cm^2^. **(B)** Effect of varying the maximal input conductance to all thalamic cells. **(B1)** Spike rastergrams of HTC, IN, RTC and RE cells when the maximal input conductance is reduced to 0 nS. **(B2)** Maximal input conductance increases to 4 nS. The default value is 1.5 nS. **(B3)** Dominant network oscillation frequency (blue) and spectral peak power (red) when the maximal input conductance varies from 0 nS to 4 nS.

Experimental data has identified the HTC cell as an important neuronal substrate for thalamic θ and α oscillations [12, 17, 18]. To test the importance of HTBs in generating α oscillations, we varied the maximal conductance density of the high-threshold T-type Ca^2+^ current (g_Ca/HT_) in HTC cells from 1 mS/cm^2^ to 5 mS/cm^2^ (default: 3 mS/cm^2^). When g_Ca/HT_ was reduced to 1 mS/cm^2^, HTC cells failed to produce HTBs (Fig 6A1, *top*). With a lack of excitation from HTC cells, IN neurons were mostly silent (Fig 6A1, *upper middle*). In the absence of feedforward inhibition from IN neurons, RTC cells fired random spontaneous activities at about 3 Hz without synchronization (Fig 6A1, *lower middle*). As a result, the α rhythm disappeared. On the other hand, when g_Ca/HT_ was increased to 5 mS/cm^2^, depolarized HTC cells started to transition from the bursting mode to the tonic spiking mode and became desynchronized because of strong heterogeneous bursting dynamics (Fig 6A2, *top*). Consequently, INs fired random bursts (Fig 6A2, *upper middle*) and suppressed the activity of RTC cells (Fig 6A2, *lower middle*). As a result, no synchronized α oscillations developed. These simulation results demonstrated that synchronized HTBs of HTC cells were essential for the generation of thalamic α oscillations, consistent with experimental observations [12, 17]. Fig 6A3 plots the network oscillation frequency (blue) and the spectral peak power (red) as a function of g_Ca/HT_. When g_Ca/HT_ increased from 1.5 to 4.5 mS/cm^2^, the oscillation frequency moderately increased from 7.3 Hz to 9.8 Hz while the oscillation power remained relatively stable. At the values of 1 and 5 mS/cm^2^, there was a large drop in oscillation power because no synchronized α oscillations occurred. Hence, thalamic α oscillations are limited to a relatively narrow frequency band (8–10 Hz) where HTC cells fired robust high-threshold bursting.

The HTB frequency of HTC cells is also dependent on the depolarization level (S1 Fig; [7, 17]). As such, changing the external drive to the network would alter the frequency of α oscillations. Indeed, when the random afferent inputs were removed from the thalamic network, HTC cells still fired spontaneous synchronized HTBs, but at a lower frequency (6 Hz; Fig 6B1, *top*). Subsequently, the whole network was synchronized at about 6 Hz (Fig 6B1), which was within the θ frequency band. One the other hand, when the maximal synaptic input conductance to the whole network increased to 4 nS (default: 1.5 nS), the HTB frequency of HTC cells increased to about 12 Hz, but there was only one spike per burst (Fig 6B2, *top*) due to inactivation of the *I*_Ca/HT_ current. The random activity of both IN and RE neurons increased substantially because of reduced synchronized HTC excitation and increased random input drive, which substantially suppressed RTC firing (compare Fig 6B2 with Fig 2C2, *lower middle*). As a result, the α oscillation power was significantly reduced (Fig 6B3). The network oscillation frequency (blue) and power (red) as a function of the maximal input conductance (g_input_) are shown in Fig 6B3. As g_input_ increased from 0 to 4 nS, the oscillation frequency increased monotonically from 6.1 Hz to 11.6 Hz. By comparison, the oscillation power increased initially from 0 nS to 0.5 nS and stayed in the same level for values up to 2 nS before decreasing considerably for larger input strength. Therefore, reducing the excitatory drive to the thalamic network switched α oscillations to θ oscillations while increasing the excitation level moved it to the upper α frequency band, consistent with experimental observation that HTC cells could underlie both α and θ oscillations dependent on the depolarization level of HTC cells [7, 12]. Nevertheless, although stronger depolarization of HTC cells increased α frequency, it reduced the α power by switching high-threshold bursting to tonic spiking (Fig 6A2 and 6B2). Consequently, thalamic α oscillations exhibit optimal power between 8–10 Hz (Fig 6A3 and 6B3). Our results thus explain why α oscillations decrease with more afferent excitation (e.g., eyes opening; [30]).

### HTC gap junctions are required for thalamic γ oscillations in the absence of strong RE feedback inhibition

**Fig 7:**
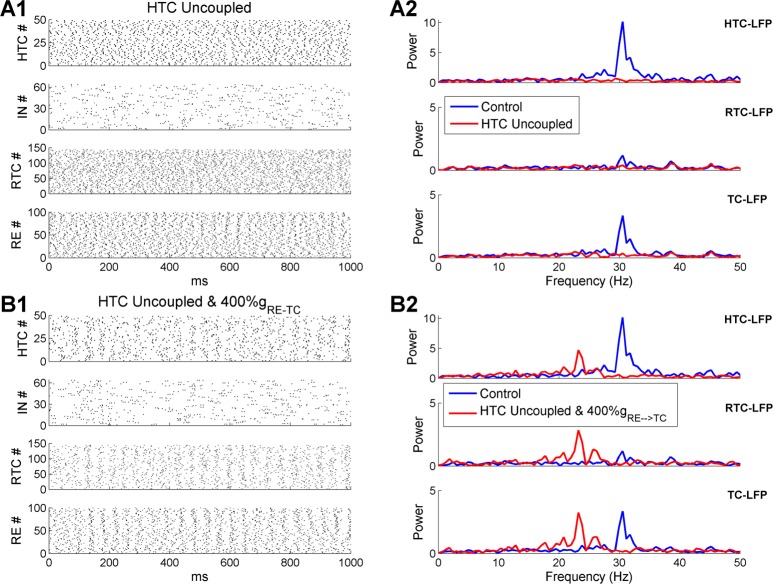
HTC gap junctions are required for thalamic γ oscillations in the absence of extra-strong feedback RE inhibition. **(A)** Thalamic γ oscillations are eliminated when the gap junctions among HTC cells are removed. **(A1)** Spike rastergrams of HTC, IN, RTC and RE cells. **(A2)** Frequency power spectrum of sLFP from HTC cells (*top*), RTC cells (*middle*) and combined HTC and RTC cells (*bottom*) in the control (blue) and HTC uncoupled (red) conditions, respectively. **(B)** The RE→TC synaptic weight needs to increase four-fold to generate similar γ oscillations in the absence of gap junctions among HTC cells. **(B1-B2)** As **(A1-A2),** but with fourfold increase of the RE→TC synaptic weight.

The key for γ oscillations was that HTC cells maintained a high level of synchrony even in the presence of strong random afferent inputs (Fig 2D2, *top*). The HTC rhythmicity was propagated to RE neurons via excitatory projection and to RTC cells via gap junctions. RE synchrony was boosted by inter-RE gap junctions and inhibition, which moderately constrained RTC firing through the RE→RTC inhibitory synapses. If the gap junctions between HTC cells were removed during γ oscillations, HTC cells were completely desynchronized (Fig 7A1, *top*) leading to unconstrained RE and RTC firing (Fig 7A1, *lower middle and bottom*). As a result, the sLFP spectral peak of either HTC or RTC cells was eliminated (Fig 7A2). Thus, gap junctions may play a critical role in synchronizing γ oscillations in the thalamus, as in the hippocampus [40] and neocortex [41].

Besides gap junctions, the negative feedback loop between excitatory (e.g., pyramidal cells) and inhibitory neurons also plays an important role in fast oscillation synchronization [42, 43]. To examine whether the feedback interaction between TC and RE cells could sustain γ oscillations as well, we increased the maximal RE→TC synaptic conductance fourfold (default: 3 nS; 4-fold increase: 12 nS) in the absence of HTC gap junction coupling. With much stronger RE inhibition, moderate degree of population rhythmicity emerged from the HTC, RTC and RE neurons (Fig 7B1). The sLFP frequency spectra revealed peaks of similar amplitude as controls, but at a lower frequency (controls: 30.5 Hz; 4-fold increase of RE inhibitory strength: 23.2 Hz; Fig 7B2) because of increased RE inhibition. Also, without HTC gap junctions, the amplitude of spectral peaks was similar for both HTC and RTC cells since they had similar degree of spike synchronization (Fig 7B1, *top and lower middle*). This was in contrast to the control case where the spectral peak of HTC-sLFP was much higher than that of RTC-sLFP (Fig 7B2). As a slight increase of the RE inhibitory synaptic weight significantly prolongs spindle oscillations (Fig 5B), such strong RE feedback inhibition (i.e., 4-fold increase of synaptic strength) seems unlikely in the thalamic network. Therefore, we conclude that HTC gap junctions are required for the synchronization of thalamic γ oscillations.

### Oscillatory state transition as a function of ACh/NE modulation and afferent excitation

**Fig 8:**
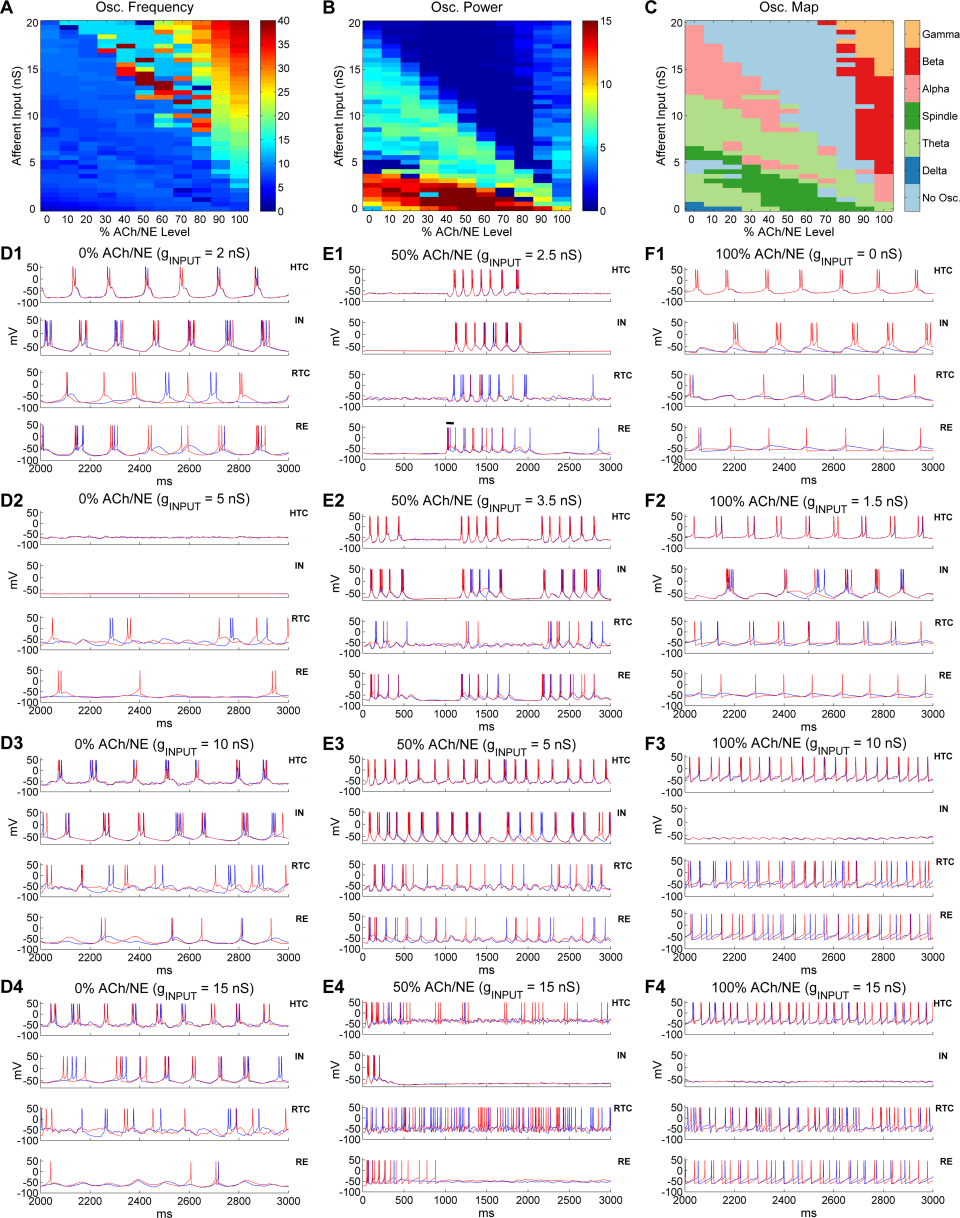
Oscillatory state transition as a function of ACh/NE modulation and afferent input. (A)Oscillation frequency heat map as a function of ACh/NE modulation and afferent input. (B) Oscillation power (spectral peak) heat map as a function of ACh/NE modulation and afferent input. (C) Distribution of different oscillatory states as a function of ACh/NE modulation and afferent input. (D) Voltage traces of two representative HTC, IN, RTC and RE neurons each under low ACh/NE modulation (0%) at four different levels of afferent input (**D1:** 2 nS; **D2:** 5 nS; **D3:** 10 nS; **D4:** 15 nS). (E) Voltage traces of two representative HTC, IN, RTC and RE neurons each under medium ACh/NE modulation (50%) at four different levels of afferent input (**E1:** 2.5 nS; **E2:** 3.5 nS; **E3:** 5 nS; **E4:** 15 nS). The horizontal bar in (**E1**) indicates the injection of a transient current input (100 ms × 100 pA) into RE neurons to trigger spindle oscillations. (F)Voltage traces of two representative HTC, IN, RTC and RE neurons each under high ACh/NE modulation (100%) at four different levels of afferent input (**F1:** 0 nS; **F2:** 1.5 nS; **F3:** 10 nS; **F4:** 15 nS). Note the scale difference in x axis between (**E**) and (**D**, **F**).

So far we have demonstrated that the thalamic network was able to generate multiple distinct oscillations dependent on ACh/NE modulation and afferent excitation. To further examine how the thalamic network transitions from one state to the other on a continuous basis, we divided the ACh/NE modulation into 11 levels evenly ranging from 0% to 100% and varied the maximal input conductance to TC cells from 0 up to 20 nS with a 0.5 nS step, which resulted in 451 different parameter combinations (see Methods section). We then simulated the thalamic network with all possible combinations of ACh/NE and input levels and identified the oscillatory state of the network across the entire parameter space. The network oscillation frequency and spectral power heat maps are shown in Fig 8A and 8B respectively. The networks with higher oscillation frequency (> 15 Hz) were predominantly located above the principal diagonal of the 2D parameter space (Fig 8A) indicating that the network oscillated faster with higher levels of ACh/NE modulation and afferent excitation. The highest oscillation frequency with the maximal level of ACh/NE (100%) and input (20 nS) was 38 Hz (note some oscillation frequencies close to 40 Hz right above the principal diagonal, but these network states were classified as non-oscillatory due to low oscillation power; see below). By comparison, the networks with higher oscillation power were primarily located below the principal diagonal with the maximal power residing in the bottom of the 2D space, corresponding to weak afferent excitation (Fig 8B). This suggests that oscillations driven minimally by the afferent input (e.g., spindle oscillations) are stronger than oscillations driven mostly by afferent excitation (e.g., γ oscillations). In addition, the oscillation power was minimal (< 1) above the principal diagonal except for high levels of ACh/NE modulation (> 70%).

Next, we determined the oscillatory state of the thalamic network under all examined combinations of ACh/NE and input levels and plotted the prominent oscillatory regimes across the entire parameter space (Fig 8C). Consistent with our modeling hypothesis (Fig 1B) and pervious analysis, we observed slow δ oscillations with low ACh/NE modulation (< 30%) and minimal afferent excitation (< 1 nS), spindle oscillations with medium ACh/NE modulation (20%-80%) and relatively weak afferent input (< 5 nS), and α, β and γ oscillations under high ACh/NE modulation (> 70%) with weak, moderate and strong inputs respectively (Fig 8C). The spindle oscillations under medium ACh/NE modulation were induced by a transient input to RE neurons as mentioned above (e.g., Fig 8E1) and the spindle regime moved to lower input area as the ACh/NE level increased (Fig 8C). For example, spindle appeared at 3 nS with 20% ACh/NE compared with 0 nS with 50% ACh/NE. This was because with low ACh/NE, TC cells fired spontaneous low-threshold bursts (LTBs) so higher afferent input was required to inactivate the low-threshold Ca^2+^ current to generate the state that permitted the occurrence of spindles. By comparison, for higher ACh/NE levels (50%-90%), TC cells no longer generated spontaneous LTBs and the network was relatively quiet without or with little afferent input (e.g., Fig 2B1). In addition to the externally-induced spindles (e.g., triggered by a transient input to RE neurons), we observed spontaneous spindle oscillations under low ACh/NE modulation (< 30%) and with higher level of afferent input (4.5–6.5 nS; Fig 8C). Such spontaneous spindle oscillations were induced spontaneously by the random afferent input and usually lasted for a few cycles (S13 Fig). Also, besides α oscillations at high ACh/NE modulation (100%), there were prominent α oscillations under low or medium ACh/NE modulation (< 60%) and with relatively large afferent input (> 8 nS). This was because large random afferent input activated the high-threshold bursting dynamics in HTC cells (see below). The region of such low-modulation α oscillations shifted to lower input area and became narrower as the ACh/NE level increased (Fig 8C; pink area in the left side). There were also sporadic α oscillations right above the region of spindles. During the transition from δ to spindles and from spindles to α oscillations, there existed two prominent θ oscillation regions (light green area): the first one appeared in the lower left corner while the second one started at higher input levels (7–12 nS) with 0% ACh/NE and gradually decreased to 0 nS with 100% ACh/NE, indicating lower afferent input was needed to drive the network to θ oscillations as ACh/NE increased. Besides the prime oscillatory regimes, there was also non-oscillatory area in the parameter map (light blue area in Fig 8C) characterized by very low oscillation power (Fig 8B). The first major non-oscillatory region was relatively narrow and appeared between the low-input θ oscillations and spontaneous spindle oscillations under low ACh/NE modulation (< 30%). By comparison, the second major non-oscillatory region was much wider which occurred above the principal diagonal extending from 0% up to 80% ACh/NE. One important characteristic of this non-oscillatory region was that it started at the highest input level (20 nS) without ACh/NE modulation (i.e., %0 ACh/NE) and expanded into lower input area as the ACh/NE modulation increased (Fig 8C, upper light blue area).

To understand the cellular basis of such oscillatory state transitions, we plotted the voltage traces of representative thalamic neurons with different afferent input drive at three levels of ACh/NE modulation (0%, 50% and 100%; Fig 8D–8F). As shown earlier, the thalamic network generated slow δ oscillations (1–4 Hz) with minimal input (e.g., 0.1 nS) in the low ACh/NE condition (Fig 2A). When the afferent input slightly increased (e.g., 2 nS), the LTB frequency of TC cells increased to the θ band (4–8 Hz; Fig 8D1), which underlay θ oscillations. Thus, model simulation suggested that both δ and θ oscillations could be mediated by the LTBs of TC cells depending on the input. With further increase of the afferent drive (e.g., 5 nS), the network entered the non-oscillatory state where HTC cells stopped bursting and RTC cells burst sparsely (Fig 8D2). This was because as the membrane depolarization increased, the low-threshold T-type Ca^2+^ current started to inactivate, reducing the intrinsic bursting dynamics of TC cells. Consequently, the spontaneous HTC bursts driven by random Poisson inputs were not able to induce synchronous bursts (only burstlets) but after-hyperpolarization in neighboring cells through gap junctions. As a result, the coupled HTC cells eventually became silent. Indeed, when the gap junctions were blocked, HTC cells were able to burst randomly (S14 Fig). When the afferent drive increased beyond 6.5 nS (e.g., 10 nS), HTC cells started to burst synchronously again owing to the activation of the high-threshold T-type Ca^2+^ current and the bursting frequency went into the θ band again (7.3 Hz, Fig 8D3). As the afferent input increased further (e.g., 15 nS), the burst frequency of HTC cells rose to the α band (9.2 Hz, Fig 8D4). At the highest afferent input tested (20 nS), HTC cells switched from HTBs to spare tonic spiking because of inactivation of the high-threshold T-type Ca^2+^ current and the network became desynchronized in the presence of strong random inputs (S15 Fig). To recapitulate, at the lowest level of ACh/NE (0%), as the afferent input increased, the thalamic network switched from slow δ oscillations to slow θ oscillations both mediated by the LTBs of TC cells. After a brief non-oscillatory state, the network exhibited spontaneous spindle oscillations followed by θ and α oscillations mediated by HTBs of HTC cells. The network eventually became desynchronized with strong afferent drive.

With medium level of ACh/NE modulation (50%), the thalamic network exhibited externally- induced spindle activity for relatively weak input (0–3 nS; Fig 8E1, also Fig 2B). The duration of spindles reduced with afferent excitation (S16 Fig) because depolarization increased the inactivation of the low-threshold Ca^2+^ current in TC cells making rebound LTBs less effective. Increasing the afferent input to 3.5 nS generated intermittent θ or spontaneous spindle-like oscillations with an oscillation frequency of 7.9 Hz (Fig 8E2). Specifically, the network driven by random Poisson inputs burst spontaneously for about 500 ms and stopped for about 500 ms before bursting again. Since the inter-burst interval (~500 ms) was relatively short, such network behavior was classified as intermittent or transient θ/α oscillations. When the afferent input further increased to 5 nS, the network generated continuous oscillations in the θ band (~ 6 Hz) which was mediated by HTBs of HTC cells (Fig 8E3). With large afferent drive (e.g., 15 nS), HTC cells switched from periodic HTBs to random sparse firing and the network became desynchronized (Fig 8E4). Similar to the non-oscillatory state in the low ACh/NE and high input condition (0% ACh/NE, 20 nS input; S15 Fig), the network desynchronization was caused by the inactivation of the high-threshold T-type Ca^2+^ current in the presence of random input drive (i.e., the gap junctions were no longer able to synchronize HTC cells with sparse random spikes). Notably, with medium ACh/NE modulation, the network entered the desynchronizing state at a much lower input level (0% ACh/NE: 20 nS; 50% ACh/NE: 9.5 nS; Fig 8C). This was due to the fact that with higher ACh/NE, the high-threshold T-type Ca^2+^ current started to inactivate at smaller input intensity. As a result, the non-oscillatory state expanded into lower input region when the ACh/NE modulation increased (Fig 8C, upper light blue area).

Under the condition of high ACh/NE modulation (100%), the thalamic network generated θ oscillations (6.7 Hz) without any input (0 nS, Fig 8F1) due to spontaneous HTBs of HTC cells. The bursting frequency increased to α band (8–14 Hz) when the afferent input slightly increased (e.g., 1.5 nS, Fig 8F2; similar to Fig 2C1). With further increase of the afferent input (e.g., 10 nS), HTC cells switched from HTBs to tonic spiking and the network synchronized at the β frequency band (23.8 Hz; Fig 8F3). With strong afferent excitation (e.g., 15 nS), the oscillation frequency of the thalamic network increased to the γ band (31.7 Hz, Fig 8F4; similar to Fig 2D1), which gave rise to potential γ oscillations. Note that INs had no spiking activities for medium or large afferent inputs (Fig 8F3 and 8F4, *upper middle*), different from default γ oscillations (Fig 2D1, *upper middle*). This was because INs did not receive direct afferent inputs in producing the oscillation map, while INs received weak afferent inputs (1.5 nS, Table 1) in the default γ simulation. In addition, different from the desynchronizing state under the low and medium ACh/NE conditions (S15 Fig and Fig 8E4), strong afferent drive did not disrupt network synchrony in the high ACh/NE condition (>80%; Fig 8F3 and 8F4). This was because HTC cells were much more excitable with high ACh/NE modulation and fired rhythmic spiking activities that were synchronized by gap junctions even when the high-threshold T-type Ca^2+^ current was inactivated. Thus, the model suggests that fast β/γ oscillations only occur at high ACh/NE level in the thalamic network.

### Responses of the thalamic network to periodic stimulation

**Fig 9:**
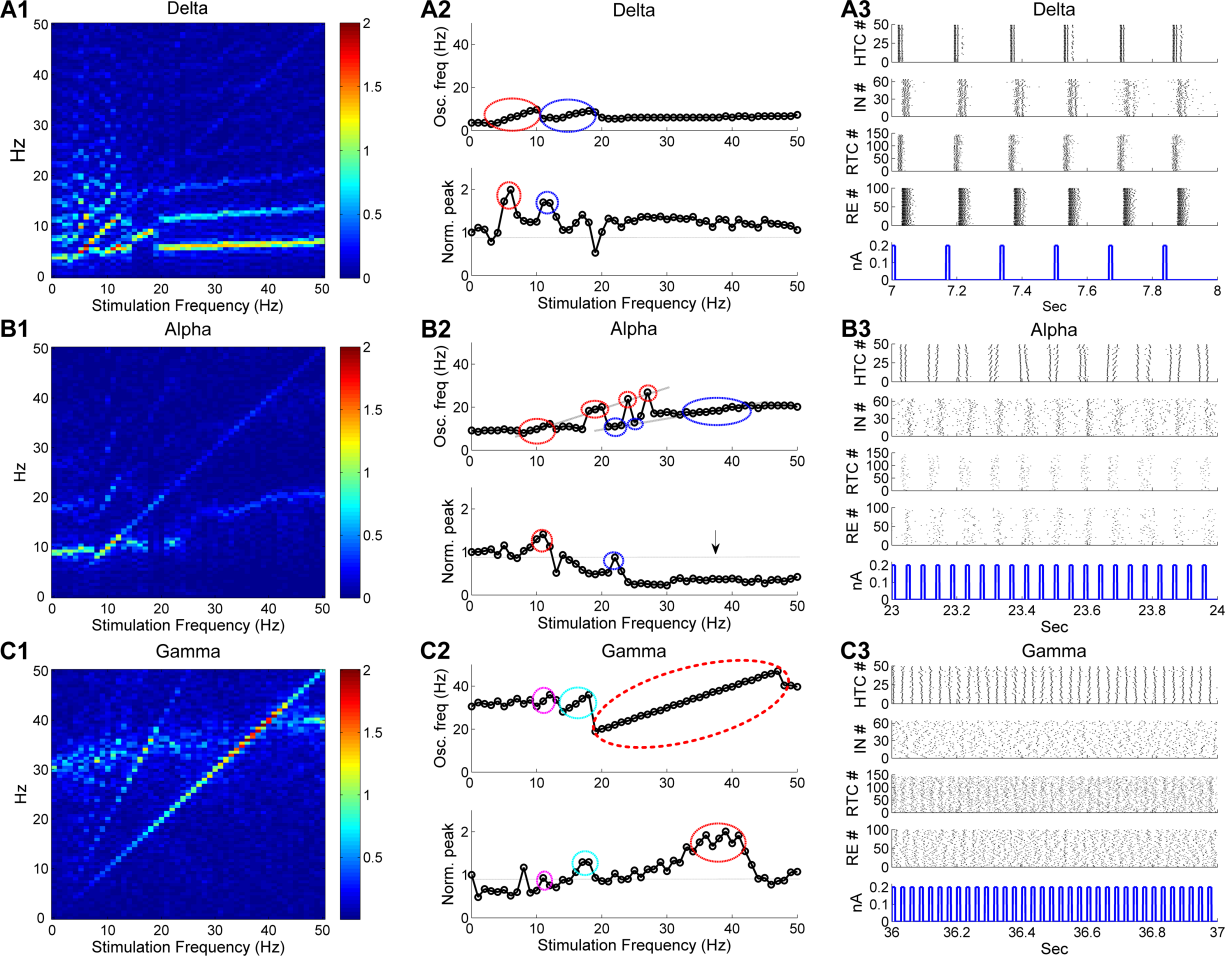
Stimulation of LGN induces state-dependent entrainment and resonance in three oscillatory states. (A) Thalamic network dynamics during stimulation of δ oscillations. **(A1)** Normalized color-coded frequency power spectrum of sLFP. **(A2)** Dominant network oscillation frequency (*top*) with normalized spectral peak (*bottom*) as a function of the stimulation frequency.**(A3)** Sample of thalamic network activity during stimulation. Stimulation amplitude is 0.2 nA. The bottom panel in **(A3**) displays the stimulation pulses and the stimulation frequency is 6 Hz. (B) Thalamic network dynamics during stimulation of α oscillations. Stimulation frequency is 22 Hz for **(B3).** The vertical arrow in (**B2**) indicates oscillation suppression. (C) Thalamic network dynamics during stimulation of γ oscillations. Stimulation frequency is 35 Hz for **(C3)**. Red ellipses in **(A2-C2)** indicate primary entrainment (1:1) and resonance, blue ellipses indicate the first subharmonic entrainment (2:1) and first harmonic resonance, cyan ellipses indicate the first harmonic entrainment (1:2) and first subharmonic resonance; magenta ellipses indicate the second harmonic entrainment (1:3) and second subharmonic resonance. The dotted horizontal lines in **(A2-C2)** indicate the normalized spectral peak without stimulation.

Lastly, we applied periodic stimuli to the thalamic network to examine how distinct thalamic oscillations are modulated by rhythmic perturbations. The phasic pulsatile stimuli were introduced to the LGN and we assumed that all TC cells and IN neurons received the same stimulation input. To analyze the impact of stimulation on thalamic oscillation dynamics, we plotted the normalized color-coded frequency power spectrum of the sLFP (frequency spectrum heat map) in response to ascending stimulation (1–50 Hz) with a fixed stimulation amplitude (0.2 nA) for three major oscillatory states (δ, α and γ oscillations, Fig 9A1–9C1). In addition, to examine how the dominant oscillatory dynamics varied with the stimulation frequency, we plotted the dominant network oscillation frequency with normalized spectral peak as a function of the stimulation frequency for all three oscillatory states in Fig 9A2–9C2. Consistent with a recent computational study of an abstract cortical model [44], stimulation induced entrainment and resonance in the thalamic network model in all three oscillatory states. Importantly, we found that the occurrence of these phenomena was state-dependent. Entrainment, a response pattern where the intrinsic oscillations are locked to the simulation, was reflected by the highlighted spectral power along the diagonal with multiple harmonic and/or subharmonic components (above and below the diagonal) in the frequency spectrum heat map (Fig 9A1–9C1). Entrainment was much more prominent during stimulation of γ oscillations than δ and α oscillations indicated by the longer and higher diagonal power during γ oscillations (compare Fig 9C1 with Fig 9A1 and 9B1). The state-dependent entrainment effect was also evident in the dominant frequency plot where the entrained frequency range during primary entrainment (1:1 entrainment) was much wider during stimulation of γ oscillations than δ and α oscillations (compare Fig 9C2 with Fig 9A2 and 9B2, *top*; enclosed by red ellipses). In addition, we observed discontinuous entrainment where the thalamic network switched between the entrained and unentrained states during stimulation of α oscillations, but not during the other two oscillatory states (compare Fig 9B2 with Fig 9A2 and 9C2, *top*; enclosed by red ellipses). The entrainment behavior of the thalamic network during δ oscillations is illustrated in Fig 9A3. In response to 6 Hz stimulation, all four types of thalamic neurons fired highly synchronized bursts at the same stimulation frequency (6 Hz) that were tightly phase-locked to the stimulation pulses. We also note that primary entrainment occurred when the stimulation frequency was close to but mostly higher than the endogenous frequency for all three oscillatory states (Fig 9A2–9C2, *top*; enclosed by red ellipses), suggesting that stimulation favored higher frequency entrainment. For example, primary entrainment occurred at 3–10 Hz during stimulation of δ oscillations (endogenous frequency: 3.7 Hz) and took place at 19–47 Hz during stimulation of γ oscillations (endogenous frequency: 30.5 Hz). Besides primary entrainment, thalamic network oscillations were also shaped by subharmonic as well as harmonic entrainment (indicated by blue, cyan and magenta ellipses, Fig 9A2–9C2, *top*). The thalamic network activity during subharmonic entrainment of α oscillations is illustrated in Fig 9B3. In response to 22 Hz stimulation, HTC cells burst at half of the stimulation frequency (11 Hz) and the whole network synchronized at 11 Hz. By comparison, during (the first) harmonic entrainment of γ oscillations, HTC cells oscillated at twice the stimulation frequency (S17 Fig).

Stimulation of the LGN also induced resonance, an enhancement of oscillation power when the stimulation frequency was close to the endogenous frequency, its harmonics and/or subharmonics. The thalamic neuronal activity during primary resonance is exemplified in Fig 9C3. When the LGN was stimulated at 35 Hz, close to the endogenous frequency (30.5 Hz), the spiking activities of all four types of neurons became more synchronized and rhythmic than without stimulation (compare Fig 9C3 with Fig 2D2). Resonance occurred in all three oscillatory states (Fig 9A2–9C2, *bottom*), but with substantial differences among the states. During stimulation of γ oscillations, the primary resonance peak was much wider than that during stimulation of δ or α oscillations (compare Fig 9C2 with Fig 9A2 and 9B2, *bottom*; enclosed by red ellipses), in line with much wider primary entrained frequency range during stimulation of γ oscillations. Notably, similar to entrainment, resonance was asymmetric with respect to the endogenous frequency and favored higher frequency stimulation. For instance, the primary resonance peak occurred at 5 and 6 Hz during stimulation of δ oscillations (Fig 9A2, *bottom*; enclosed by red ellipse) and 10 and 11 Hz during stimulation of α oscillations (Fig 9B2, *bottom*; enclosed by red ellipse), both of which were higher than their respective endogenous frequencies (3.7 Hz and 9.2 Hz respectively). Such asymmetry was most prominent during stimulation of γ oscillations. Although the endogenous γ frequency was 30.5 Hz, stimulation induced maximal resonance (> 60%) between 35 and 41 Hz (Fig 9C2, *bottom*; enclosed by red ellipse). The strong bias towards higher frequency in both entrainment and resonance suggests that stimulation increases the endogenous frequency of thalamic oscillations by enhancing the excitability of TC neurons. In addition to resonance enhancement, we observed substantial power suppression during high frequency (> 25 Hz) stimulation of α oscillations (Fig 9B2, *bottom*; indicated by black arrow), but not during the other two oscillatory states. This was because high frequency stimulation switched HTC bursting to tonic spiking and suppressed RTC activities by increasing IN firing (S18 Fig). Overall, stimulation of the thalamic network induces state-dependent entrainment and resonance, which are stronger during γ oscillations than δ and α oscillations.

## Discussion

Given the dominant focus on cortical circuits for the mechanistic study of brain oscillations, the thalamus has been somewhat overlooked, despite repeated suggestions that the thalamus may serve as a “pacemaker” of brain oscillations [7, 8, 45, 46]. Indeed, recent experimental findings indicate that the thalamus plays a key role in controlling cortical states and functioning [32, 47, 48] and regulating the information transmission between different cortical areas [49, 50]. However, it is not clear whether the thalamus can independently generate different types of brain oscillations without interaction with the cortex. Answering this question will help to determine the validity of a thalamic pacemaker model. Using a biophysically realistic thalamic network model, we provided for the first time a full account of how multiple distinct oscillations could arise from the cellular and network properties of the thalamic circuitry and how thalamic oscillation transitions from one state to the another. This model then enabled us to investigate the state-dependent response to brain stimulation.

### State-dependent oscillatory properties of TC cells underlie the genesis of distinct thalamic oscillations

**Fig 10:**
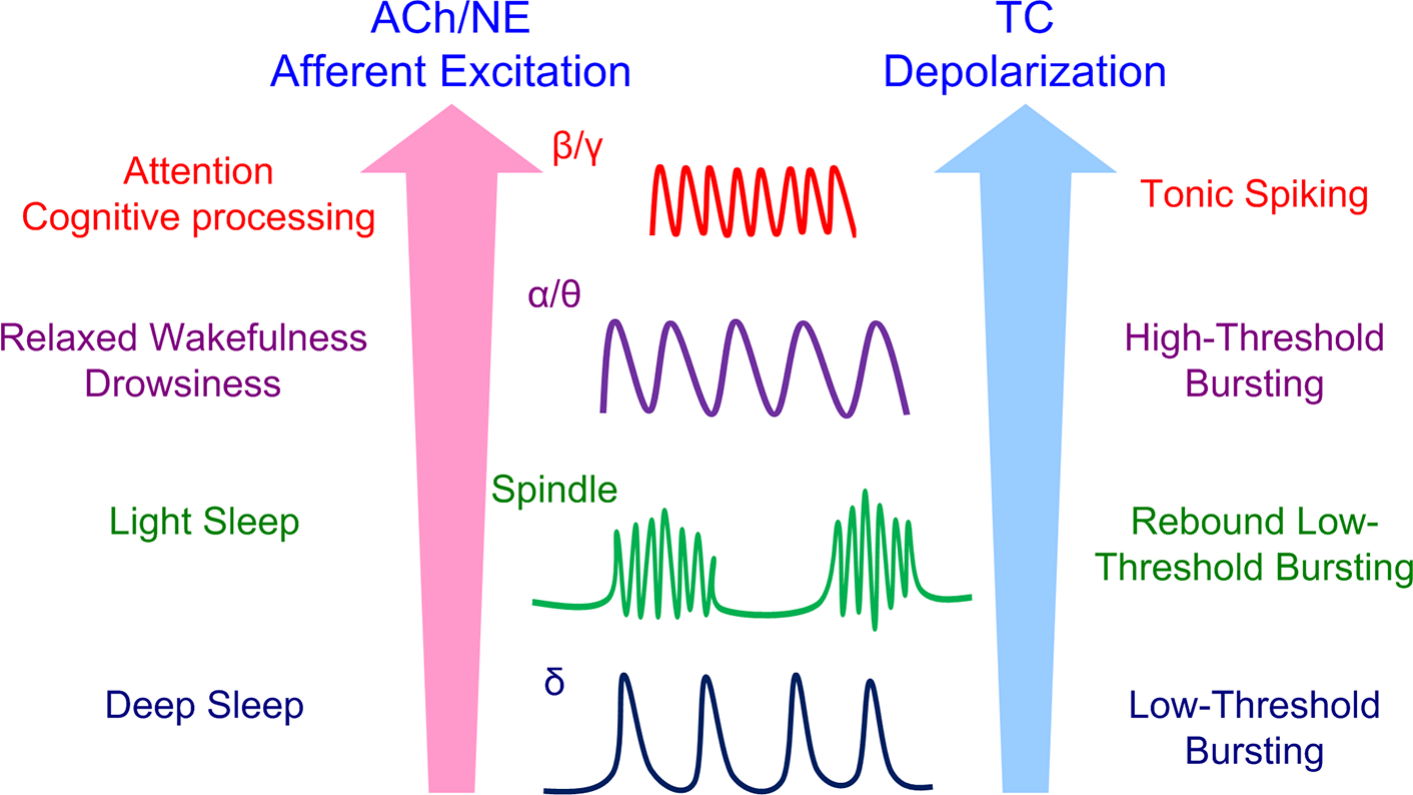
A unified hypothesis for thalamic oscillatory state generation and transition. During deep sleep, both the ACh/NE modulation and afferent excitation are very low. TC cells generate intrinsic slow low-threshold bursts (LTBs) that underlie δ oscillations. During light sleep with increased ACh/NE modulation and afferent excitation, TC cells are not spontaneously bursting but can produce rebound LTBs that mediate spindle oscillations. During relaxed wakefulness or drowsiness, the ACh/NE modulation is significantly increased and HTC cells fire spontaneous high-threshold bursts (HTBs) which underlie both α and θ oscillations depending on afferent excitation. During attention or cognitive processing, TC cells are further depolarized to fire high-frequency tonic spiking that is synchronized by gap junctions and feedback inhibition to generate fast β or γ oscillations.

The key for the genesis of multiple distinct oscillations is that TC cells exhibit multiple oscillatory bursting/spiking patterns depending on neuromodulation and afferent excitation level (S1 Fig). In the low ACh/NE modulation state, TC cells are sufficiently hyperpolarized to generate low-threshold bursts (LTBs) in the δ frequency band mediated by the low-threshold T-type Ca^2+^ current [51, 52]. Such low-frequency LTBs form the neuronal basis of δ oscillations (Fig 10). In the medium ACh/NE modulation state, although TC cells are not spontaneously bursting, hyperpolarization of TC cells by afferent input or inhibition evoked rebound LTBs after the release of hyperpolarization [53, 54]. Such hyperpolarization-induced rebound LTBs are necessary for spindle oscillations (Fig 10). In the high ACh/NE modulation state, HTC cells fire high-threshold bursts (HTBs) in the θ/α frequency band that mediate θ or α oscillations depending on afferent excitation level (Fig 10; [7, 12]). Stronger depolarization of HTC cells by afferent input switches HTBs into high frequency tonic spiking enabling γ oscillations (Fig 10). Such high frequency tonic spiking of TC cells was supported by experimental data that TC neurons exhibited fast oscillatory discharge in the γ frequency band in LGN (~50 Hz; [11]) and in the ventroanterior-ventrolateral (VA-VL) complex (20–40 Hz; [27]). Thus, the rhythmic burst/spiking properties of TC neurons provide the cellular mechanism underlying multiple distinct thalamic oscillations [55].

### Thalamic network is endowed with multiple synchronizing mechanisms

To generate oscillations at the population level, the rhythmic burst firing or tonic spiking of TC neurons has to be synchronized. We found that the specific connectivity of the thalamic network endows it with the capability to synchronize under different neuromodulatory states and over a wide range of frequencies. First, the existence of gap junctions plays a crucial role in the synchronization of network activity. In particular, the gap junctions between HTC cells [7, 12, 17, 18] enable them to serve as a “pacemaker” or synchronization “engine” of the circuit that remain synchronized even in the presence of strong random noisy inputs. We showed that thalamic oscillations were either impaired or eliminated if the HTC gap junctions were removed, consistent with experimental observation [12, 35]. Besides HTC gap junctions, the weaker gap junctions between HTC and RTC cells and the inter-RE gap junctions also contribute to the synchronization of the network. Second, the feedforward IN inhibition and feedback RE inhibition lead to synchronization of RTC cells. Different from HTC cells, there are no gap junctions among RTC neurons [7, 17]. Nevertheless, synchronized HTC activity propagates to both IN and RE neurons, which constrain RTC firing via feedforward inhibition. As RTC cells also project excitatory inputs on RE neurons, they receive feedback RE inhibition as well which enhances the synchronization. During low frequency oscillations (δ, spindle and α), we observed that either IN or RE inhibition was sufficient to synchronize RTC activity, while during fast frequency oscillations (β/γ), simultaneous rhythmic IN and RE inhibition was needed for high level of RTC synchrony due to strong random noisy inputs (S9 Fig). Thus, the presence of both IN and RE inhibition strengthens the synchronization of TC neurons. Third, multiple mechanisms contribute to the generation, synchronization and stability of thalamic oscillations. The specific connectivity of the thalamic circuit allows for the synergy of multiple mechanisms in the generation of synchronized oscillations. For example, δ oscillations are synchronized by both gap junctions and inhibition (Fig 4). During spindle oscillations, both the feedforward IN and feedback RE inhibition contribute to the rebound bursting of TC cells. In particular, the feedforward HTC→IN→RTC inhibition could also lead to feedback inhibition on HTC cells via the HTC-RTC gap junctions. Similarly, during fast β/γ oscillations, while the HTC gap junctions play a major role, the feedback RE inhibition could also contribute to fast oscillations if the inhibitory strength is sufficiently strong. We hypothesize that the existence of multiple synchronizing mechanisms coupled with the strong intrinsic oscillatory properties of TC cells enables the thalamic network to serve as a pacemaker during thalamocortical oscillations, consistent with experimental observation [56].

### ACh/NE modulation and afferent excitation set the thalamic oscillation state

To further test the hypothesis that generation and transition of distinct thalamic oscillations are functions of both ACh/NE neuromodulation and afferent excitation, we varied the ACh/NE and input levels systematically to identify the prominent oscillatory regimes across the entire parameter space. The oscillation transition map (Fig 8C) confirmed the existence of multiple distinct oscillations under physiological conditions (Fig 2B): δ oscillations with low ACh/NE modulation and minimal input (deep sleep); spindle oscillations with medium ACh/NE modulation and slight to weak inputs (light sleep); α/θ oscillations with high ACh/NE modulation and weak input (relaxed wakefulness) and γ/β oscillations with high ACh/NE modulation and strong input (arousal and attention). One interesting and surprising finding from the oscillation transition map is that it reveals prominent α oscillations under low ACh/NE modulation and with large afferent input thus extending the original model hypothesis where α oscillations occur under high ACh/NE modulation and with low afferent input (compare Fig 8C with Fig 1B). Note that α oscillations arising from these two different regions have different physiological implications. While α oscillations under high ACh/NE modulation correspond to the state of relaxed wakefulness, α oscillations under low ACh/NE modulation represent a non-physiological state in deep sleep where the thalamus receives strong afferent drive.

In addition, the oscillation transition map reveals several important insights and predictions as to the distribution and transition of thalamic oscillations. First, there exist two separate regions for the generation of θ oscillations. The first region locates between δ and spindle oscillations and is mediated by the LTBs of TC cells, while the second region resides between spindle and α oscillations and is mediated by HTBs of HTC cells. Thus, the model predicts the existence of θ oscillations during the transition from spindle to δ oscillations. Also, persistent and coherent θ oscillations mediated by LTBs are definitive signatures of a number of neurological or psychiatric disorders including Parkinson’s disease, major depressive disorder (MDD) and schizophrenia [6, 7]. Hence, the θ oscillation region mediated by LTBs could potentially correspond to a pathological state. Second, spontaneous spindle oscillations can be generated under relatively low ACh/NE modulation but with moderate level afferent input. Spindle oscillations can be initiated by a number of mechanisms including spontaneous firing from a more excitable region of the thalamus, spontaneous oscillating TC cells or cortical stimulation through excitation of RE cells [57]. In the default simulation (Fig 2B), we used a brief input to RE cells to induce spindle oscillations. Here we showed that spindle oscillations could also be initiated by spontaneous firing of TC cells given that the random background inputs are relatively high, consistent with previous hypotheses [57]. Third, there exist transient θ/α oscillations under medium ACh/NE modulation and with moderate level of afferent input. During the transition from spindle oscillations to persistent θ oscillations under medium level ACh/NE (e.g., 50%), we observed transient θ/α oscillations which lasted for a few hundred milliseconds (e.g., Fig 8E2). Such transient θ/α oscillations were induced by a surge of background inputs, sustained a few cycles through the TC-RE interaction and terminated because of the inactivation of the low-threshold Ca^2+^ current (due to afferent input; returning back the resting state), similar to spontaneous spindle oscillations. Interestingly, recent experimental studies discovered transient or intermittent α/β events in the awake mammalian neocortex [58, 59]. Combined experimental and computational evidence showed that such transient oscillations emerged from the integration of synchronous bursts of excitatory synaptic drive targeting proximal and distal dendrites of pyramidal neurons [59]. Thus, though both the thalamus and neocortex are capable of generating transient rhythms, their underlying mechanisms may be different. Fourth, the effect of afferent input is somewhat equivalent to ACh/NE modulation. For example, without afferent input (0 nS), as the ACh/NE modulation increased, the thalamic oscillatory state switched from δ oscillations to θ oscillations and to spindle oscillations and to θ oscillations again (Fig 8C). Similarly, for 0% ACh/NE, as the afferent input increased, the thalamic oscillation switched from δ oscillations to θ oscillations and to spindle oscillations after a brief quiescent state followed by θ oscillations again (Fig 8C). Lastly, fast frequency β or γ oscillations in the thalamus can only be generated under high ACh/NE modulation. This is because under low and medium ACh/NE modulation, large (random) afferent input desynchronizes the network when the HTBs of HTC cells switch to sparse tonic spiking. This is consistent with the experimental data that fast γ oscillations in the thalamus are supported by cholinergic projection from the brainstem [27] and acetylcholine release contributes to gamma oscillations in prefrontal cortex during attention [60].

### The role of cortex in thalamic oscillations

We used a brief depolarizing input (to RE neurons) to trigger spindle oscillations in the default simulation (Fig 2B). Such brief depolarization could arise from the cortex; for example, the initial portion of the cortical up state during slow oscillations (<1 Hz) triggers thalamic spindles [61, 62] and cortical stimulation induces spindle oscillations in the thalamus [29]. Alternatively, such transient depolarization may result from a synchronized surge of random background inputs to TC or RE neurons, as discussed above. In either case, spindle oscillations are generated internally from the thalamic circuit in the absence of cortical modulation, as observed experimentally [63, 64]. Besides, substantial depolarization of TC cells is required to generate γ rhythms in the thalamic network [27, 37]. Such strong depolarization could result from a combination of sensory stimulus [11], elevated cholinergic neuromodulation [27] and increased corticothalamic projection to the thalamus [24]. Independent of the source of depolarization, it should be emphasized that we modeled the TC depolarization (in the high ACh/NE modulation state) by increasing the synaptic strength of the random Poisson inputs to TC cells which did not contain any rhythmic structure. Hence, although the cortex may provide necessary inputs to trigger spindles and depolarize TC neurons during γ oscillations, it is through the intra-thalamic mechanisms that the thalamic model produces distinct states of oscillatory patterns, in agreement with experimental findings [2, 24, 27].

Our modeling results suggest that the thalamus could be a driving force for thalamocortical oscillations, consistent with experimental observations [7, 8, 46]. Nevertheless, this prediction does not preclude the existence of cortically-originated oscillations in the thalamocortical systems. Indeed, δ oscillations are found to contain both thalamic and cortical components [37] and the cortical δ waves persist in the absence of the thalamus [65]. Similarly, α oscillations could be of cortical origin mediated by layer 5 pyramidal cells [10]. Also, corticothalamic feedback strongly modulates spindle oscillations [33] and synchronizes spindle waves to widespread cortical areas [63]. Moreover, γ oscillations in both the visual and motor cortex could arise from intracortical mechanisms [9, 66]. Thus, it is possible that independent neural generators exist for distinct thalamic and cortical oscillations and the corticothalamic feedback synchronizes the thalamic and cortical rhythms into coherent thalamocortical oscillations [3, 37, 67]. Indeed, it has been proposed that three cardinal oscillators (one cortical oscillator and two thalamic oscillators) underlie the generation of the slow (< 1 Hz) electroencephalogram (EEG) rhythm of NREM sleep through intricate dynamic interactions [68]. Future studies are needed to examine how independent cortical and thalamic oscillators interact dynamically to create coherent rhythms in the thalamocortical system.

### State-dependent entrainment of thalamic oscillations by stimulation

Rhythmic stimulation has become an important and promising technique to study the causal role of oscillations in brain function [69, 70] and as therapeutic intervention to treat neurological and psychiatric disorders [71, 72]. As such, the responses of neuronal circuits to periodic perturbation have been examined in a number of experimental [73, 74] and computational/theoretical studies [44, 75–79]. Most of the existing theoretical models focused on the nonlinear dynamics of neurons using simplified neuronal models (e.g., integrate-and-fire neurons [75–77]) and did not consider the interaction between rhythmic input and intrinsic neuronal dynamics. One notable exception was a cortical network model developed by Tiesinga [78] that investigated the dependence of LFP resonance on different biophysical time scales of the neuronal circuit. Since the Tiesinga model focused on pyramidal interneuron network gamma (PING) in the cortex, it remains unclear how the stimulation interacts with endogenous neural dynamics and depends on the physiological state of the thalamus. The manifestation of multiple distinct oscillations in one unified biophysical thalamic model enabled us to examine the impact of rhythmic stimulation on thalamic network dynamics. When subject to periodic stimulation, the thalamic network displayed two most prominent response patterns, entrainment and resonance, as shown previously in more abstract neuronal models [44, 75–77]. Importantly, such response patterns were highly state dependent in that stimulation of γ oscillations induced much stronger entrainment and resonance than δ and α oscillations (Fig 9). We hypothesize that this is because γ oscillations are mainly driven by afferent excitation and the network synchrony or endogenous oscillation power is much lower compared with δ or α oscillations which are driven by intrinsic mechanisms (i.e., neuronal bursting; Fig 2). Thus, during fast γ oscillations, the thalamus can more effectively relay sensory inputs than slow δ and α oscillations (i.e., easier to be entrained). Our simulation results are consistent with experimental observations that reduced α oscillation power enables entrainment [80] and fast γ band oscillations facilitate visual information processing [73, 81]. In addition to entrainment and resonance, high frequency stimulation induced strong suppression on α oscillations, which was not observed during stimulation of δ and γ oscillations for the same stimulation amplitude (Fig 9). Taken together, our model is the first to demonstrate how the response patterns of the thalamic network to periodic stimulation depend on the physiological state or intrinsic dynamics of constituent neurons. As the particular oscillation state of the thalamus is set by both ACh/NE modulation and afferent excitation, our model thus suggests that ACh/NE and afferent excitation also define the thalamic response to brain stimulation.

### Previous models of thalamic oscillations

Thalamic oscillations have been modeled by a number of previous studies, either in isolated thalamus [57, 82–84] or integrated thalamocortical network [15, 85–93]. Several novel features distinguish our model from the existing thalamic models. First, our model incorporated a newly identified, special subclass of TC cells, high-threshold bursting TC cells (HTCs) and the gap junctions among HTCs [12, 17, 18]. In addition, the model contained gap junctions between HTCs and relay-mode TC cells (RTCs) [17] and gap junctions among reticular neurons [94, 95], which enhanced the thalamic network synchrony. The only existing model that consisted of HTCs and gap junctions was developed by Vijayan and Kopell [84] to study α oscillation and its role in stimulus processing. However, the Vijayan and Kopell model did not model INs explicitly and did not include the gap junctions between HTCs and RTCs and among RE cells. Also, the Vijayan and Kopell model did not consider the action of NE on α oscillations. As a result, the RE neurons were silent due to ACh inhibition during the muscarinic ACh receptor- induced α activity (Fig 1B of [84]). By comparison, our model considered the combined action of ACh and NE so RE cells fired tonic spiking during α oscillations (Fig 2C).

Second, our thalamic model integrates multiple distinct oscillations (δ, spindle, α/θ, and γ/β) into one unified framework, in a similar spirit to a previous model of carbachol-induced δ, θ and γ oscillations in the hippocampus [96]. Notably, all four neuronal models (HTC, RTC, IN and RE) in the thalamic network were parameterized carefully to produce experimentally observed firing patterns under different neuromodulatory states (S1 Text, S1 Fig and S2 Fig), enabling it to generate distinct, neuromodulation-dependent oscillation patterns. In contrast, most of the existing thalamic models focused on only one or two specific oscillatory patterns such as α oscillations [84], spindle oscillations [15, 57, 83, 89, 90], δ and spindle transition [82, 85], spindle and gamma oscillations [88] or spindle and slow (< 1 Hz) oscillations [92]. Other thalamocortical models have studied the transition from slow sleep oscillations (< 1 Hz) to asynchronous waking state [86, 87]. It should be noted that a recent thalamocortical model from the Bazhenov group also investigated the generation and transition of multiple distinct oscillations during the sleep stages: spindle in NREM 2, slow δ oscillations during NREM 3 and α or mu-like rhythm during REM sleep [93]. There are several differences between our model and the Krishan et al. model. First, the Krishan et al. model focused δ band activity mostly on slow oscillations (0.5–1 Hz) generated from the cortex, while our model considered regular δ oscillations (1–4 Hz) originated from thalamic TC cells. Second, the α or mu-like rhythm in the Krishan et al. model was generated by sparse synchronized single spiking in the cortex, while the α oscillation in our model was produced by synchronized high-threshold bursting in thalamic HTC cells. Third, our thalamic model was able to produce fast frequency γ/β oscillations, while the Krishan et al. model did not consider fast frequency oscillations. Last, we only varied two model parameters (potassium leak conductance and synaptic input conductance) to generate multiple oscillations, while more parameter change was needed to switch oscillation from one state to the other in the Krishan et al. model.

Lastly, our model is the first to consider the co-regulation of neuromodulation and afferent input in thalamic oscillatory state transition. The effects of neuromodulation on thalamic oscillatory state transition have been examined in a number of models (e.g., [87, 92, 93]), but these previous models mostly focused on the sole action of neuromodulation. By comparison, we investigated the combined effect of neuromodulation and afferent input and demonstrated that the generation and transition of thalamic oscillations are functions of both neuromodulation and afferent excitation.

### Model limitations

As for any scientific study, there are several limitations to our work. First, as mentioned above, in addition to acetylcholine and norepinephrine, thalamic processing is subject to other neuromodulator action such as histamine (HA), serotonin (5-HT) and adenosine [25]. Similar to ACh and NE, most of these neuromodulators target the potassium leak current (*I*_KL_) in TC and RE neurons [25]. For example, application of HA leads to a slow depolarization in TC cells by decreasing the *I*_KL_ [97]. Also, application of 5-HT *in vitro* strongly depolarize RE neurons via reduction of the *I*_KL_, an effect similar to noradrenergic modulation [98]. Thus, the net effect of these neuromodulators seems to be a depolarization of both TC and RE neurons and we hypothesize that activation of multiple neuromodulatory systems strengthens the neuromodulatory control of thalamic oscillatory state transition. Second, cholinergic and noradrenergic inputs modulate other ionic currents in thalamic cells other than *I*_KL_. For example, the hyperpolarization-activated cation current *I*_H_ in TC cells is modulated by NE [99] and variation of *I*_H_ conductance density was able to switch the oscillatory pattern between δ and spindle-like oscillations in a TC cell model [82]. Also, ACh may influence the muscarinic current *I*_M_ and affect excitatory synaptic strength in the thalamus, similar to its modulatory effect in the cortex [100]. We only considered the modulation of *I*_KL_ since it is the major target of cholinergic and noradrenergic inputs [25, 26, 101] and we attempt to model the transition of multiple oscillatory states with minimal change of parameters. Thus, our model can be considered as a minimal model of thalamic oscillation transition and inclusion of other ionic currents modulated by ACh/NE can be studied in future modeling studies. Third, in our model, δ oscillations occur during deep sleep stage, which corresponds to low ACh/NE modulation and minimal afferent excitation (Fig 8C), consistent with experimental observation [29, 31]. A recent experimental work in nonhuman primates demonstrates that in primary auditory cortex δ and γ oscillations co-occur during attentive processing while α and β oscillations occur during periods of inattention [102]. The existence of δ oscillations in the study could be due to the fact that both the auditory and visual stimuli are presented in the δ frequency band (1.6 Hz and 1.8 Hz respectively). In addition, our modeling results agree with the experimental data [102] that α oscillations occur during inattention while γ oscillations occur during attention. Future study is needed to examine δ and γ phase coupling during attention in a thalamocortical model subject to rhythmic δ band stimulation. Lastly, as the major goal of this study is to determine whether an isolated thalamus is capable of generating multiple distinct oscillation patterns, it presently does not include the cortex. The absence of the cortex prevents us modeling certain oscillatory pattern that is of cortical origin such as slow oscillations (< 1 Hz; [86, 87, 103]). Nevertheless, a deeper mechanistic understanding of thalamic oscillations enables the systematic investigation of the cellular and circuit mechanisms of thalamocortical rhythms in the future.

## Methods

### Network structure

The thalamic network consisted of both the lateral geniculate nucleus (LGN) and the reticular nucleus (TRN) (Fig 1A). The LGN contained two major cell types: thalamocortical (TC) cells and local interneurons (INs), and the TRN contained reticular (RE) cells. The TC cells were further divided into high-threshold bursting TC (HTC) cells and relay-mode TC (RTC) cells, based on whether TC cells can generate high-threshold bursting or not [12, 18]. In the cat LGN, HTC cells account for about 25%-30% of the whole TC population [12, 17], while INs constitute about 25% of the total neuronal population in all dorsal thalamic nuclei of cats and primates [104]. Accordingly, the thalamic network contained 49 (7×7) HTC cells, 144 (12×12) RTC cells, 64 (8×8) INs and 100 (10×10) RE neurons, all placed in a two-dimensional grid. The major modeling results were robust to the network size when the synaptic weight and connectivity density were scaled accordingly. The network connectivity between the four types of neurons was illustrated in Fig 1A. HTC cells were connected with gap junctions [12, 17] and provided feedforward excitation to INs, which in turn delivered feedforward inhibition to RTC cells [18]. A small percentage (20%) of RTC cells were also connected with HTC cells via gap junctions [17]. Both HTC and RTC cells sent glutamatergic synapses to RE neurons, while receiving GABAergic feedback inhibition from the RE population [20, 105]. RE neurons were connected with both gap junctions [94, 95] and GABAergic synapses [106, 107]. Lastly, a small percentage (~10%) of RE neurons project GABAergic synapses to local interneurons [108].

In the model, all gap junction connections were local and the maximal distance between two electrically coupled neurons was two units (the distance between two adjacent neurons in horizontal or vertical direction was assumed to be one unit). Each HTC cell formed gap junctions with neighboring HTC cells with a random connection probability. Also, 20% of RTC cells (randomly selected) formed gap junctions with neighboring HTC cells and 20% of RE cells (randomly selected) formed gap junctions with neighboring RE cells. For all gap junction connections, the connection probability was taken to be 30% within the local region. By comparison, all chemical synapses were global in this relatively small network. The connection probability (0.3) was higher for the TC-IN connections (including HTC→IN and IN→RTC projections) than that (0.2) for the TC-RE connections (including HTC→RE, RTC→RE, RE→HTC and RE→RTC projections) because TC cells show higher correlation with INs than with RE cells during α oscillations in cats [18]. A connection probability of 0.2 was used for the RE→RE synapses, while a much smaller probability (0.05) was used for the RE→IN synapses according to experimental data [108].

### Single-cell models

Following previous “point” models of thalamic cells [57, 82–84, 86, 109], all single cell models in the thalamic network contained one single compartment and multiple ionic currents described by the Hodgkin-Huxley formulism [110]. The current balance equation was given by:

$$C_{m}\frac{dV}{dt}=-g_{L}(V-E_{L})-g_{KL}(V-E_{KL})-\sum I^{int}-10^{-3}\sum \frac{I^{syn}}{A}+10^{-3}\frac{I_{app}}{A}$$ (1)

where *C*_*m*_ = 1μF/cm^2^ is the membrane capacitance for all four types of neurons, *g*_*L*_ is the leakage conductance (nominal value: *g*_*L*_ = 0.01 mS/cm^2^ for all four types of cells) and *g*_*KL*_ is the potassium leak conductance modulated by both ACh and NE (see Table 1 and below for details). *E*_L_ is the leakage reversal potential (*E*_*L*_ = −70 mV for both HTC and RTC cells; *E*_*L*_ = −60 mV for both IN and RE neurons), and *E*_KL_ is the reversal potential for the potassium leak current (*E*_*KL*_ = −90 mV for all neurons). *I*^int^ and *I*^syn^ are the intrinsic ionic currents (in μA/cm^2^) and synaptic currents (in nA) respectively and *I*_app_ is the externally applied current injection (in nA). The following total membrane area (*A*) was used to normalize the synaptic and externally applied currents in Eq (1): HTC and RTC cells: 2.9×10^−4^ cm^2^ [109]; INs: 1.7×10^−4^ cm^2^ [111]; RE cell: 1.43×10^−4^ cm^2^ [57, 86].

#### Thalamocortical cells

Both HTC and RTC cells contained the following six active ionic currents: a spike generating fast sodium current (*I*_Na_), a delayed rectifier potassium current (*I*_DR_), a hyperpolarization-activated cation current (*I*_H_), a high-threshold L-type Ca^2+^ current (*I*_Ca/L_), a Ca^2+^- dependent potassium current (*I*_AHP_) and a Ca^2+^- activated nonselective cation current (*I*_CAN_). In addition, both TC cells included a regular low-threshold T-type Ca^2+^ current (*I*_Ca/T_) and a high-threshold T-type Ca^2+^ current (*I*_Ca/HT_); the conductance density of *I*_Ca/HT_ was much higher in HTC cells than in RTC cells to support high-threshold bursting (S1 Table; [12]). The kinetics of *I*_Na_ and *I*_DR_ were taken from a previous TC cell model [86] with origin from [112], whereas the kinetics of *I*_Ca/T_ and *I*_H_ were taken from a previous computational study of rhythmic oscillation in thalamic relay neurons [113]. To obtain the high-threshold T-type current *I*_Ca/HT_, both the activation and inactivation of the *I*_Ca/T_ current was shifted by 28 mV, similar to a previous TC modeling study [84]. The mathematical description of *I*_Ca/L_ and *I*_CAN_ was adapted from [114], and that of *I*_AHP_ was based on [84].

#### Interneurons

Based on previous modeling study [111], the IN model cell contained the following six active ionic currents: a spike generating fast sodium current (*I*_Na_), a delayed rectifier potassium current (*I*_DR_), a hyperpolarization-activated cation current (*I*_H_), a high-threshold T-type Ca^2+^ current (*I*_Ca/HT_), a Ca^2+^- dependent potassium current (*I*_AHP_) and a Ca^2+^- activated nonselective cation current (*I*_CAN_). All the currents were modeled the same as those for the TC cells.

#### Reticular cells

According to experimental data [115, 116], the RE model cell included five active ionic currents: a spike generating fast sodium current (*I*_Na_), a delayed rectifier potassium current (*I*_DR_), a low-threshold T-type Ca^2+^ current (*I*_Ca/T_), a Ca^2+^-dependent potassium current (*I*_AHP_) and a Ca^2+^- activated nonselective cation current (*I*_CAN_). The kinetics of *I*_Ca/T_ was taken from [117]. Other currents were modeled the same as those for the TC cells.

### Current kinetics

All active ionic conductances were modeled using the Hodgkin-Huxley formalism [110]. Specifically, the ionic current for channel *i*, *I*_*i*,_ was modeled as *I*_*i*_ = *g*_*i*_*m*^*p*^*h*^*q*^(*V* − *E*_*i*_), where *g*_*i*_ was its maximal conductance density, *m* its activation variable (with exponent *p*), *h* its inactivation variable (with exponent *q*), and *E*_*i*_ its reversal potential. The *I*_CAN_ current utilized a slightly modified equation [114]: *I*_*CAN*_ = *g*_*CAN*_*M*([*Ca*]_*i*_)*m*(*V* − *E*_*CAN*_), where *M*([*Ca*]_*i*_) = [*Ca*]_*i*_/(0.2 + [*Ca*]_*i*_) is a Michaelis-Menten function and [*Ca*]_i_ denotes the intracellular calcium concentration. The kinetic equation for the gating variable *x* (*m or h*) satisfied a first-order kinetic model:

$$\frac{dx}{dt}=\phi_{x}\frac{x_{\infty }(V,{\left[Ca\right]}_{i})-x}{\tau_{x}(V,{\left[Ca\right]}_{i})}$$ (2)

where *ϕ*_*x*_ is a temperature-dependent factor, *x*_*∞*_ is the voltage or Ca^2+^- dependent steady state and *τ*_*x*_ is the voltage or Ca^2+^- dependent time constant in msec. Equivalently, Eq (2) can be written as:

$$\frac{dx}{dt}=\phi_{x}(\alpha_{x}(V,{\left[Ca\right]}_{i})(1-x)-\beta_{x}(V,{\left[Ca\right]}_{i})x)$$ (3)

where *α*_x_ and *β*_x_ are the voltage or Ca^2+^- dependent rate constants with dimension of msec^-1^. The maximal conductance densities of all ionic currents and the kinetic parameters of all gating variables for all four types of neurons are listed in S1 and S2 Tables, respectively. The sodium reversal potential was set to *E*_Na_ = 50 mV and the potassium to *E*_K_ = -90 mV. The reversal potentials for *I*_H_ and *I*_CAN_ currents were *E*_H_ = -43 mV [113] and *E*_CAN_ = 10 mV [114] respectively. The calcium reversal potential (*E*_Ca_) was dynamically determined by the Nernst equation in all cell types in the model [118]:

$$E_{Ca}=\frac{RT}{2F}log\left(\frac{{\left[{Ca}^{2+}\right]}_{o}}{{\left[{Ca}^{2+}\right]}_{i}}\right)$$ (4)

where *R* = 8.31441 J/(mol°K), *T* = 309.15°K, *F* = 96,489 C/mol, and [Ca^2+^]_o_ = 2 mM.

### Calcium dynamics

Intracellular calcium was regulated by a simple first-order differential equation of the form [118, 119]:

$$\frac{d{\left[Ca^{2+}\right]}_{i}}{dt}=-\frac{I_{Ca}}{zFw}+\frac{{\left[Ca^{2+}\right]}_{rest}-{\left[Ca^{2+}\right]}_{i}}{\tau_{Ca}}$$ (5)

where *I*_Ca_ is the summation of all Ca^2+^ currents, *w* is the thickness of the perimembrane “shell” in which calcium is able to affect membrane properties (0.5 μm), z = 2 is the valence of the Ca^2+^ ion, *F* is the Faraday constant, and *τ*_*Ca*_ is the Ca^2+^ removal rate (10 ms for HTC, RTC and IN cells; 100 ms for RE cells). The resting Ca^2+^ concentration was set to be [*Ca*^2+^]_*rest*_ = .05 μM.

### Synaptic currents and short-term synaptic depression

The gap junction current was computed as *I*_*gap*_ = (*V*_*post*_ − *V*_*pre*_)/*R*_*g*_, where *V*_*pre*_ and *V*_*post*_ are the membrane potentials of the presynaptic and postsynaptic neurons respectively. Gap junctional coupling was stronger among HTC cells than between HTC and RTC cells [17]. Accordingly, the gap junction resistance *R*_*g*_ was smaller for the HTC-HTC synapses (100 MΩ) than for the HTC-RTC synapses (300 MΩ). The coupling strength between RE cells was set to be the same as that between HTC and RTC cells (*R*_*g*_ = 300 MΩ). These gap junction resistance values were selected to match the experimental data [12, 17, 94]. In the model, glutamatergic synaptic current was mediated by both AMPA and NMDA receptors, while GABAergic synaptic current was mediated by GABA_A_ receptors. The synaptic current was calculated by the following equation [119, 120]:

$$I_{syn}=g_{syn}sB(V)(V-E_{syn})$$ (6)

where *g*_syn_ is the maximal synaptic conductance and *E*_syn_ is the synaptic reversal potential. The default maximal conductances were: *g*_*AMPA*_ = 6 nS and *g*_*NMDA*_ = 3 nS for HTC→IN synapses, and *g*_*AMPA*_ = 4 nS and *g*_*NMDA*_ = 2 nS for the TC→RE synapses. The synaptic strength from inhibitory neurons (INs and REs) to TC cells was assumed to be higher than that among inhibitory neurons: *g*_*GABA*_ = 3 nS for IN→RTC and RE→TC synapses while *g*_*GABA*_ = 1 nS for both RE→IN and RE→RE synapses. *E*_syn_ = 0 mV for AMPA and NMDA currents, and *E*_syn_ = -80 mV for GABA_A_ receptors in TC cells, while *E*_syn_ = -70 mV for GABA_A_ receptors in RE neurons [106, 121]. The function *B*(V), which implements the Mg^2+^ block for NMDA currents, was defined as *B*(*V*) = 1/(1 + exp(−(*V* + 25)/12.5) [86, 112]. For AMPA and GABA_A_ currents, *B*(V) = 1. The gating variable *s* represents the fraction of open synaptic ion channels and obeys a first-order kinetics [82, 83, 122]:

$$\frac{ds}{dt}=\alpha [T](1-s)-\beta s$$ (7)

where [T] is the concentration of neurotransmitter in the synapse and *α* and *β* are forward and backward binding rates. The neurotransmitter is assumed to be a brief pulse that has duration of 0.3 ms and amplitude of 0.5 mM following an action potential in the presynaptic neuron [57]. The channel opening rate constants (*α* and *β*) are given as: *α* = 0.94 ms^-1^, *β* = 0.18 ms^-1^ for AMPA receptor current, *α* = 1 ms^-1^, *β* = 0.0067 ms^-1^ for NMDA receptor current and *α* = 10.5 ms^-1^, *β* = 0.166 ms^-1^ for GABA_A_ receptor current. These values were taken from previous modeling studies [57, 86, 118]. A synaptic delay of 2 ms was introduced in all chemical synapses.

Short-term synaptic depression was implemented in all chemical synapses and was modeled by scaling the maximal conductance of a given synaptic channel by a depression variable *D*, which represented the amount of available “synaptic vesicles” [86, 87]. The variable *D* was updated according to a simple phenomenological rule [86, 123]:

$$D=1-(1-D_{i}(1-U))exp(-\frac{t-t_{i}}{\tau })$$ (8)

where U = 0.07 is the fraction of resources used per action potential, τ = 700 ms is the time constant of recovery of the synaptic vesicles. *D*_i_ is the value of *D* immediately before the i_th_ presynaptic spike and *t*_i_ is the timing of the i_th_ spike event.

### External inputs and neuronal heterogeneity

All neurons in the thalamic network received independent Poisson-distributed spike inputs at an average rate of 100 Hz (results maintained unchanged if higher input rates were used when also scaling down the maximal synaptic input conductance). These random inputs represented both extrinsic sources of background noise and asynchronous visual input. This input was exclusively mediated by AMPA receptors modeled as an instantaneous step followed by an exponential decay with a time constant of 5 ms [124]. The synaptic input weights (i.e., maximal synaptic conductance) for all neuronal types during different oscillatory states are given in Table 1. Spindle oscillations were triggered by a transient input (100 ms × 100 pA) injected into RE neurons which represented a cortical UP state or a surge of synchronized background inputs.

To introduce heterogeneity into the model neurons, the leakage conductance (*g*_L_) of all neurons in the network was drawn from a uniform distribution within ±25% of the default value (i.e., 0.0075–0.0125 mS/cm^2^). This leak conductance variation, random synaptic connectivity, and random external inputs constituted the model noise in the thalamic network.

### Model hypothesis

Our central modeling hypothesis is that the generation and transition of distinct thalamic oscillatory states are functions of both ACh/NE neuromodulation and afferent excitation level (Fig 1B). Main motivations were the fact that different oscillatory states appear under different behavioral conditions in the sleep-wakefulness cycle (Table 1) and that the transition from sleep to wakefulness is controlled mainly by activation of both cholinergic and noradrenergic neuromodulatory systems [25, 26, 125]. The thalamic oscillatory state transition is also a function of afferent excitation since thalamic neurons receive stronger afferent inputs during wakefulness than sleep due to activation of the sensory systems. Accordingly, we modeled four distinct oscillations (δ, spindle, α and γ) under three different ACh/NE modulation states (low, medium and high) corresponding to deep sleep, light sleep and awake conditions (Table 1). Specifically, δ oscillations were modeled in low ACh/NE modulation state with minimal afferent excitation; spindle oscillations were modeled in the medium ACh/NE modulation state with slight afferent excitation; and α and γ oscillations were modeled in the high ACh/NE modulation state with weak and strong afferent excitation respectively (Table 1). The two levels of afferent excitation in the high ACh/NE modulation state modeled two different behavioral conditions: awake with eyes closed (where α oscillations are maximal) and awake with eyes open and with attention. The effect of ACh/NE modulation was modeled by varying the potassium leak conductance in all four types of thalamic neurons while different afferent excitation was modeled by changing the maximal synaptic conductance of the random Poisson inputs to thalamic cells (Table 1). The rationale and selection of specific parameter values during each oscillatory state for both the potassium leak conductance and synaptic input conductance are described below.

### Simulated effects of ACh and NE in thalamic neurons

Acetylcholine (ACh) and norepinephrine (NE) alter the intrinsic excitability of thalamic neurons mainly by modulating the potassium leak current [25, 26, 101, 126–128]. Both ACh and NE directly depolarize TC cells via blocking the potassium leak current [25, 101, 127]. By comparison, ACh inhibits LGN local interneurons and RE cells by activating the potassium leak current via muscarinic receptor activation [126, 128]. In contrast, application of NE or stimulation of the locus coeruleus enhances the excitability of RE neurons by reducing the potassium leak current [25, 26, 127]. The combined effect of ACh and NE on RE cells is inferred from experimental data showing that a progressive hyperpolarization occurred in RE neurons during the transition from arousal to quite wakefulness and to deeper states of EEG-synchronized sleep [61, 129]. We thus assumed that the excitatory effect of NE dominated the inhibitory effect of ACh on RE neurons so that the potassium leak current was decreased during transition from sleep to wakefulness. Also, since the action of NE on LGN interneurons remains unknown [25, 26], the ACh/NE neuromodulatory effect on interneurons was assumed to be mediated by cholinergic action only.

### Model parameters for different oscillatory states

#### Delta oscillations

Thalamic delta waves occur during the deep stage of sleep where the membrane potentials of TC and RE neurons are most hyperpolarized [31], presumably as a result of the inactivation of the cholinergic and noradrenergic systems [28]. Thus, the potassium leak conductance in TC and RE cells was the highest among the three neuromodulatory states (TC: 0.035 mS/cm^2^; RE: 0.03 mS/cm^2^; Table 1). In contrast, the potassium leak conductance in INs was the smallest among the three states of neuromodulation (0.01 mS/cm^2^; Table 1) since the absence of ACh reduced the potassium leak current, as mentioned earlier. The random input strength (0.1 nS) was the lowest among all four oscillatory states because of inactivation of both the sensory and neuromodulatory systems during the deep sleep stage (Table 1).

#### Spindle oscillations

Since spindle oscillations occur during earlier sleep stages than delta waves [61], the level of ACh/NE modulation is higher than that during the deep sleep stage, but lower than that during the awake stage. Correspondingly, for TC cells the potassium leak conductance was decreased from 0.035 mS/cm^2^ during the deep sleep stage (low modulation state) to 0.01 mS/cm^2^ during the light sleep stage (medium modulation state; Table 1). As higher ACh level increases the potassium leak current in INs, the potassium leak conductance was increased from 0.01 mS/cm^2^ during the deep sleep stage to 0.015 mS/cm^2^ during the light sleep stage. For RE neurons, the potassium leak conductance was decreased from 0.03 mS/cm^2^ during the deep sleep stage to 0.02 mS/cm^2^ during the light sleep stage as we assumed that the concerted action of ACh and NE was to reduce the potassium leak current in RE cells. In the meantime, the random input strength (to all four types of neurons) was increased from 0.1 nS during deep sleep stage to 0.3 nS during the light sleep stage (Table 1).

#### Alpha oscillations

Since α oscillations appear in the awake state, ACh/NE level is assumed to be high. This is consistent with experimental finding that muscarinic cholinergic receptor (mAChR) activation induced synchronized α oscillations in LGN slice preparation [17, 18]. Alpha oscillation was thus modeled by fully blocking the potassium leak current in TC cells, representing the concerted action of high level of ACh and NE modulation during wakefulness. Similarly, the potassium leak conductance in RE cells decreased from 0.02 mS/cm^2^ in the medium modulation state during light sleep to 0.01 mS/cm^2^ in the high modulation state during wakefulness (Table 1). On the other hand, to model the inhibitory effect of ACh on interneurons, the potassium leak conductance was increased from 0.015 mS/cm^2^ in the medium modulation state to 0.02 mS/cm^2^ in the high modulation state (Table 1). The random input strength substantially increased from 0.3 nS (during light sleep) to 1.5 nS during wakefulness, which was assumed to be the same for all four types of neurons (Table 1)

#### Gamma oscillations

As fast γ oscillations occur during the awake state, the level of ACh and NE modulation was assumed to be the same as that during α oscillations. The switch from α to γ oscillations was modeled by a marked increase in the random input intensity to both HTC and RTC cells which mimicked the change from “eyes closed” to “eyes open” and the additional attentional input (from the cortex): *g*_*input*_ = 17 nS for γ oscillations (Table 1). The random input strength to both IN and RE cells was maintained at the same level (1.5 nS) as during α oscillations.

### Stimulation protocol

We selected a periodic pulsatile stimulus that conceptually resembles the waveform of deep brain stimulation (DBS) or repetitive transcranial magnetic stimulation (rTMS) [44]. The stimulation was assumed to be global: all neurons in the LGN (TC & IN cells) received the same stimulus pattern. Stimulation consisted of a train of 10 ms brief square current pulse applied at different frequencies ranging from 1 Hz to 50 Hz with a 1 Hz/step increment. The stimulation amplitude was fixed at 0.2 nA. Stimulation was performed on three major oscillatory states (δ, α and γ oscillations) and stimulation at each frequency lasted for 1 second.

### Data analysis

#### Simulated local field potential

A simulated local field potential (sLFP) was constructed by filtering the average membrane potentials across all TCs [84, 119, 130]. Filtering was carried out numerically using a band-pass filter (0.5–80 Hz) with the MATLAB functions FIR1 and FILTFILT [130]. The power spectrum of the signal was obtained by a fast Fourier transform (FFT) of the filtered sLFP. The network oscillation frequency was determined by the peak spectral frequency. During stimulation, the frequency power spectrum was calculated during each stimulation frequency step (i.e., 1 second) and the power spectrum heat map was generated using the MATLAB function IMAGESC. Neuronal spike times were converted to spike phases as detailed in [119]. Also, to compare with the experimental data, the sign of the sLFP was reversed (-sLFP) to calculate the spike phases [84].

#### Synchronization and correlation indices

The synchronization index was calculated as follows [119]:

$$\kappa =1/N\sqrt{{\left[\sum_{i=1}^{N}sin(\varphi_{i})\right]}^{2}+{\left[\sum_{i=1}^{N}cos(\varphi_{i})\right]}^{2}}$$ (9)

where φ_i_ was the phase of each spike relative to the sLFP peak and *N* was the total number of spikes for a particular neuronal population. This metric measures the degree of phase-locking between spikes and sLFP: when all spikes have identical phases, the index achieves its maximal value of unity.

To compute the correlation index, we first generated the peri-event time histogram (PETH) for each of the four neuronal populations (HTC, RTC, IN and RE) by dividing the simulation time interval into small bins (2 ms) and summing up the number of spikes in each bin. The correlation index between two groups of neuronal populations was determined as the peak of the cross-correlation between the mean-removed PETH of the two corresponding neuronal groups. The correlation index for the whole network was calculated as the mean of the respective indices for all six pairs of neuronal populations within the thalamic network.

#### Oscillation state transition map

To generate the oscillation state transition map, we divided the ACh/NE modulation into 11 levels evenly ranging from 0% to 100% with 10% increment. The 0% ACh/NE modulation corresponded to the potassium leak conductances in the deep sleep stage (HTC & RTC: 0.036 mS/cm^2^; IN: 0.01 mS/cm^2^; RE: 0.03 ms/cm^2^; note the conductances for HTC & RTC cells were slightly increased compared to Table 1) while the 100% ACh/NE modulation corresponded to the potassium leak conductances in the awake state (HTC & RTC: 0.0 mS/cm^2^; IN: 0.02 mS/cm^2^; RE: 0.01 ms/cm^2^; Table 1). The conductance values for other levels of ACh/NE were obtained through linear interpolation between 0% and 100% ACh/NE. In addition, we varied the maximal input conductance to TC cells from 0 up to 20 nS with a 0.5 nS step, which resulted in 451 different parameter combinations in term of ACh/NE level and afferent input level. We then simulated the thalamic network for each parameter combination for 3 seconds and classified the oscillatory state of the network accordingly. The classification process involved several steps. First, spontaneous spindle oscillations (induced spontaneously by random Poisson inputs to TCs) were classified which need to satisfy the following conditions: (1) The sLFP oscillation power (spectral peak) during the 1-second period before spindle oscillations is below a threshold (1.0); (2) The sLFP oscillation power during spindle oscillations (1 second) was above a threshold (3.0); and (3) The oscillation frequency is between 7 and 15 Hz. Second, after the spontaneous spindle oscillations were classified, the state of the remaining networks was determined using the frequency power spectrum of the sLFP during the last 1 second of the 3-second simulation interval. If the oscillation power was above a threshold (1.0), the oscillation state was classified according to the dominant oscillation frequency (δ: 1–4 Hz; θ: 4–8 Hz; α: 8–14 Hz; β: 14–30 Hz; γ: > 30 Hz). If the oscillation power was below a threshold (1.0), the network was temporally classified as non-oscillatory. Lastly, a transient (100 ms) spindle triggering input (see above) was applied to RE neurons at 1000 ms for the networks temporally classified as non-oscillatory. If the transient input induced a train of oscillations that lasted for more than 500 ms, whose power was larger than a threshold (3.0) and whose frequency was between 7 and 15 Hz, the network state was reclassified as spindle oscillation. Otherwise, the network state was finally set to non-oscillatory.

### Numerical method

The thalamic network model was coded with C++. All simulation were performed using the fourth-order Runge-Kutta (RK4) method with a fixed time step of 0.02 ms. Shorter simulation step did not change the results. The major simulation results were validated in a separate model implementation using the Brian simulator [131]. Simulations were run on a Dell Linux workstation under Ubuntu. The model source codes are available in the ModelDB database (https://senselab.med.yale.edu/modeldb/).

## Supporting information

S1 Text Firing patterns of single thalamic model neurons. (DOCX)

S1 Table Maximal conductance densities (mS/cm^2^) of active ionic currents in the HTC, RTC, IN and RE model cells. (DOCX)

S2 Table Kinetics of gating variables for each channel implemented in the HTC, RTC, IN and RE model cells. (DOCX)

S1 Fig Firing patterns of the HTC and RTC model cells in three different ACh/NE modulatory states. **(A)** Voltage responses of the HTC model cell. **(A1)** Voltage responses of the HTC model cell to three levels of current injection (0 pA, 300 pA and 500 pA; 0–2000 ms) in the low ACh/NE modulatory state. Note that HTC cell generates spontaneous low-threshold bursting. **(A2)** Voltage responses of the HTC model cell to three levels of current injection (-50 pA, 100 pA and 200 pA; 500–1500 ms) in the medium ACh/NE modulatory state. (**A3)** Voltage responses of the HTC model cell to three levels of current injection (0 pA, 30 pA and 100 pA; 0–2000 ms) in the high ACh/NE modulatory state. Note that HTC cell generates spontaneous high-threshold bursting.
**(B)** Voltage responses of the RTC model cell. **(B1)** Voltage responses of the RTC model cell to three levels of current injection (0 pA, 300 pA and 500 pA; 0–2000 ms) in the low ACh/NE modulatory state. **(B2)** Voltage responses of the RTC model cell to three levels of current injection (-50 pA, 100 pA and 200 pA; 500–1500 ms) in the medium ACh/NE modulatory state. **(B3)** Voltage responses of the RTC model cell to three levels of current injection (-100 pA, 100 pA and 200 pA; 500–1500 ms) in the high ACh/NE modulatory state. For both HTC and RTC cells, *g*_*KL*_ = 0.035 mS/*cm^2^* in the low ACh/NE modulatory state; *g*_*KL*_ = 0.01 mS/*cm^2^* in the medium ACh/NE modulatory state and *g*_*KL*_ = 0.0 mS/*cm^2^* in the high ACh/NE modulatory state.
(TIF)

S2 Fig Firing patterns of the IN and RE model cells in three different ACh/NE modulatory states. **(A)** Voltage responses of the IN model cell. **(A1)** Voltage responses of the IN model cell to three levels of current injection (50 pA, 100 pA and 200 pA; 500–1500 ms) in the low ACh/NE modulation state. **(A2)** As (**A1)**, but in the medium ACh/NE modulation state. **(A3)** As (**A1)**, but in the high ACh/NE modulation state. For the low ACh/NE modulation state, *g*_*KL*_ = 0.01 mS/*cm^2^*; for the medium ACh/NE modulation state, *g*_*KL*_ = 0.015 mS/*cm^2^*; and for the high ACh/NE modulation state, *g*_*KL*_ = 0.02 mS/*cm^2^*.
**(B)** Voltage responses of the RTC model cell. **(B1)** Voltage responses of the RE model cell to three levels of current injection (-50 pA, 100 pA and 300 pA; 500–1500 ms) in the low ACh/NE modulation state. **(B2)** As **(B1)**, but in the medium ACh/NE modulation state. **(B3)** As **(B1)**, but in the high ACh/NE modulation state. For the low ACh/NE modulation state, *g*_*KL*_ = 0.03 mS/*cm^2^*; for the medium ACh/NE modulation state, *g*_*KL*_ = 0.02 mS/*cm^2^*; and for the high ACh/NE modulation state, *g*_*KL*_ = 0.01 mS/*cm^2^*.
(TIF)

S3 Fig Delta oscillation frequency can be reduced by adjusting the leak/potassium leak current (*I*_L_/*I*_KL_) and the low-threshold T-type Ca^2+^ current (*I*_Ca/T_) in TC cells. **(A)** Delta oscillation frequency is reduced to about 3 Hz (controls: 3.7 Hz) when the potassium leak conductance in TC cells slightly increases to 0.037 mS/cm^2^ (controls: 0.035 mS/cm^2^). **(A1)** Membrane voltage of representative HTC, IN, RTC and RE neurons. **(A2)** Spike rastergrams of HTC, IN, RTC and RE cells. **(A3)** Simulated LFP (*top*) with associated frequency power spectrum (*bottom*).
**(B)** Delta oscillation frequency is reduced to about 2 Hz (controls: 3.7 Hz) when the regular leak conductance in TC cells is reduced substantially to 0.001 mS/cm^2^ (controls: 0.01 mS/cm^2^), the potassium leak conductance in TC cells increases to 0.04 mS/cm^2^ (controls: 0.035 mS/cm^2^), and the inactivation time constant of the low-threshold T-type Ca^2+^ current (*I*_Ca/T_) increases 25%. **(B1)** Membrane voltage of representative HTC, IN, RTC and RE neurons. **(B2)** Spike rastergrams of HTC, IN, RTC and RE cells. **(B3)** Simulated LFP (*top*) with associated frequency power spectrum (*bottom*).
(TIF)

S4 Fig Effect of possible NE modulation on INs during spindle oscillations. **(A)** Spindle oscillations during the control condition when the NE modulatory effect on INs is neglected (*g*_*KL*_ = 0.015 mS/cm^2^). **(A1)** Membrane voltages of representative HTC, IN, RTC and RE cells. **(A2)** Spike rastergrams of HTC, IN, RTC and RE cells. **(A3)** Simulated LFP (*top*) with associated frequency power spectrum (*bottom*).
**(B)** Spindle oscillations when the NE modulatory effect on INs counteracts the effect of ACh (*g*_*KL*_ = 0.01 mS/cm^2^). **(B1)** Membrane voltages of representative HTC, IN, RTC and RE cells. **(B2)** Spike rastergrams of HTC, IN, RTC and RE cells. **(B3)** Simulated LFP (*top*) with associated frequency power spectrum (*bottom*).
**(C)** Spindle oscillations when the NE modulatory effect on INs overcomes the effect of ACh (*g*_*KL*_ = 0.005 mS/cm^2^). **(C1)** Membrane voltages of representative HTC, IN, RTC and RE cells. **(C2)** Spike rastergrams of HTC, IN, RTC and RE cells. **(C3)** Simulated LFP (*top*) with associated frequency power spectrum (*bottom*).
(TIF)

S5 Fig Effect of possible NE modulation on INs during alpha oscillations. **(A)** Alpha oscillations during the control condition when the NE modulatory effect on INs is neglected (*g*_*KL*_ = 0.02 mS/cm^2^). **(A1)** Membrane voltages of representative HTC, IN, RTC and RE cells. **(A2)** Spike rastergrams of HTC, IN, RTC and RE cells. **(A3)** Simulated LFP (*top*) with associated frequency power spectrum (*bottom*).
**(B)** Alpha oscillations when the NE modulatory effect on INs counteracts the effect of ACh (*g*_*KL*_ = 0.01 mS/cm^2^). **(B1)** Membrane voltages of representative HTC, IN, RTC and RE cells. **(B2)** Spike rastergrams of HTC, IN, RTC and RE cells. **(B3)** Simulated LFP (*top*) with associated frequency power spectrum (*bottom*).
**(C)** Alpha oscillations when the NE modulatory effect on INs overcomes the effect of ACh (*g*_*KL*_ = 0.0 mS/cm^2^). **(C1)** Membrane voltages of representative HTC, IN, RTC and RE cells. **(C2)** Spike rastergrams of HTC, IN, RTC and RE cells. **(C3)** Simulated LFP (*top*) with associated frequency power spectrum (*bottom*).
(TIF)

S6 Fig Effect of possible NE modulation on INs during gamma oscillations. **(A)** Gamma oscillations during the control condition when the NE modulatory effect on INs is neglected (*g*_*KL*_ = 0.02 mS/cm^2^). **(A1)** Membrane voltages of representative HTC, IN, RTC and RE cells. **(A2)** Spike rastergrams of HTC, IN, RTC and RE cells. **(A3)** Simulated LFP (*top*) with associated frequency power spectrum (*bottom*).
**(B)** Gamma oscillations when the NE modulatory effect on INs counteracts the effect of ACh (*g*_*KL*_ = 0.01 mS/cm^2^). **(B1)** Membrane voltages of representative HTC, IN, RTC and RE cells. **(B2)** Spike rastergrams of HTC, IN, RTC and RE cells. **(B3)** Simulated LFP (*top*) with associated frequency power spectrum (*bottom*).
**(C)** Gamma oscillations when the NE modulatory effect on INs overcomes the effect of ACh (*g*_*KL*_ = 0.0 mS/cm^2^). **(C1)** Membrane voltages of representative HTC, IN, RTC and RE cells. **(C2)** Spike rastergrams of HTC, IN, RTC and RE cells. **(C3)** Simulated LFP (*top*) with associated frequency power spectrum (*bottom*).
(TIF)

S7 Fig High level of RE synchronization is not required for coherent α oscillations. **(A)** Spike rastergrams of HTC, IN, RTC and RE cells when the TC→RE synaptic strength is reduced by 50% (AMPA: from 4 nS to 2 nS; NMDA: from 2 nS to 1 nS) and the random input strength to RE cells increases twofold (from 1.5 nS to 3 nS).
**(B)** Distribution of spike phase relative to sLFP peaks for HTC, IN, RTC and RE cells under the same parameter changes as **(A)**.
**(C)** Simulated LFP (*top*) with associated frequency power spectrum (*bottom*) under the same parameter changes as **(A)**.
(TIF)

S8 Fig Thalamic α oscillations are abolished when gap junctions among TC cells are blocked in the network. **(A)** Thalamic network activities with intact gap junctions. **(A1)** Membrane voltages of two representative HTC, IN, RTC and RE cells each in the control case. **(A2)** Spike rastergrams of HTC, IN, RTC and RE cells in the control case. **(A3)** Simulated LFP (*top*) with associated frequency power spectrum (*bottom*) in the control case.
**(B)** Thalamic network activities without gap junctions. **(B1-B3)** As **(A1-A3)**, but when gap junctions among TC cells (HTC-HTC & HTC-RTC) are blocked.
(TIF)

S9 Fig IN neurons become phase-locked to the γ rhythm when the short-term depression (STD) at the HTC→IN synapses is removed. **(A)** INs are not phase locked to the γ rhythm in the control case. **(A1)** Spike rastergrams of HTC, IN, RTC and RE cells. **(A2)** Distribution of spike phases relative to sLFP peaks for HTC, IN, RTC and RE cells.
**(B)** INs are phase locked to the γ rhythm when the STD at HTC→IN synapses is blocked. **(B1-B2)** As **(A1-A2)**, but when the STD at HTC→IN synapses is blocked.
(TIF)

S10 Fig The thalamic network oscillation frequency is reduced to the β band (e.g., 23.2 Hz) when the synaptic input drive to TC cells is reduced (e.g., from 17 nS to 10 nS). **(A)** Membrane voltages of two representative HTC, IN, RTC and RE cells each.
**(B)** Spike rastergrams of HTC, IN, RTC and RE cells.
**(C)** Simulated LFP (*top*) with associated frequency power spectrum (*bottom*).
(TIF)

S11 Fig Thalamic δ oscillations are altered when specific gap junctions between TC cells are blocked. **(A)** Simulated LFP (*top*) with associated frequency power spectrum (*bottom*) when the gap junctions among HTC cells are blocked.
**(B)** As **(A)**, but when both HTC-HTC and HTC-RTC gap junctions are blocked.
(TIF)

S12 Fig Effect of increasing the excitatory TC→RE synaptic weight on spindle duration. **(A)** Membrane voltages of two representative HTC, IN, RTC and RE cells each in the control condition.
**(B)** Membrane voltage of two representative HTC, IN, RTC and RE cells each when the excitatory TC→RE synaptic weight increases to 150% of its default value (AMPA: from 4 nS to 6 nS; NMDA: from 2 nS to 3 nS). The horizontal bar in the bottom panel indicates the injection of a transient current input (100 ms × 100 pA) into RE neurons to trigger spindle oscillations.
(TIF)

S13 Fig Spontaneous spindle oscillations under low ACh/NE modulation (0%) and with moderate afferent input (6.5 nS). **(A)** Membrane voltages of two representative HTC, IN, RTC and RE cells each.
**(B)** Spike rastergrams of HTC, IN, RTC and RE cells.
**(C)** Simulated LFP (*top*) with associated frequency power spectrum (*bottom*).
(TIF)

S14 Fig HTC cells burst randomly under low ACh/NE modulation (0%) and with moderate level input (5 nS) when gap junctions between HTCs are blocked. **(A)** Membrane voltages of two representative HTC, IN, RTC and RE cells each.
**(B)** Spike rastergrams of HTC, IN, RTC and RE cells.
(TIF)

S15 Fig The thalamic network becomes desynchronized under low ACh/NE modulation (0%) when the afferent input is strong (20 nS). **(A)** Membrane voltages of two representative HTC, IN, RTC and RE cells each.
**(B)** Spike rastergrams of HTC, IN, RTC and RE cells.
(TIF)

S16 Fig The duration of spindle oscillations reduces when the afferent input (to TCs) increases under medium level of ACh/NE modulation (50%). **(A)** Membrane voltages of two representative HTC, IN, RTC and RE cells each when *g*_*Input*_ = 1.5 nS.
**(B)** Membrane voltages of two representative HTC, IN, RTC and RE cells each when *g*_*Input*_ = 2.0 nS.
**(C)** Membrane voltages of two representative HTC, IN, RTC and RE cells each when *g*_*Input*_ = 2.5 nS.
(TIF)

S17 Fig Thalamic network activity during the first harmonic entrainment (1:2) of γ oscillations. **(A)** Top four panels: spike rastergrams of HTC, IN, RTC and RE cells; bottom panel: stimulation waveform.
**(B)** Simulated LFP (*top*) with associated frequency power spectrum (*bottom*).
(TIF)

S18 Fig High frequency stimulation of α oscillations suppresses oscillation power. **(A)** Thalamic network activity during α oscillations without stimulation. **(A1)** Spike rastergrams of HTC, IN, RTC and RE cells (*top four panels*). **(A2)** Simulated LFP (*top*) with associated frequency power spectrum (*bottom*).
**(B)** Thalamic network activity during 40 Hz stimulation of α oscillations. **(B1)** Spike rastergrams of HTC, IN, RTC and RE cells (*top four panels*) with stimulation waveform (*bottom panel*). **(B2)** Simulated LFP (*top*) with associated frequency power spectrum (*bottom*). The stimulation amplitude is 200 pA (0.2 nA).
(TIF)
