# Supplementary material for: Unified thalamic model generates multiple distinct oscillations with state-dependent entrainment by stimulation
Source: PLoS Comput Biol. 2017 Oct 26;13(10):e1005797. doi: 10.1371/journal.pcbi.1005797 (PMC5675460; doi:10.1371/journal.pcbi.1005797)
Supplement: S1 Text — (DOCX) [file pcbi.1005797.s001.docx]

**Supporting Information**

**S1 Text. Firing patterns of single thalamic model neurons**

*High-threshold bursting TC cell*

In the low ACh/NE modulation state (similar to the control condition), the HTC model cell fired intrinsic low-threshold bursts (LTBs) at 3.4 Hz in the absence of current injection (S1A1 Fig, *bottom*). Depolarization of the HTC model cell by a large positive current injection (500 pA) switched intrinsic LTBs to tonic spiking (S1A1 Fig, *top*) and there also existed a quiescent resting state during the transition from LTBs to tonic spiking (S1A1 Fig, *middle*), consistent with TC cell physiological data [1-4]. In the medium ACh/NE modulation state, the HTC model cell rested at around -64 mV without afferent input (S1A2 Fig; first 500 ms before current injection). Hyperpolarization of the HTC model cell by a negative current injection (-50 pA) led to LTBs during hyperpolarization as well as rebound LTBs on the release of the hyperpolarizing input (S1A2 Fig, *bottom*), as observed experimentally [1-3, 5]. On the other hand, depolarizing current injection (100 pA) produced high-threshold bursts (HTBs) (S1A2 Fig, *middle*) due to activation of the high-threshold T-type Ca^2+^ current (*I*_Ca/HT_). The HTBs were converted to tonic spiking when a larger injected current (200 pA) was applied (S1A2 Fig, *top*). In the high ACh/NE modulation state, the HTC cell produced spontaneous HTBs with an inter-burst frequency of 6.7 Hz at depolarized membrane potentials (>-55 mV) (S1A3 Fig, *bottom* ), consistent with experimental data [6, 7]. The HTB frequency increased to 9.2 Hz with small depolarization (30 pA; S1A3 Fig, *middle*), and eventually switched to tonic spiking with larger depolarization (100 pA; S1A3 Fig, *top*). Thus, the HTC model cell exhibited three distinct firing modes, LTB/rebound LTB, HTB and tonic spiking, dependent on the ACh/NE modulation state as well as on the afferent depolarization level. These three firing modes covered the range of activities observed in these cells both *in vitro* [4, 5, 7, 8] and in *vivo* [9].

*Relay-mode TC cell*

The firing properties of the RTC model cell are shown in S1B Fig. Similar to the HTC cell, the RTC model cell also displayed two firing modes in the low ACh/NE modulation state (S1B1 Fig). They fired spontaneous LTBs in the δ frequency range (3 Hz) (S1B1 Fig, *bottom*) and tonic spiking with high levels of depolarization (S1B1 Fig, *top*). There also existed a quiescent resting state during the transition from LTBs to tonic spiking (S1B1 Fig, *middle*). In the medium ACh/NE modulation state, the RTC model cell rested at about -64 mV (first 500 ms; S1B2 Fig) where a small hyperpolarizing current (-50 pA) produced LTBs/rebound LTBs (S1B2 Fig, *bottom*) and a larger depolarizing input (200 pA) induced tonic spiking (S1B2 Fig, *top*), similar to the responses of the HTC model cell (S1A2 Fig, *bottom and top*). However, unlike the HTC model cell, a medium depolarizing current injection (100 pA) failed to produce HTBs in the RTC model cell (compare S1B2 Fig with S1A2 Fig, *middle*) because of the much smaller *I*_Ca/HT_ conductance in RTC cell (S1 Table). In the high ACh/NE modulation state, the resting potential of the RTC model cell was raised to -55.7 mV, but no HTBs were generated either spontaneously or with depolarizing current (S1B3 Fig). Similar to the medium ACh/NE modulation state (S1B2 Fig), a hyperpolarizing current (-100 pA) induced LTBs/rebound LTBs (S1B3 Fig, *bottom*) while depolarizing inputs (100 & 200 pA) led to tonic spiking (S1B3 Fig, *top and middle*). Due to increased excitability, it was noted that the same medium depolarizing input (100 pA) that failed to generate spiking activity in the medium ACh/NE modulation state was able to produce tonic spiking under the high ACh/NE modulation state (compare S1B3 Fig with S1B2 Fig, *middle*). Overall, the RTC model cell replicated the classic dual firing modes of TC neurons and the excitability increased with ACh/NE modulation [4, 7, 8, 10].

*IN neuron*

The interneurons in the rat LGN fire high-threshold bursts (HTBs) in response to depolarizing input which transform to tonic spiking with large depolarization [11, 12]. The firing patterns of the IN model cell are shown in S2A Fig. In the low ACh/NE modulation state, the IN neuron had a resting potential of -65.6 mV and a small depolarization (50 pA) generated HTBs in depolarized membrane potentials (>-56 mV) (S2A1 Fig, *bottom*). Larger depolarizing currents (100 & 200 pA) led to tonic spiking in relatively high frequency (19 Hz and 34 Hz respectively) (S2A1 Fig, *middle and top*). In medium ACh/NE modulation state, the resting membrane potential was decreased to -69.3 mV due to increase of the potassium leak current and HTBs persisted for small and medium current depolarization (50 & 100 pA; S2A2 Fig, *middle* and *bottom*). Similarly, further depolarization (200 pA) switched HTBs to tonic spiking (S2A2 Fig, *top*). In the high ACh/NE modulation state, a small depolarizing current (50 pA) failed to induce bursting (S2A3 Fig, *bottom*) and a medium level of depolarization (100 pA) produced HTBs (S2A3 Fig, *middle*) while higher depolarization (200 pA) resulted in tonic spiking (S2A3 Fig, *top*).

*RE neuron*

Similar to TC cells, RE neurons also operate in two distinct firing modes depending on the membrane potentials. At relatively depolarized membrane potentials (> -65 mV), depolarizing current injection evokes tonic discharges, whereas at hyperpolarized membrane potentials (< -70 mV), the same depolarizing input leads to high-frequency burst of action potentials (APs) mediated by the low-threshold T-type Ca^2+^ current [13-18]. Such membrane potential- dependent firing modes were well replicated in the RE model cell (S2B Fig). In the low ACh/NE modulation state, the resting membrane potential of RE cell was hyperpolarized to -80.3 mV (due to large potassium leak conductance) where a medium depolarizing current pulse (100 pA) evoked a strong burst of Ca^2+^ spikes (7 APs) followed by one single spike (S2B1 Fig, *middle*); larger depolarizing pulse (300 pA) produced a stronger Ca^2+^ burst (8 APs) followed by a train of action potential (S2B1 Fig, *top*). It was noted that a marked silent period existed between the burst discharge and the tonic spiking that followed in response to a 300 pA current pulse, closely resembling experimental data [16, 19]. Strong LTB (7 APs) was also generated when the RE cell was released from a hyperpolarizing current pulse (-50 pA; S2B1 Fig, *bottom*), as observed experimentally [15, 16, 18]. In the medium ACh/NE modulation state, the RE membrane potential was depolarized to -75.2 mV due to reduction of the potassium leak current. The membrane potential depolarization was associated with weaker LTBs evoked by depolarizing current pulses (compare S2B2 Fig with S2B1 Fig, *top and middle*). Also, for large current injection (300 pA), the silent period between the initial LTB and tonic spiking disappeared and the LTB was immediately followed by tonic spiking (compare S2B2 Fig with S2B1 Fig, *top*). Such voltage-dependent interval between LTB and tonic discharge was highly consistent with experimental observation [16]. Moreover, the release from a hyperpolarizing current pulse (-50 pA) resulted in a train of LTBs, in contrast to only one single LTB after the hyperpolarizing input (compare S2B2 Fig with S2B1 Fig, *bottom*). In the high ACh/NE modulation state, the membrane potential was further depolarized to -64.2 mV under which only tonic discharge was evoked in response to depolarizing current injection (100 & 300 pA; S2B3 Fig, *top and middle*).This was because the low threshold T-type Ca^2+^ current was inactivated by membrane potential depolarization. Interestingly, under the high ACh/NE modulation state where the membrane potential was more depolarized, release from the same hyperpolarizing current pulse (-50 pA) resulted in higher frequency and more robust LTBs than under the low and medium ACh/NE modulation states (compare S2B3 Fig with S2B1 Fig and S2B2 Fig, *bottom*), in agreement with experimental finding [17, 18].

**References**

1. Jahnsen H, Llinás R (1984) Electrophysiological properties of guinea-pig thalamic neurones: an in vitro study. *J Physiol* 349:205–226.
2. Jahnsen H, Llinás R (1984) Ionic basis for the electro-responsiveness and oscillatory properties of guinea-pig thalamic neurones in vitro. *J Physiol* 349: 227–247.
3. McCormick DA, Pape HC (1990) Properties of a hyperpolarization-activated cation current and its role in rhythmic oscillation in thalamic relay neurones. *J Physiol* 431: 291–318.
4. Leresche N, Lightowler S, Soltesz I, Jassik-Gerschenfeld D, Crunelli V (1991) Low-frequency oscillatory activities intrinsic to rat and cat thalamocortical cells. *J Physiol* 441:155–174.
5. Dossi RC, Nunez A, Steriade M (1992) Electrophysiology of a slow (0.5–4 Hz) intrinsic oscillation of cat thalamocortical neurones in vivo. *J Physiol* 447: 215–234.
6. Hughes SW, Lörincz M, Cope DW, Blethyn KL, Kékesi KA, Parri HR, Juhász G, Crunelli V (2004) Synchronized oscillations at alpha and theta frequencies in the lateral geniculate nucleus *Neuron*, 42: 253–268.
7. Lorincz ML, Crunelli V, Hughes SW (2008) Cellular dynamics of cholinergically-induced alpha (8-13 Hz) rhythms in sensory thalamic nuclei in vitro. *J Neurosci* 28: 660-671.
8. Llinás R, Jahnsen H (1982) Electrophysiology of mammalian thalamic neurones in vitro. *Nature*, 297: 406–408.
9. Lorincz ML, Kekesi KA, Juhasz G, Crunelli V, Hughes SW (2009) Temporal framing of thalamic relay-mode firing by phasic inhibition during the alpha rhythm. *Neuron* 63: 683-696.
10. McCormick DA (1992) Cellular mechanisms underlying cholinergic and noradrenergic modulation of neuronal firing mode in the cat and guinea pig dorsal lateral geniculate nucleus. *J Neurosci* 12:278–289.
11. Zhu JJ, Lytton WW, Xue J-T, Uhlrich DJ (1999) An intrinsic oscillation in interneurons of the rat lateral geniculate nucleus. *J Neurophysiol* 81:702–711.
12. Zhu JJ, Uhlrich DJ, Lytton WW (1999) Burst firing in identified rat geniculate interneurons. *Neuroscience* 92:1445–1460.
13. Mulle C, Madariaga A, Deschênes M (1986) Morphology and electrophysiological properties of reticularis thalami neurons in cat: in vivo study of a thalamic pacemaker. *J Neurosci* 6:2134–2145.
14. Avanzini G, de Curtis M, Panzica F, Spreafico R (1989) Intrinsic properties of nucleus reticularis thalami neurones of the rat studied in vitro. *J Physiol* 416: 111–122.
15. Contreras D, Dossi RC, Steriade M (1992) Bursting and tonic discharges in two classes of reticular thalamic neurons. *J Neurophysiol* 68: 973–977.
16. Contreras D, Dossi RC, Steriade M (1993) Electrophysiological properties of cat reticular thalamic neurones in vivo. *J Physiol (Lond.)* 470:273–294.
17. Bal T, McCormick DA (1993) Mechanisms of oscillatory activity in guinea-pig nucleus reticularis thalami in vitro: a mammalian pacemaker. *J Physiol (Lond.)* 468: 669–691.
18. Brunton J, Charpak S (1997) Heterogeneity of cell firing properties and opioid sensitivity in the thalamic reticular nucleus. *Neuroscience* 78:303–307.
19. Domich L, Oakson G, Steriade M (1986) Thalamic burst patterns in the naturally sleeping cat: a comparison between cortically projecting and reticularis neurones. *J Physiol* 379:429–449.
