# Supplementary material for: Unified thalamic model generates multiple distinct oscillations with state-dependent entrainment by stimulation
Source: PLoS Comput Biol. 2017 Oct 26;13(10):e1005797. doi: 10.1371/journal.pcbi.1005797 (PMC5675460; doi:10.1371/journal.pcbi.1005797)
Supplement: S1 Table — (DOCX) [file pcbi.1005797.s002.docx]

**S1 Table.** Maximal conductance densities (mS/cm^2^) of active ionic currents in the HTC, RTC, IN and RE model cells.

|  | *I*_Na_ | *I*_DR_ | *I*_H_ | *I*_Ca/L_ | *I*_Ca/T_ | *I*_Ca/HT_ | *I*_AHP_ | *I*_CAN_ |
| --- | --- | --- | --- | --- | --- | --- | --- | --- |
| HTC | 90 | 10 | 0.01 | 0.5 | 2.1 | 3.0 | 0.3 | 0.5 |
| RTC | 90 | 10 | 0.01 | 0.3 | 2.1 | 0.6 | 0.1 | 0.6 |
| IN | 90 | 10 | 0.05 | – | – | 2.5 | 0.2 | 0.1 |
| RE | 90 | 10 | – | – | 1.3 | – | 0.2 | 0.2 |
