# Supplementary material for: Unified thalamic model generates multiple distinct oscillations with state-dependent entrainment by stimulation
Source: PLoS Comput Biol. 2017 Oct 26;13(10):e1005797. doi: 10.1371/journal.pcbi.1005797 (PMC5675460; doi:10.1371/journal.pcbi.1005797)
Supplement: S2 Table — (DOCX) [file pcbi.1005797.s003.docx]

**S2 Table.** Kinetics of gating variables for each channel implemented in the HTC, RTC, IN and RE model cells.

| Current Type | Gating Variable | $\phi_{x}$ | α_x_ or *x*_∞_ | β_x_ or τ_x_ (ms) | Ref. |
| --- | --- | --- | --- | --- | --- |
| *I*_Na_ | *p=3* | 1 | $\alpha_{m}=\frac{0.32(V-V_{SH}-13)}{1-\exp(-(V-V_{SH}-13)/4)}$  *V*_SH =_ -30 for HTC and IN  *V*_SH =_ -40 for RTC and RE | $\beta_{m}=\frac{-0.28(V-V_{SH}-40)}{1-\exp((V-V_{SH}-40)/5)}$ | [1] |
|  | *q=1* | 1 | $\alpha_{h}=0.128\exp(-(V-V_{SH}-17)/18)$ | $\beta_{h}=\frac{4}{1+\exp(-(V-V_{SH}-40)/5)}$ |  |
| *I*_DR_ | *p=4* | 0.25  (RE: 1) | $\alpha_{m}=\frac{0.032(V-V_{SH}-15)}{1-\exp(-(V-V_{SH}-15)/5)}$ | $\beta_{m}=0.5\exp(-(V-V{}_{SH}-10)/40)$ | [1] |
| *I*_H_ | *p=1* | 1 | $m_{\infty}=\frac{1}{1+\exp((V+75)/5.5)}$ | $\tau_{m}=\frac{1}{\exp(-0.086V-14.59)+\exp(0.0701V-1.87)}$ | [2] |
| *I*_Ca/L_ | *p=2* | 4.6 | $m_{\infty}=\frac{1}{1+\exp(-(V+10)/4)}$ | $\tau_{m}=0.4+\frac{0.7}{\exp(-(V+5)/15)+\exp((V+5)/15))}$ | [3] |
|  | *q=1* | 3.7 | $h_{\infty}=\frac{1}{1+\exp((V+25)/2)}$ | $\tau_{h}=300+\frac{100}{\exp(-(V+40)/9.5)+\exp((V+40)/9.5))}$ |  |
| *I*_Ca/T_ | *p=2* | 4.6 | $m_{\infty}=\frac{1}{1+\exp(-(V+62)/6.2)}$ | $\tau_{m}=0.612+\frac{1}{\exp(-(V+135)/16.7)+\exp((V+19.8)/18.2))}$ | [2] |
|  | *q=1* | 3.7 | $h_{\infty}=\frac{1}{1+\exp((V+86)/4)}$ | When V < -83 mV, $\tau_{h}=\exp((V+470)/66.6)$  When V >= -83 mV, $\tau_{h}=\exp(-(V+25)/10.5)+28$ |  |
| *I*_Ca/HT_ | *p=2* | 4.6 | $m_{\infty}=\frac{1}{1+\exp(-(V+34)/6.2)}$ | $\tau_{m}=0.612+\frac{1}{\exp(-(V+107)/16.7)+\exp((V-8.2)/18.2))}$ | [2] |
|  | *q=1* | 3.7 | $h_{\infty}=\frac{1}{1+\exp((V+58)/4)}$ | When V < -55 mV, $\tau_{h}=\exp((V+442)/66.6)$  When V >= -55 mV, $\tau_{h}=\exp(-(V-3)/10.5)+28$ |  |
| *I*_Ca/T_  (RE) | *p=2* | 6.9 | $m_{\infty}=\frac{1}{1+\exp(-(V+55)/7.4)}$ | $\tau_{m}=3+\frac{1}{\exp((V+30)/10)+\exp(-(V+105)/15))}$ | [4] |
|  | *q=1* | 3.7 | $h_{\infty}=\frac{1}{1+\exp((V+83)/5)}$ | $\tau_{m}=85+\frac{1}{\exp((V+51)/4)+\exp(-(V+410)/50))}$ |  |
| *I*_CAN_ | *p=1* | 1 | $m_{\infty}=\frac{1}{1+\exp(-(V+43)/5.2)}$ | $\tau_{m}=1.6+\frac{2.7}{\exp(-(V+55)/15)+\exp((V+55)/15))}$ | [3] |
| *I*_AHP_ | *p=1* | 1 | $m_{\infty}=\frac{48\cdot[Ca]_{i}^{2}}{48\cdot[Ca]_{i}^{2}+0.09}$ | $\tau_{m}=\frac{1}{48\cdot[Ca]_{i}^{2}+0.09}$ | [5] |

**References**

1. Bazhenov M, Timofeev I, Steriade M, Sejnowski TJ (2002) Model of thalamocortical slow-wave sleep oscillations and transitions to activated States. *J Neurosci* **22:**8691-8704.
2. Huguenard JR, McCormick DA (1992) Simulation of the currents involved in rhythmic oscillations in thalamic relay neurons. *J Neurophysiol* 68:1373–1383.
3. Inoue T, Strowbridge BW (2008) Transient activity induces a long-lasting increase in the excitability of olfactory bulb interneurons. *J Neurophysiol* 99: 187–199.
4. Huguenard JR, Prince DA (1992) A novel T-type current underlies prolonged Ca^2+^- dependent burst firing in GABAergic neurons of rat thalamic reticular nucleus. *J Neurosci* 12: 3804–3817.
5. Vijayan S, Kopell NJ (2012) Thalamic model of awake alpha oscillations and implications for stimulus processing. Proc Natl Acad Sci USA 109: 18553–18558.
