# Supplementary figures and images for: Unified thalamic model generates multiple distinct oscillations with state-dependent entrainment by stimulation
Source: PLoS Comput Biol. 2017 Oct 26;13(10):e1005797. doi: 10.1371/journal.pcbi.1005797 (PMC5675460; doi:10.1371/journal.pcbi.1005797)

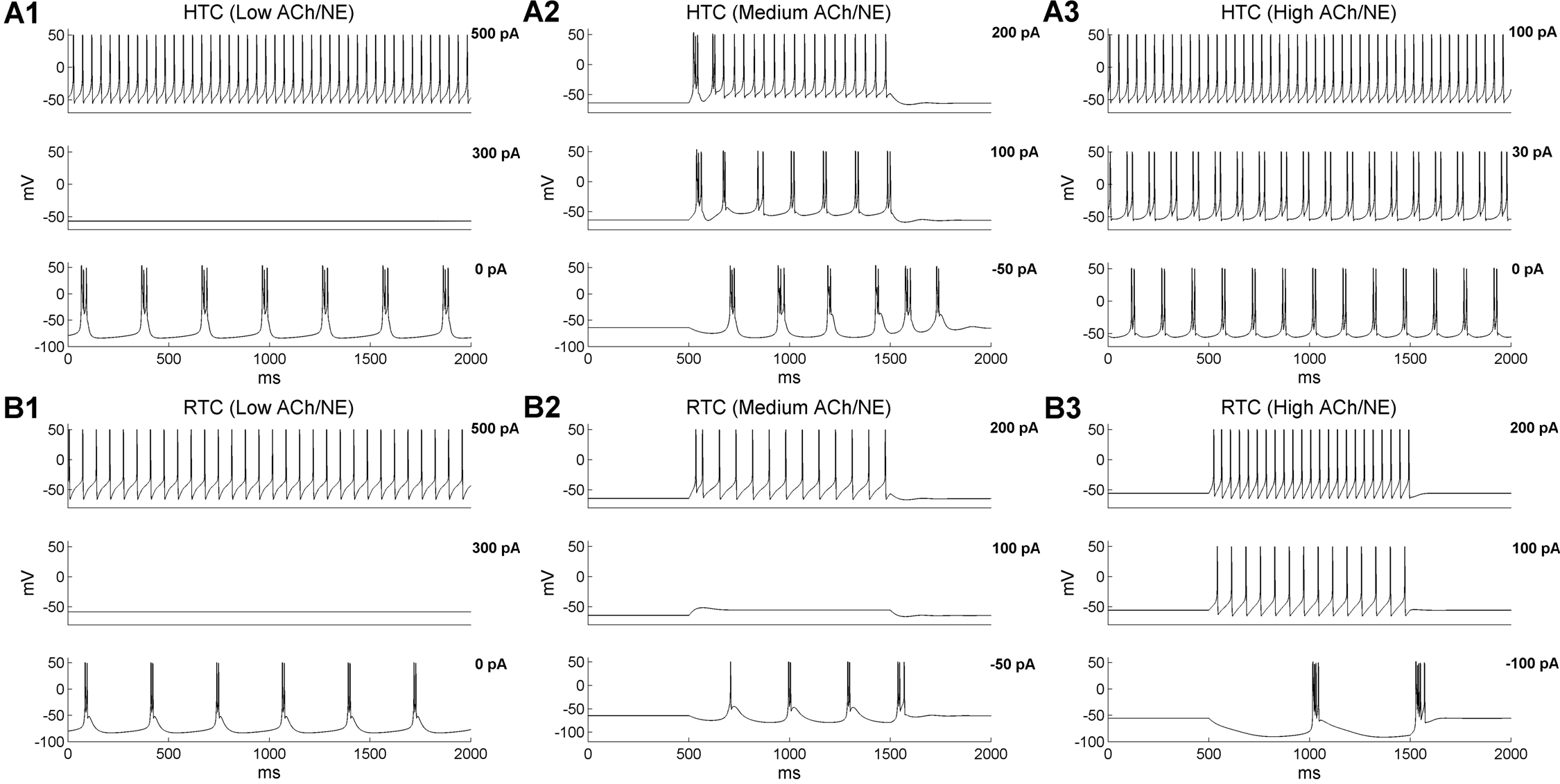

Supplement: S1 Fig — (A) Voltage responses of the HTC model cell. (A1) Voltage responses of the HTC model cell to three levels of current injection (0 pA, 300 pA and 500 pA; 0–2000 ms) in the low ACh/NE modulatory state. Note that HTC cell generates spontaneous low-threshold bursting. (A2) Voltage responses of the HTC model cell to three levels of current injection (-50 pA, 100 pA and 200 pA; 500–1500 ms) in the medium ACh/NE modulatory state. (A3) Voltage responses of the HTC model cell to three levels of current injection (0 pA, 30 pA and 100 pA; 0–2000 ms) in the high ACh/NE modulatory state. Note that HTC cell generates spontaneous high-threshold bursting. (B) Voltage responses of the RTC model cell. (B1) Voltage responses of the RTC model cell to three levels of current injection (0 pA, 300 pA and 500 pA; 0–2000 ms) in the low ACh/NE modulatory state. (B2) Voltage responses of the RTC model cell to three levels of current injection (-50 pA, 100 pA and 200 pA; 500–1500 ms) in the medium ACh/NE modulatory state. (B3) Voltage responses of the RTC model cell to three levels of current injection (-100 pA, 100 pA and 200 pA; 500–1500 ms) in the high ACh/NE modulatory state. For both HTC and RTC cells, gKL = 0.035 mS/cm2 in the low ACh/NE modulatory state; gKL = 0.01 mS/cm2 in the medium ACh/NE modulatory state and gKL = 0.0 mS/cm2 in the high ACh/NE modulatory state. (TIF) [file pcbi.1005797.s004.tif]

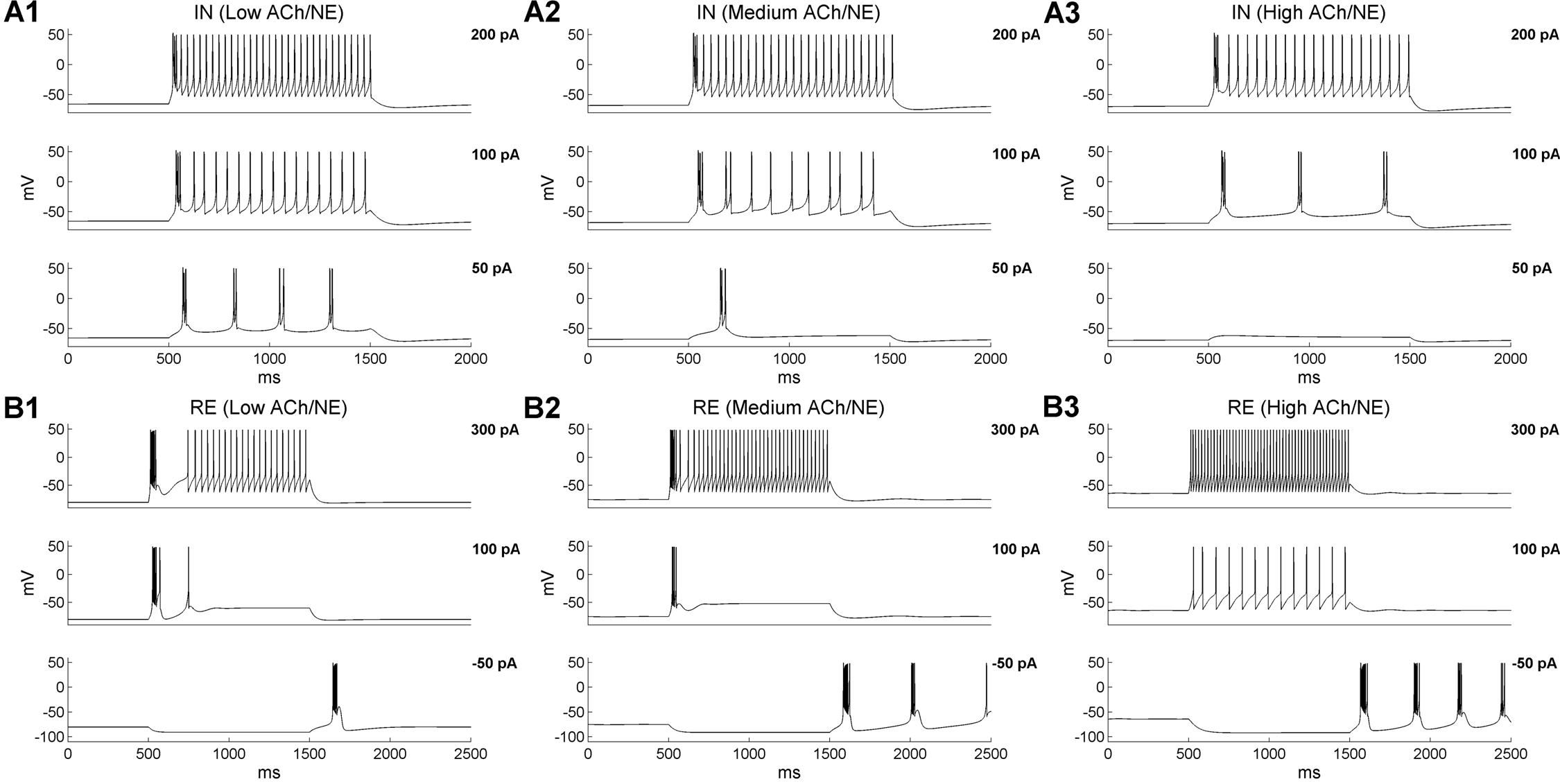

Supplement: S2 Fig — (A) Voltage responses of the IN model cell. (A1) Voltage responses of the IN model cell to three levels of current injection (50 pA, 100 pA and 200 pA; 500–1500 ms) in the low ACh/NE modulation state. (A2) As (A1), but in the medium ACh/NE modulation state. (A3) As (A1), but in the high ACh/NE modulation state. For the low ACh/NE modulation state, gKL = 0.01 mS/cm2; for the medium ACh/NE modulation state, gKL = 0.015 mS/cm2; and for the high ACh/NE modulation state, gKL = 0.02 mS/cm2. (B) Voltage responses of the RTC model cell. (B1) Voltage responses of the RE model cell to three levels of current injection (-50 pA, 100 pA and 300 pA; 500–1500 ms) in the low ACh/NE modulation state. (B2) As (B1), but in the medium ACh/NE modulation state. (B3) As (B1), but in the high ACh/NE modulation state. For the low ACh/NE modulation state, gKL = 0.03 mS/cm2; for the medium ACh/NE modulation state, gKL = 0.02 mS/cm2; and for the high ACh/NE modulation state, gKL = 0.01 mS/cm2. (TIF) [file pcbi.1005797.s005.tif]

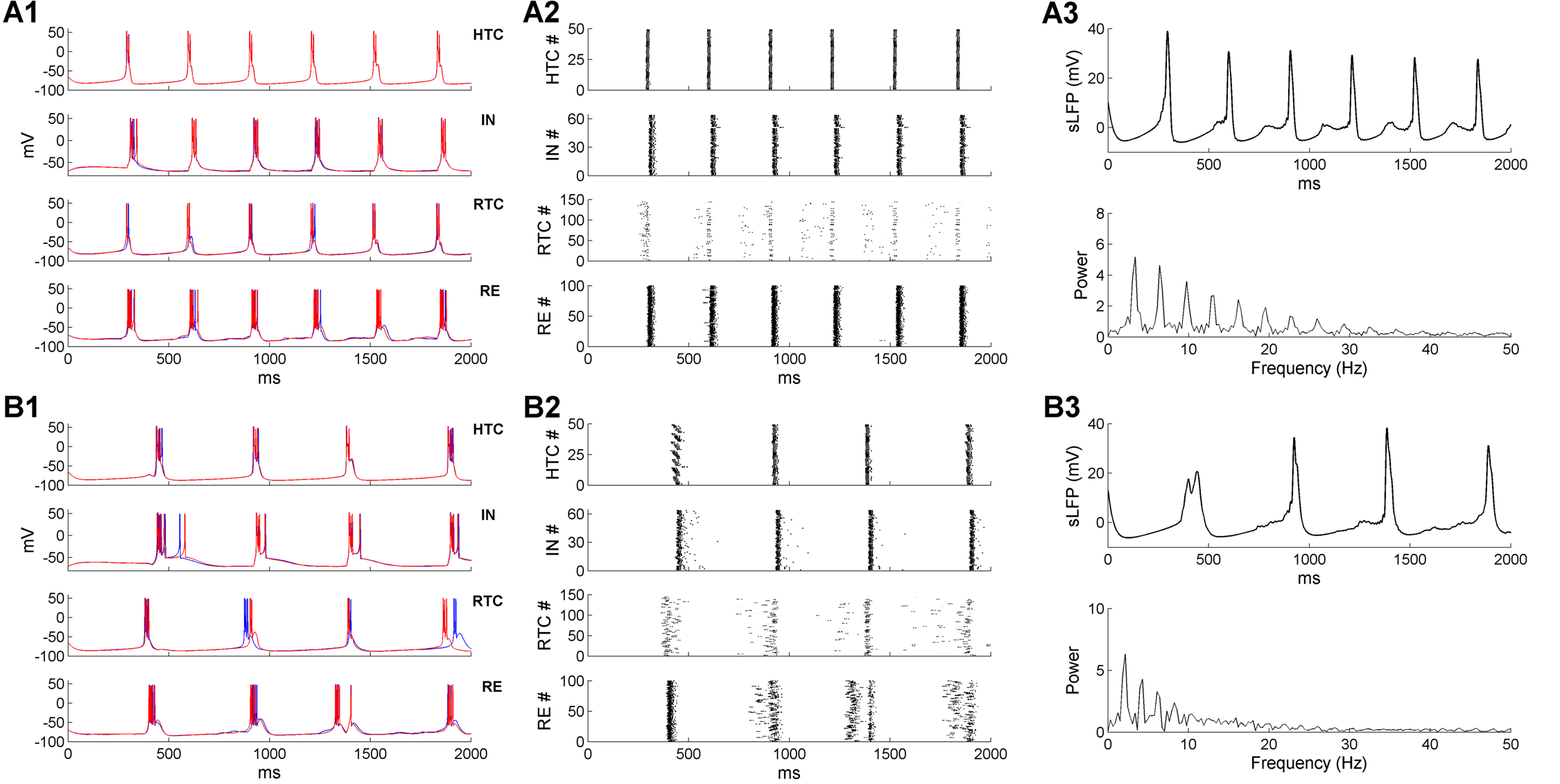

Supplement: S3 Fig — (A) Delta oscillation frequency is reduced to about 3 Hz (controls: 3.7 Hz) when the potassium leak conductance in TC cells slightly increases to 0.037 mS/cm2 (controls: 0.035 mS/cm2). (A1) Membrane voltage of representative HTC, IN, RTC and RE neurons. (A2) Spike rastergrams of HTC, IN, RTC and RE cells. (A3) Simulated LFP (top) with associated frequency power spectrum (bottom). (B) Delta oscillation frequency is reduced to about 2 Hz (controls: 3.7 Hz) when the regular leak conductance in TC cells is reduced substantially to 0.001 mS/cm2 (controls: 0.01 mS/cm2), the potassium leak conductance in TC cells increases to 0.04 mS/cm2 (controls: 0.035 mS/cm2), and the inactivation time constant of the low-threshold T-type Ca2+ current (ICa/T) increases 25%. (B1) Membrane voltage of representative HTC, IN, RTC and RE neurons. (B2) Spike rastergrams of HTC, IN, RTC and RE cells. (B3) Simulated LFP (top) with associated frequency power spectrum (bottom). (TIF) [file pcbi.1005797.s006.tif]

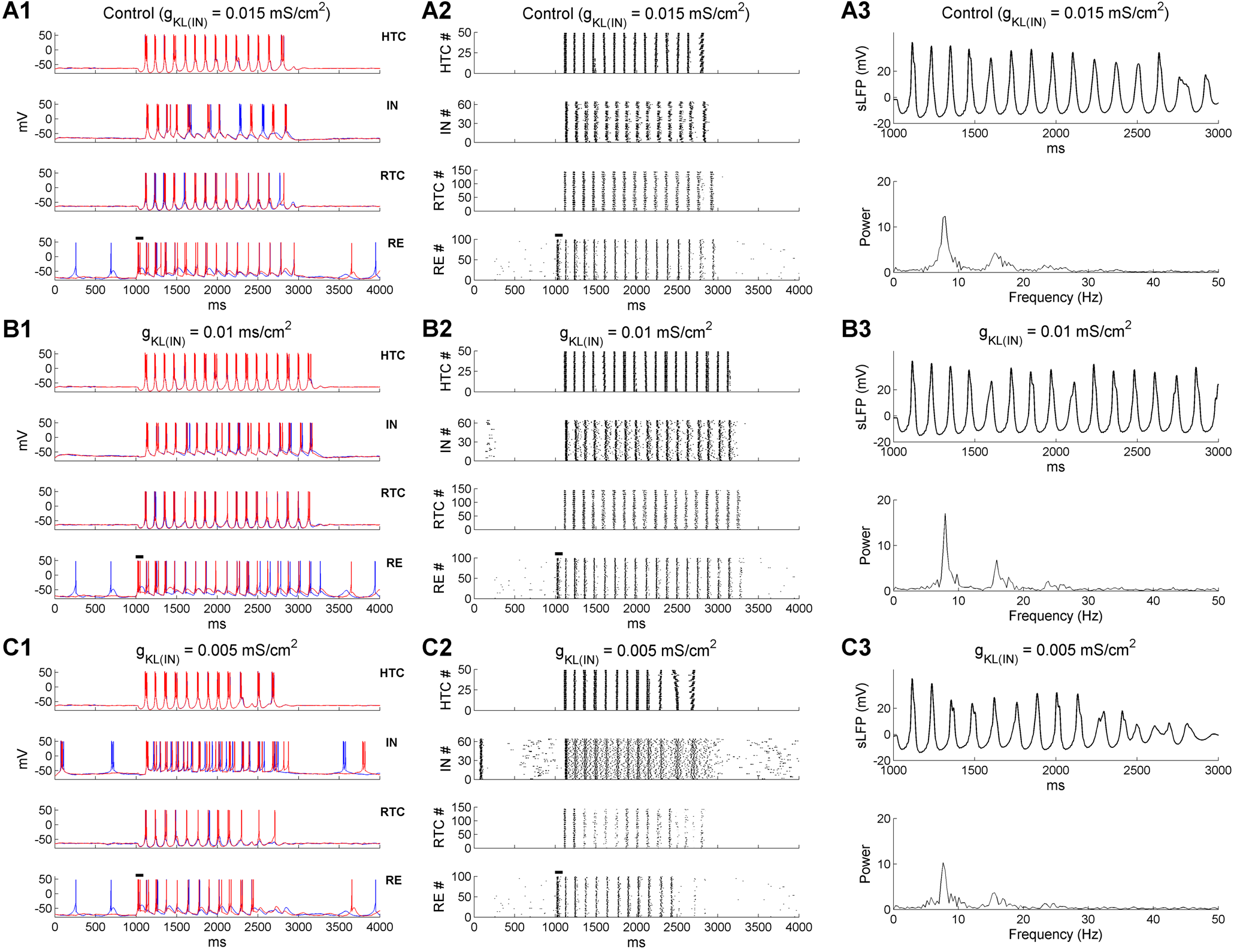

Supplement: S4 Fig — (A) Spindle oscillations during the control condition when the NE modulatory effect on INs is neglected (gKL = 0.015 mS/cm2). (A1) Membrane voltages of representative HTC, IN, RTC and RE cells. (A2) Spike rastergrams of HTC, IN, RTC and RE cells. (A3) Simulated LFP (top) with associated frequency power spectrum (bottom). (B) Spindle oscillations when the NE modulatory effect on INs counteracts the effect of ACh (gKL = 0.01 mS/cm2). (B1) Membrane voltages of representative HTC, IN, RTC and RE cells. (B2) Spike rastergrams of HTC, IN, RTC and RE cells. (B3) Simulated LFP (top) with associated frequency power spectrum (bottom). (C) Spindle oscillations when the NE modulatory effect on INs overcomes the effect of ACh (gKL = 0.005 mS/cm2). (C1) Membrane voltages of representative HTC, IN, RTC and RE cells. (C2) Spike rastergrams of HTC, IN, RTC and RE cells. (C3) Simulated LFP (top) with associated frequency power spectrum (bottom). (TIF) [file pcbi.1005797.s007.tif]

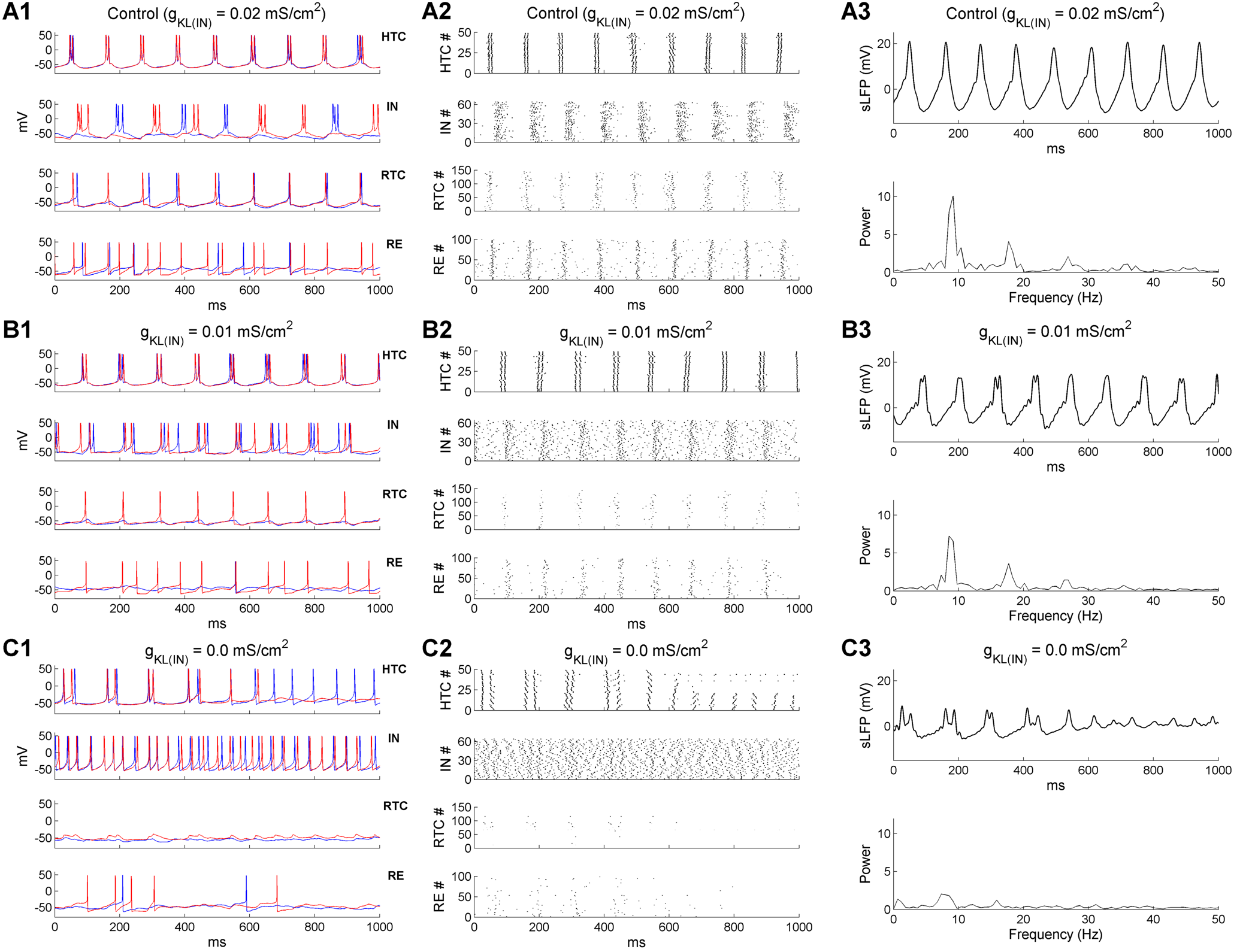

Supplement: S5 Fig — (A) Alpha oscillations during the control condition when the NE modulatory effect on INs is neglected (gKL = 0.02 mS/cm2). (A1) Membrane voltages of representative HTC, IN, RTC and RE cells. (A2) Spike rastergrams of HTC, IN, RTC and RE cells. (A3) Simulated LFP (top) with associated frequency power spectrum (bottom). (B) Alpha oscillations when the NE modulatory effect on INs counteracts the effect of ACh (gKL = 0.01 mS/cm2). (B1) Membrane voltages of representative HTC, IN, RTC and RE cells. (B2) Spike rastergrams of HTC, IN, RTC and RE cells. (B3) Simulated LFP (top) with associated frequency power spectrum (bottom). (C) Alpha oscillations when the NE modulatory effect on INs overcomes the effect of ACh (gKL = 0.0 mS/cm2). (C1) Membrane voltages of representative HTC, IN, RTC and RE cells. (C2) Spike rastergrams of HTC, IN, RTC and RE cells. (C3) Simulated LFP (top) with associated frequency power spectrum (bottom). (TIF) [file pcbi.1005797.s008.tif]

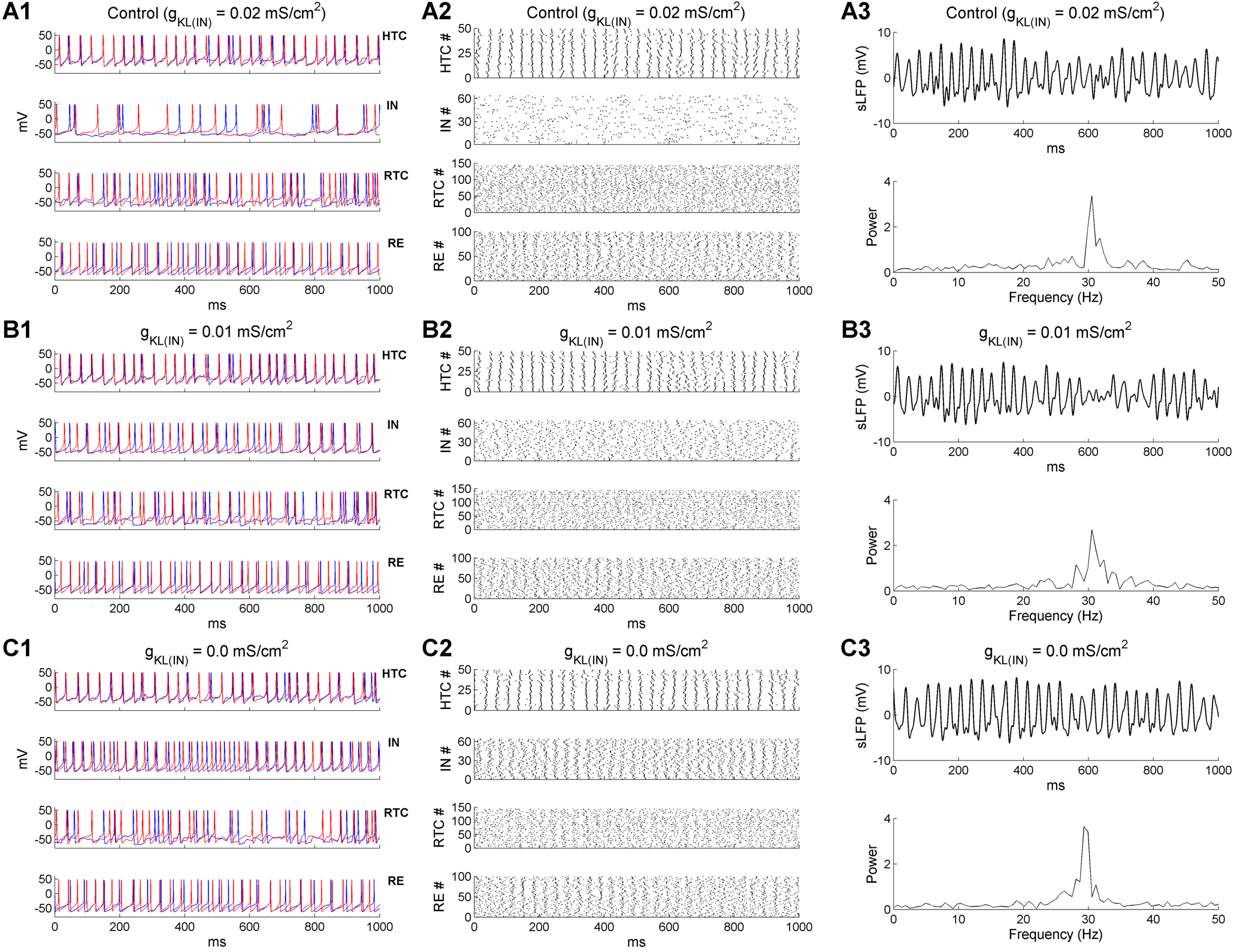

Supplement: S6 Fig — (A) Gamma oscillations during the control condition when the NE modulatory effect on INs is neglected (gKL = 0.02 mS/cm2). (A1) Membrane voltages of representative HTC, IN, RTC and RE cells. (A2) Spike rastergrams of HTC, IN, RTC and RE cells. (A3) Simulated LFP (top) with associated frequency power spectrum (bottom). (B) Gamma oscillations when the NE modulatory effect on INs counteracts the effect of ACh (gKL = 0.01 mS/cm2). (B1) Membrane voltages of representative HTC, IN, RTC and RE cells. (B2) Spike rastergrams of HTC, IN, RTC and RE cells. (B3) Simulated LFP (top) with associated frequency power spectrum (bottom). (C) Gamma oscillations when the NE modulatory effect on INs overcomes the effect of ACh (gKL = 0.0 mS/cm2). (C1) Membrane voltages of representative HTC, IN, RTC and RE cells. (C2) Spike rastergrams of HTC, IN, RTC and RE cells. (C3) Simulated LFP (top) with associated frequency power spectrum (bottom). (TIF) [file pcbi.1005797.s009.tif]

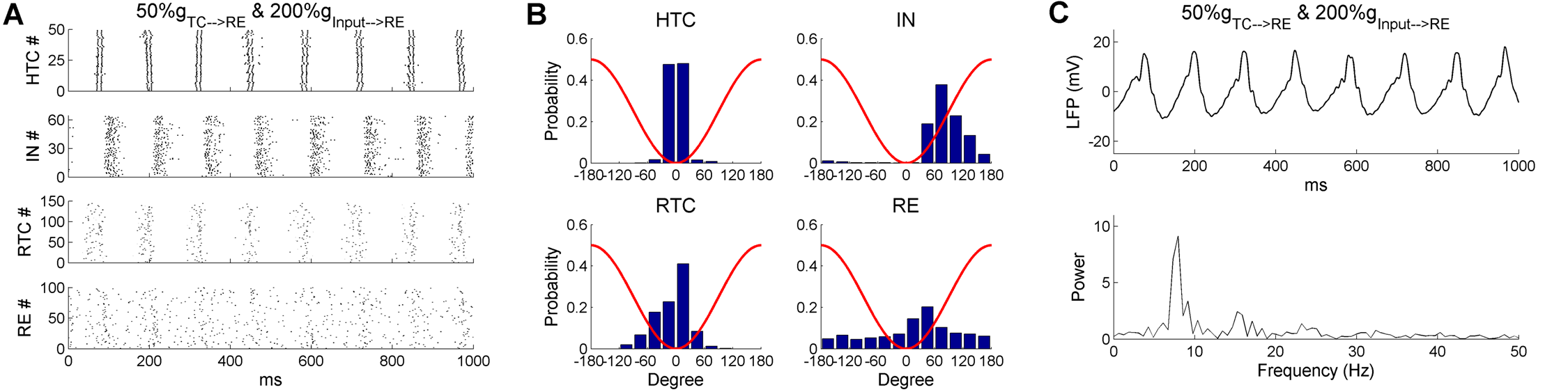

Supplement: S7 Fig — (A) Spike rastergrams of HTC, IN, RTC and RE cells when the TC→RE synaptic strength is reduced by 50% (AMPA: from 4 nS to 2 nS; NMDA: from 2 nS to 1 nS) and the random input strength to RE cells increases twofold (from 1.5 nS to 3 nS). (B) Distribution of spike phase relative to sLFP peaks for HTC, IN, RTC and RE cells under the same parameter changes as (A). (C) Simulated LFP (top) with associated frequency power spectrum (bottom) under the same parameter changes as (A). (TIF) [file pcbi.1005797.s010.tif]

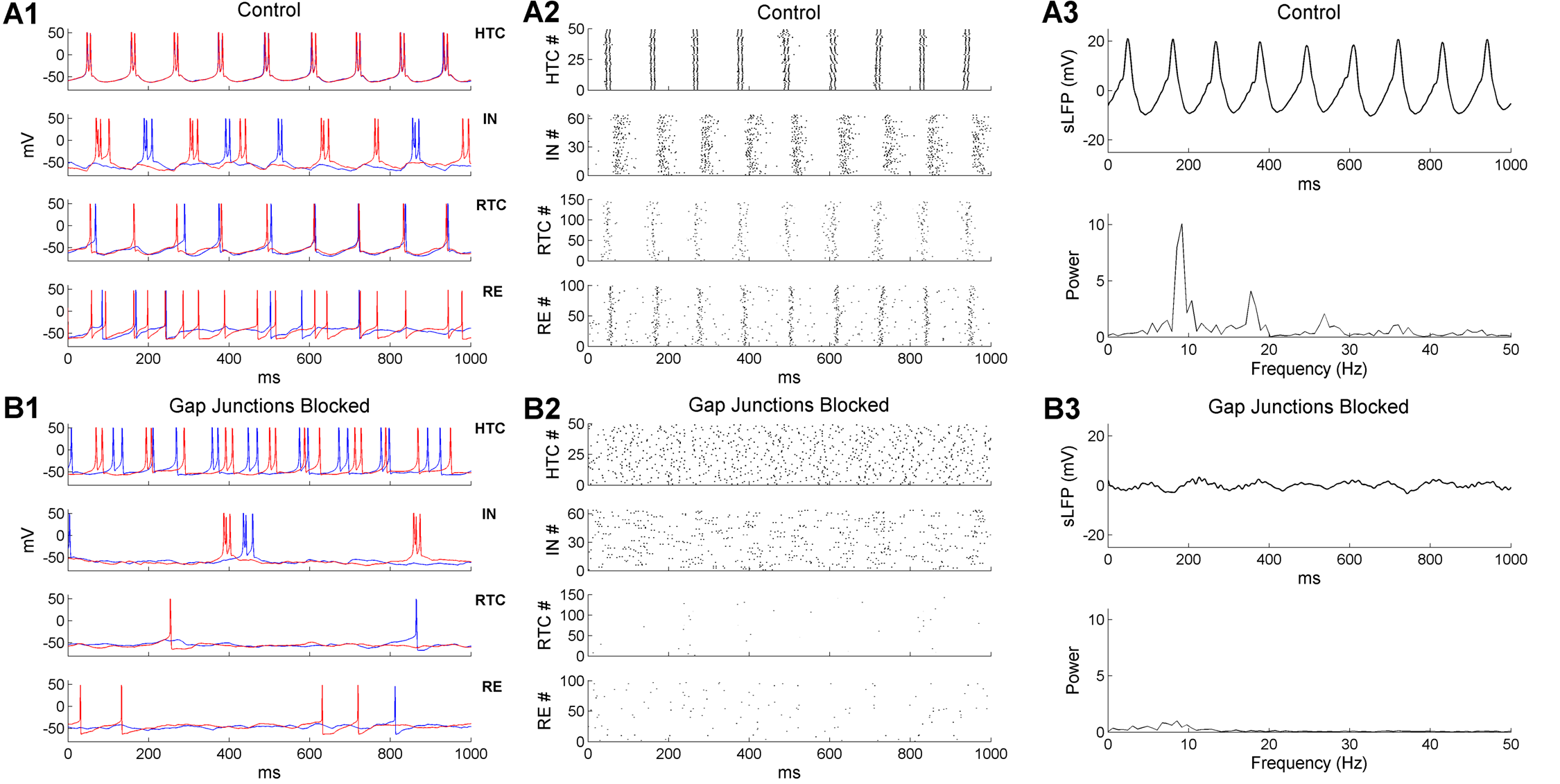

Supplement: S8 Fig — (A) Thalamic network activities with intact gap junctions. (A1) Membrane voltages of two representative HTC, IN, RTC and RE cells each in the control case. (A2) Spike rastergrams of HTC, IN, RTC and RE cells in the control case. (A3) Simulated LFP (top) with associated frequency power spectrum (bottom) in the control case. (B) Thalamic network activities without gap junctions. (B1-B3) As (A1-A3), but when gap junctions among TC cells (HTC-HTC & HTC-RTC) are blocked. (TIF) [file pcbi.1005797.s011.tif]

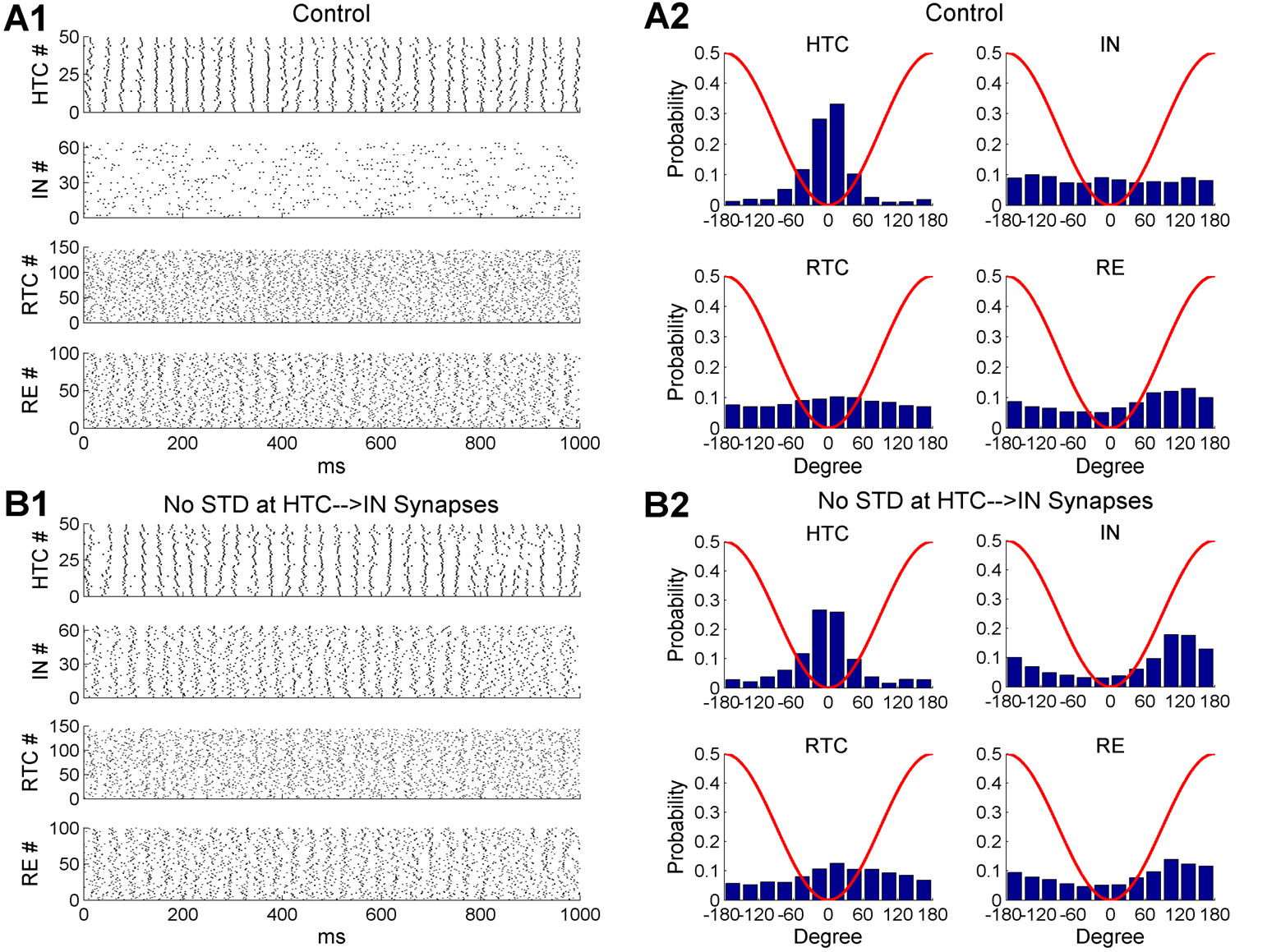

Supplement: S9 Fig — (A) INs are not phase locked to the γ rhythm in the control case. (A1) Spike rastergrams of HTC, IN, RTC and RE cells. (A2) Distribution of spike phases relative to sLFP peaks for HTC, IN, RTC and RE cells. (B) INs are phase locked to the γ rhythm when the STD at HTC→IN synapses is blocked. (B1-B2) As (A1-A2), but when the STD at HTC→IN synapses is blocked. (TIF) [file pcbi.1005797.s012.tif]

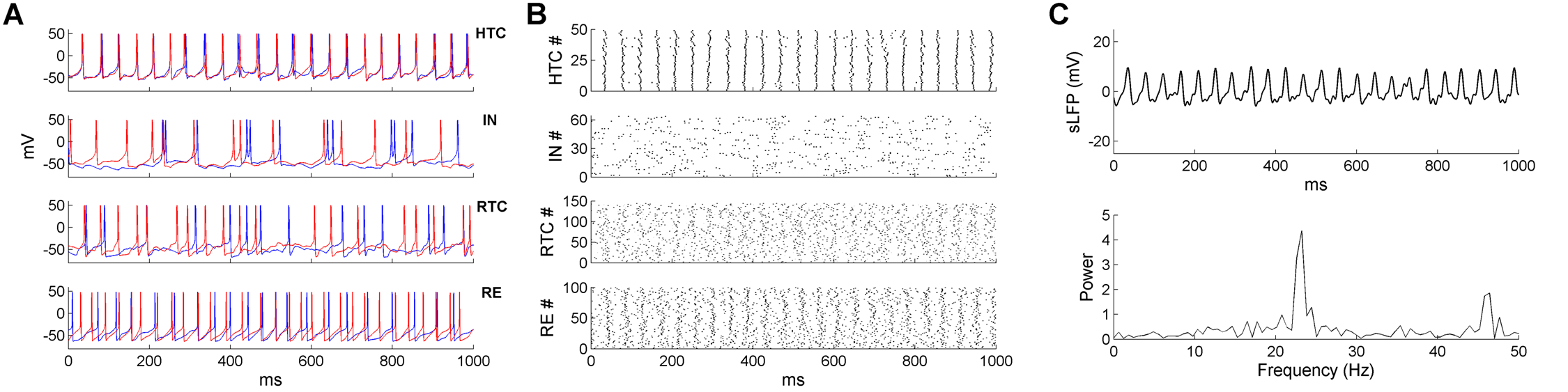

Supplement: S10 Fig — (A) Membrane voltages of two representative HTC, IN, RTC and RE cells each. (B) Spike rastergrams of HTC, IN, RTC and RE cells. (C) Simulated LFP (top) with associated frequency power spectrum (bottom). (TIF) [file pcbi.1005797.s013.tif]

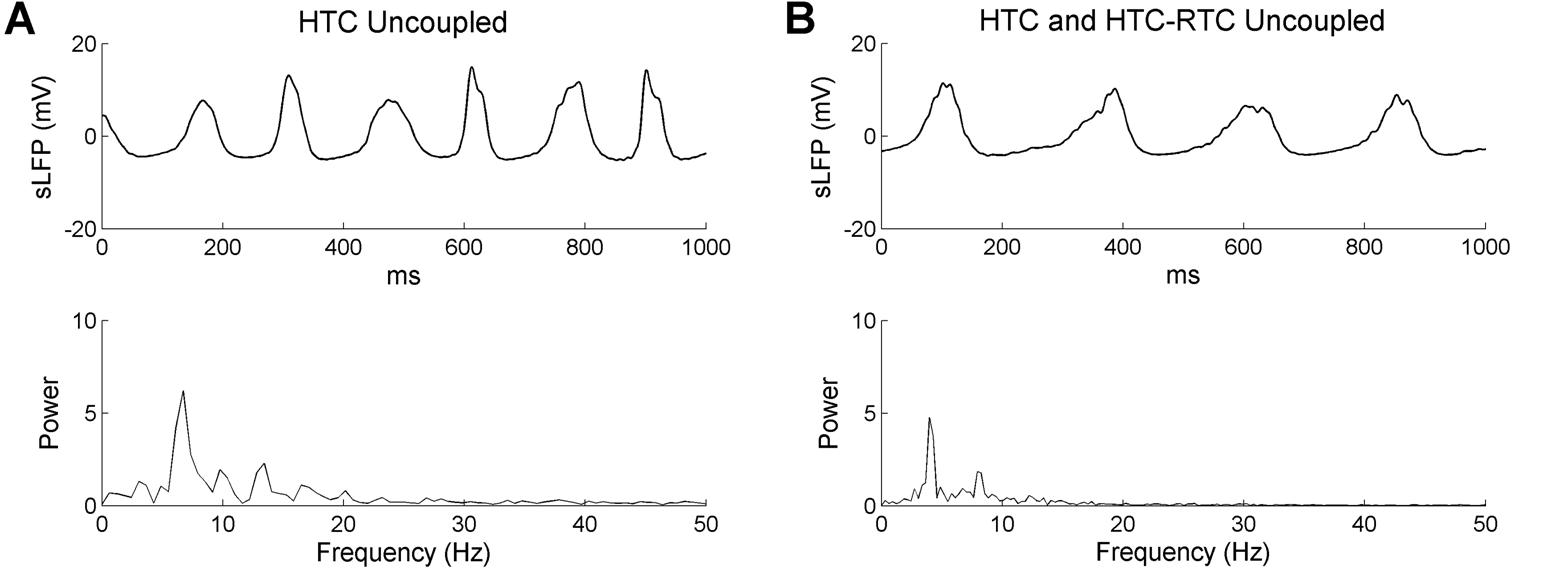

Supplement: S11 Fig — (A) Simulated LFP (top) with associated frequency power spectrum (bottom) when the gap junctions among HTC cells are blocked. (B) As (A), but when both HTC-HTC and HTC-RTC gap junctions are blocked. (TIF) [file pcbi.1005797.s014.tif]

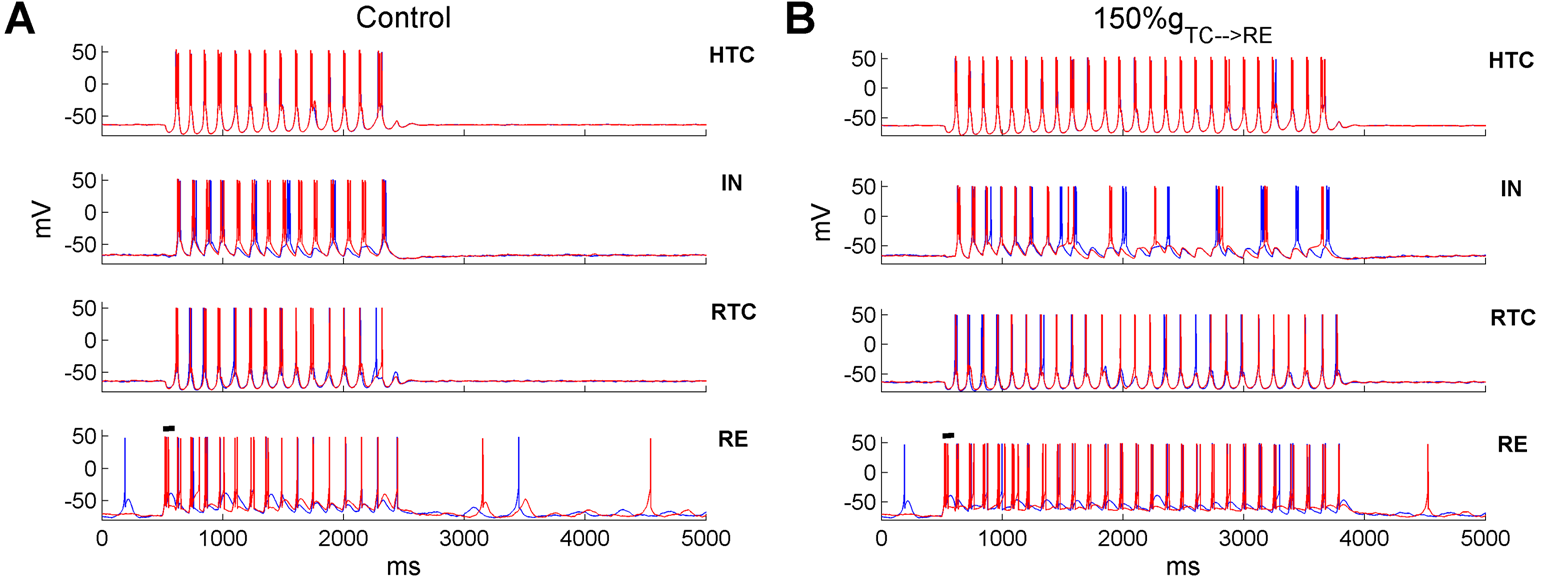

Supplement: S12 Fig — (A) Membrane voltages of two representative HTC, IN, RTC and RE cells each in the control condition. (B) Membrane voltage of two representative HTC, IN, RTC and RE cells each when the excitatory TC→RE synaptic weight increases to 150% of its default value (AMPA: from 4 nS to 6 nS; NMDA: from 2 nS to 3 nS). The horizontal bar in the bottom panel indicates the injection of a transient current input (100 ms × 100 pA) into RE neurons to trigger spindle oscillations. (TIF) [file pcbi.1005797.s015.tif]

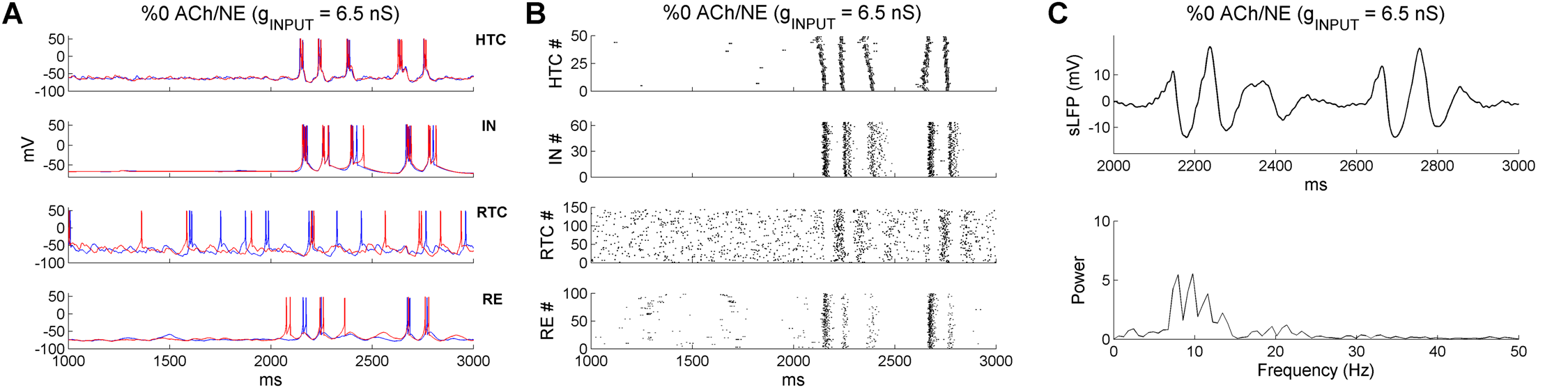

Supplement: S13 Fig — (A) Membrane voltages of two representative HTC, IN, RTC and RE cells each. (B) Spike rastergrams of HTC, IN, RTC and RE cells. (C) Simulated LFP (top) with associated frequency power spectrum (bottom). (TIF) [file pcbi.1005797.s016.tif]

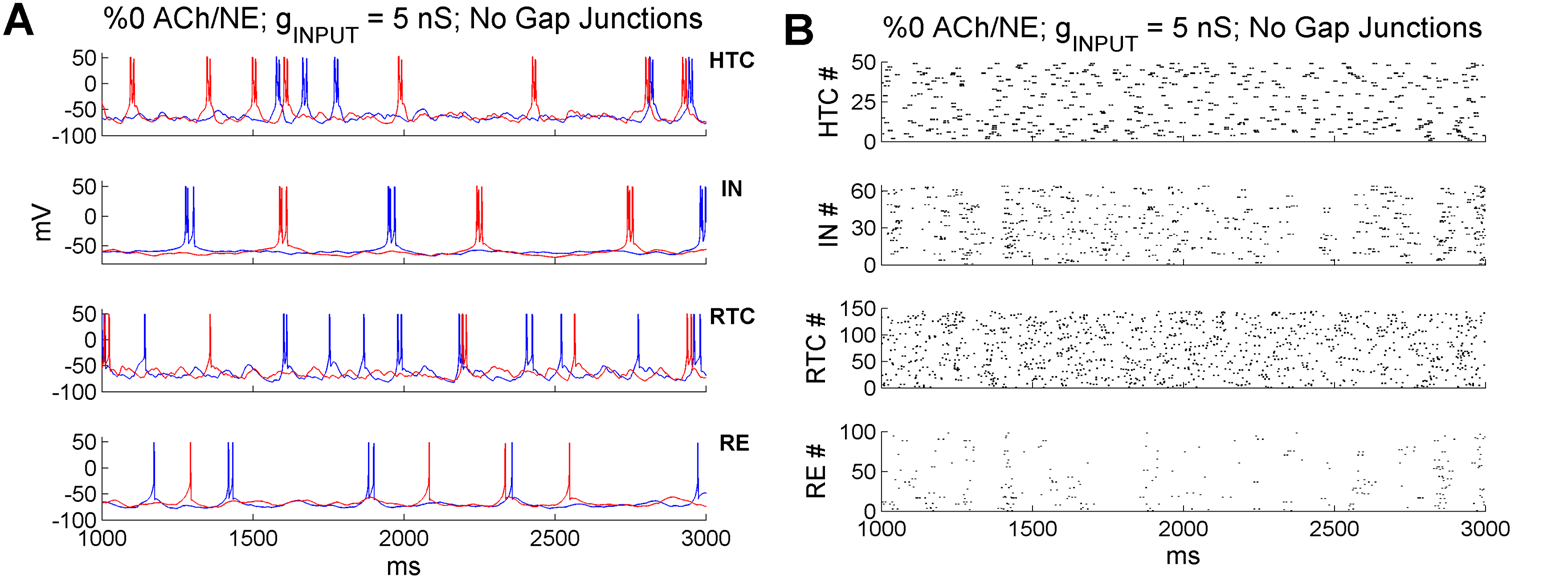

Supplement: S14 Fig — (A) Membrane voltages of two representative HTC, IN, RTC and RE cells each. (B) Spike rastergrams of HTC, IN, RTC and RE cells. (TIF) [file pcbi.1005797.s017.tif]

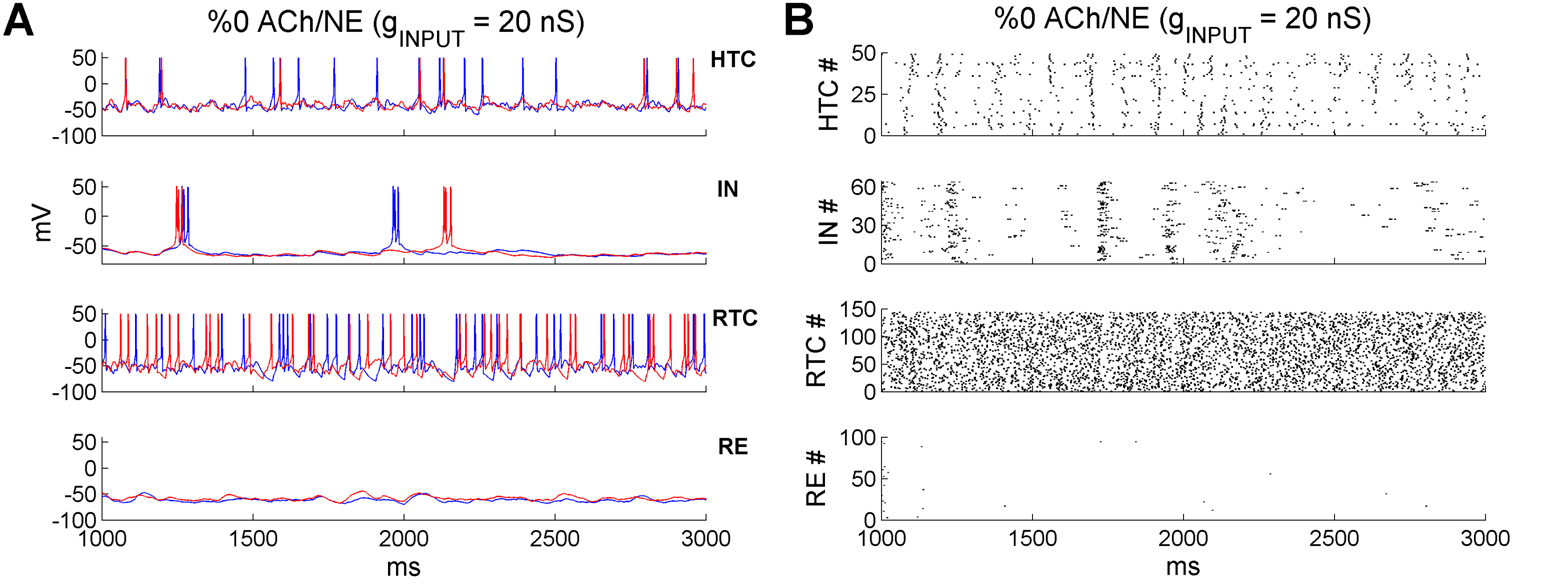

Supplement: S15 Fig — (A) Membrane voltages of two representative HTC, IN, RTC and RE cells each. (B) Spike rastergrams of HTC, IN, RTC and RE cells. (TIF) [file pcbi.1005797.s018.tif]

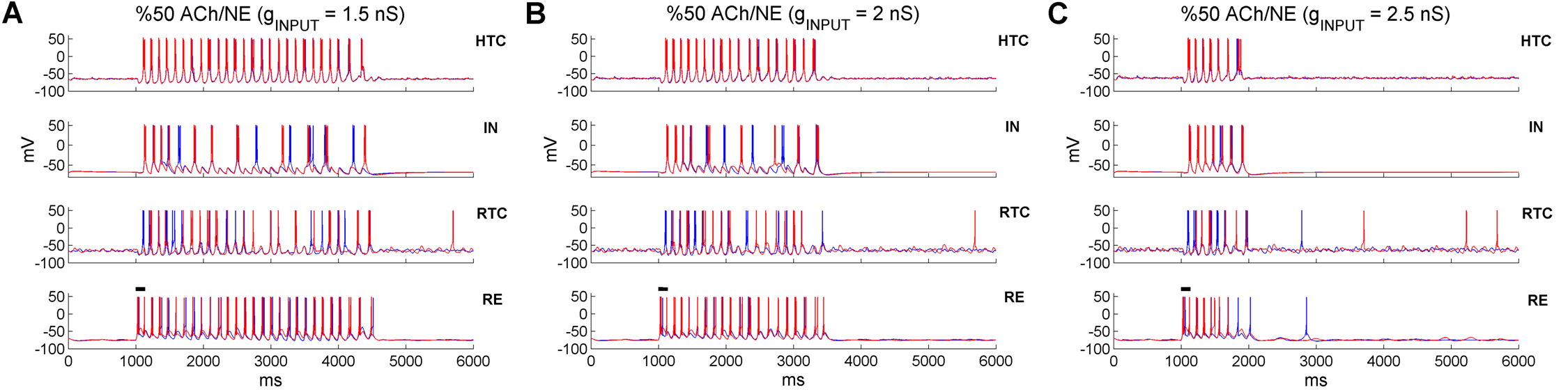

Supplement: S16 Fig — (A) Membrane voltages of two representative HTC, IN, RTC and RE cells each when gInput = 1.5 nS. (B) Membrane voltages of two representative HTC, IN, RTC and RE cells each when gInput = 2.0 nS. (C) Membrane voltages of two representative HTC, IN, RTC and RE cells each when gInput = 2.5 nS. (TIF) [file pcbi.1005797.s019.tif]

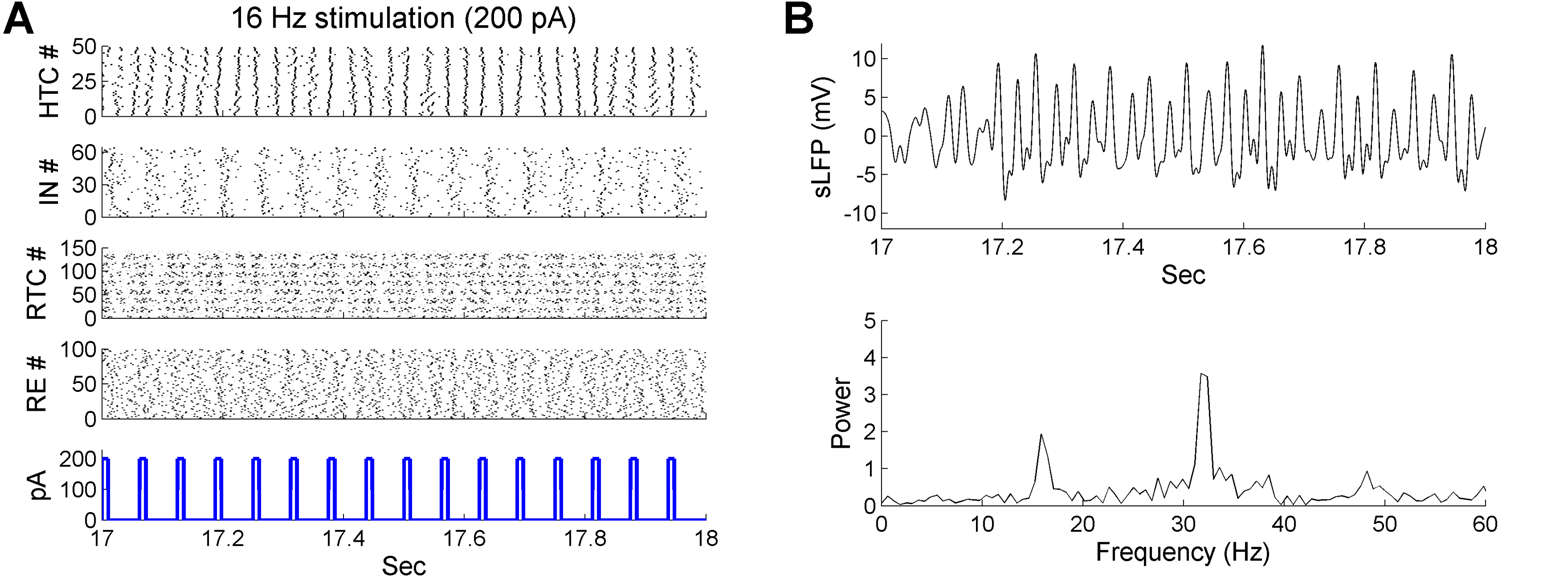

Supplement: S17 Fig — (A) Top four panels: spike rastergrams of HTC, IN, RTC and RE cells; bottom panel: stimulation waveform. (B) Simulated LFP (top) with associated frequency power spectrum (bottom). (TIF) [file pcbi.1005797.s020.tif]

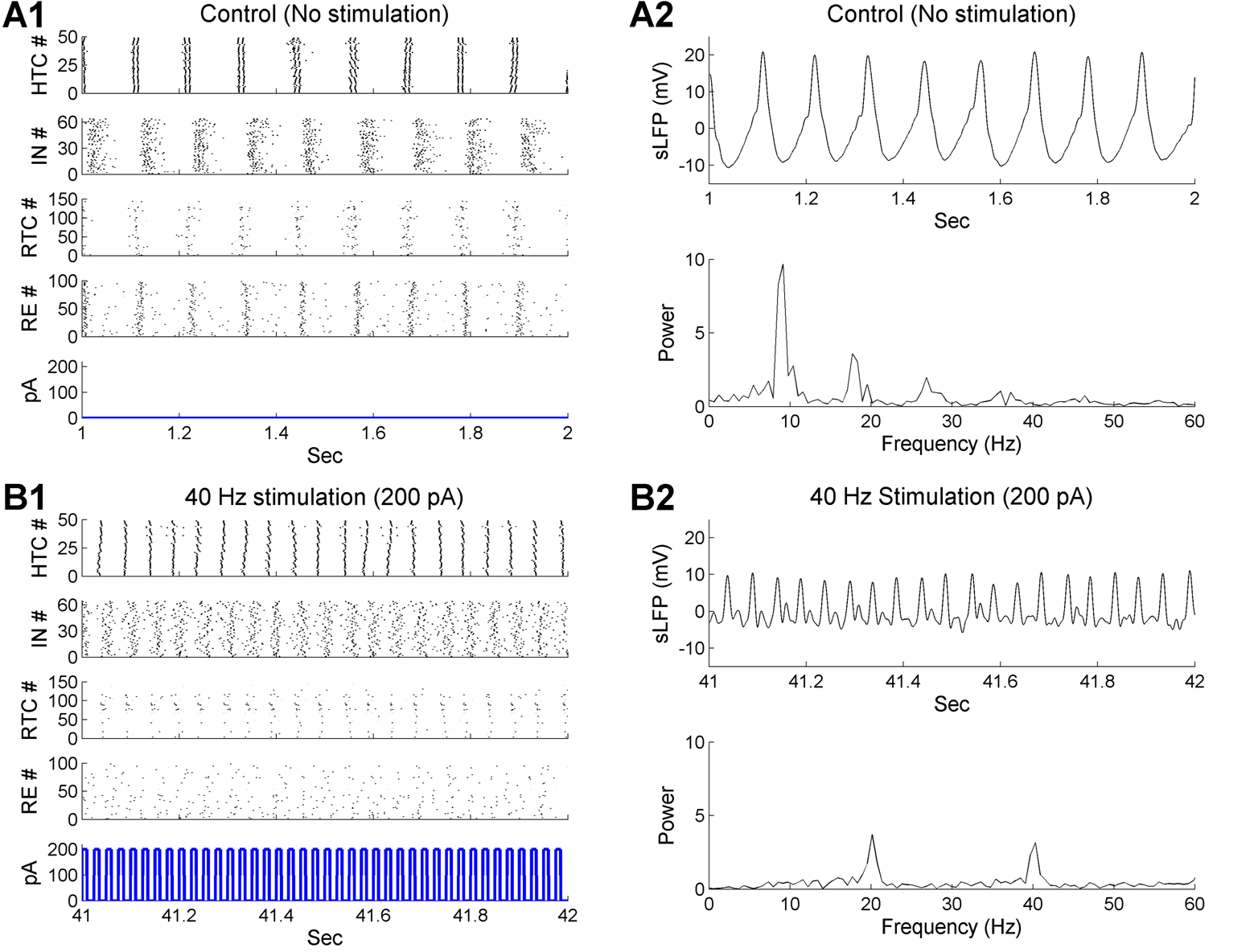

Supplement: S18 Fig — (A) Thalamic network activity during α oscillations without stimulation. (A1) Spike rastergrams of HTC, IN, RTC and RE cells (top four panels). (A2) Simulated LFP (top) with associated frequency power spectrum (bottom). (B) Thalamic network activity during 40 Hz stimulation of α oscillations. (B1) Spike rastergrams of HTC, IN, RTC and RE cells (top four panels) with stimulation waveform (bottom panel). (B2) Simulated LFP (top) with associated frequency power spectrum (bottom). The stimulation amplitude is 200 pA (0.2 nA). (TIF) [file pcbi.1005797.s021.tif]
